# An annotated checklist of Coccinellidae (Insecta, Coleoptera) with eight new records from the Kingdom of Saudi Arabia

**DOI:** 10.3897/zookeys.1006.59123

**Published:** 2020-12-21

**Authors:** Amin Al Ansi, Areej A. Alkhalaf, Hassan Fadl, Iftekhar Rasool, Hathal Al Dhafer

**Affiliations:** 1 King Saud University Museum of Arthropods, Plant Protection Department, College of Food and Agriculture Sciences, King Saud University, Riyadh, Saudi Arabia King Saud University Riyadh Saudi Arabia; 2 Biology Department, College of Science, Princess Nourah bint Abdulrahman University, Riyadh, Saudi Arabia Princess Nourah bint Abdulrahman University Riyadh Saudi Arabia; 3 Entomology Departments, Faculty of Science, Ain Shams University, Cairo, Egypt Ain Shams University Cairo Egypt

**Keywords:** Checklist, ladybird beetles, new records, Saudi Arabia, zoogeographical distribution

## Abstract

The members of Coccinellidae are among the most important biological control agents being used throughout the world. The objective of this study was to provide the current scenario of this family in Saudi Arabia. Collection surveys of ladybird individuals were conducted throughout Saudi Arabia. A total of 5400 specimens of Coccinellidae were collected belonging to 51 species in 19 genera and seven tribes: Sticholotidini (2 genera / 10 species), Coccidulini (1/3), Scymnini (4/24), Diomini (1/1), Hyperaspidini (1/3), Chilocorini (3/8), Noviini (1/3), Coccinellini (8/11), Epilachnini (2/2). One genus, *Clitostethus*, and eight species and subspecies, *Cheilomenes lunata lunata* (Fabricius, 1775), *Clitostethus arcuatus* (Rossi, 1794), *Nephus ornatulus* Korschefsky, 1931, *N. nigricans* (Weise, 1879), *Pharoscymnus fleischeri* (Weise, 1883), *Novius yemenensis* Raimundo & Fürsch, 2006, Scymnus (Scymnus) scapuliferus Mulsant, 1850, and *Stethorus endrodyi* Fürsch, 1970, are reported as new records to Saudi Arabia. Two endemic species, *Scymnus agrumi* and *S. arabicus*, were recorded. This study also describes the geographical distribution for each species and the diagnostic characters for new records.

## Introduction

The members of Coccinellidae comprise one of the most important biological control agents being used throughout the world (Obrycki and Kring 1998). This family deserves its own superfamily Coccinelloidea in the suborder Polyphaga (Robertson et al. 2015) and represents approximately 6000 described species in 360 genera worldwide, of which approximately 90% are considered as predators (Vandenberg 2002; Hesler 2009; Magro et al. 2010). Monitoring the diversity of coccinellid species is crucial for integrated pest management programs and for incorporating the biological control of numerous arthropod pests. Coccinellids are relatively well known and commonly known as ladybird beetles in Australia, South Africa, and Great Britain and ladybugs in North America (Magro et al. 2010; Roy and Migeon 2010). These beetles can be found in various habitats, ranging from highly urbanized areas to the types of agro and natural ecosystems (Leather et al. 1999).

Before this study, a total of 56 species were reported from Saudi Arabia in 20 genera and 12 tribes (Kapur 1959; Shalaby 1962; Beccari 1971; Martin 1972; Fürsch 1979; Talhouk 1982; Walker and Pittaway 1987; Fürsch 1989; Raimundo and van Harten 2000; Kovář 2007). The first study on ladybird beetles of Saudi Arabia was conducted by Kapur (1959) with an initial record of 32 species from southwestern Arabia (southwestern KSA and Yemen). Subsequently, Hesler (1962) listed three species, *Exochomus flavipes* Thunberg, *Epilachna hirta* Thunberg, and *Thea 16-notata* (Mulsant 1850), from KSA. Beccari (1971) listed 10 species, *Adonia variegata* (Goeze, 1777), *Chilocorus bipustulatus* (L., 1858), *Cheilomenes propinqua vicina* Mulsant, 1850; *Coccinella 7-punctata* (L., 1758); *C. 11-punctata* (L., 1758); *Epilachna chrysomelina* (F., 1775); *E. hirta* (Thunberg, 1781); *Exochomus flavipes* (Thunberg, 1781); *Scymnus punctillum* (Weise, 1891); and *Thea 16-notata* (Mulsant, 1850), from KSA. Fürsch (1979) provided an identification key for 35 species, describing two new species, *Nephus wittmeri* Fürsch, 1979; and *Pharoscymnus arabicus* Fürsch, 1979, and one new subspecies, *N. hiekei riyadhensis*. Walker and Pittaway (1987) provided comprehensive comments, colour photographs, distributional information, and maps for four species of coccinellids occurring in Eastern Arabia. Fürsch (1989) also examined the groups of the species-rich genus *Scymnus* of the Arabian Peninsula and recorded only six species from KSA, including two new species, *S. arabicus* Fürsch, 1989, and *S. luxorensis* Fürsch, 1989. Later, Raimundo and van Harten (2000) provided the description of the new species *Serangium buettikeri* Fürsch, 2000, from KSA and Yemen. The family Coccinellidae of the Palaearctic region was catalogued by Kovář (2007), who listed 41 species from KSA.

Several researchers have been exploring the diversity of Coccinellidae in the adjacent countries of Saudi Arabia. Yemen represents the highest diversity of coccinellids with 85 species (Raimundo and van Harten 2000; Raimundo et al. 2006). In the United Arab Emirates, 23 species have been reported to date (Raimundo et al. 2007; Gillett 2009), including a phytophagous species, *Henosepilachna elaterii orientalis* (Zimmermann, 1936). In Jordan, seven species of coccinellids, *C. bipustulatus*, *Pharoscymnus ovoideus* Sicard, 1929, *Cheilomenes propinqua vicina*, *E. quadripustulatus*, *Scymnus subvillosus* (Goeze, 1777), *S. rubromaculatus* (Goeze, 1777), and *Oenopia conglobata* (Linnaeus, 1758), have been recorded (Awameleh et al. 2009).

Syria has 20 recorded species of Coccinellidae (Khalil and Mourad 2005, 2006). In Egypt, 59 coccinellid species have been recorded (Alfieri 1976; Al Akkad 1979). In Palestine and adjacent areas, 71 species of coccinellids have been reported (Halperin et al. 1995). In the present study, the objective was to provide the current status of the family Coccinellidae for the KSA, the largest land mass of the Arabian Peninsula, by surveying the species of this family in selected regions of KSA, to identify the Coccinellidae specimens in the King Saud University Museum of Arthropods (KSMA) and Arabian National Museum of Arthropods, Ministry of Agriculture (ANMA), and the specimens that were collected during the present investigation.

## Materials and methods

**Figure 1.**
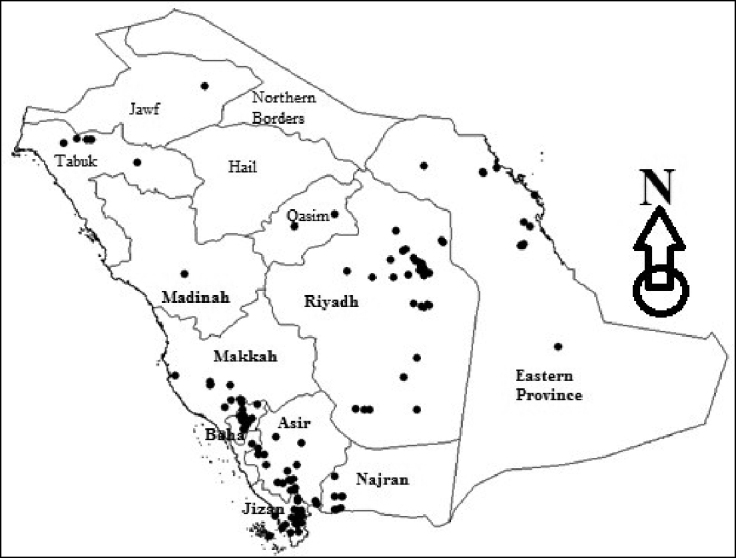
Map of Saudi Arabia indicating collection localities.

Collection surveys were conducted throughout the KSA (Figure 1) using beating sheets, malaise traps, sweep net, light traps, and sucking traps and by handpicking. Additional materials were examined from KSMA, ANMA, both in Riyadh, Saudi Arabia. Adult coccinellid beetles of large size were mounted immediately whenever possible using #2 and #3 pins (Monarch Brand from www.bioquip.com). Small-sized coccinellid beetles were mounted on small pieces of stiff paper, and some were preserved in 70% ethanol for dissection purpose and were deposited in KSMA.

For the dissection of genitalia, the method of Davis (2009) was followed with some modifications. Specimens were first relaxed by soaking in warm water for 10–15 min. The abdomen was separated from the body under a stereomicroscope using fine needles or forceps with small-sized tips. After extracting the genitalia (male genitalia or spermatheca) from the abdomen, they were placed in 10% KOH solution for 5–10 min depending on the amount of tissues. After removing from the KOH solution, the genitalia were placed in water to neutralize any KOH for 2–5 min, glued onto a small card using yellow glue (# 1160 shellac gel), and placed underneath the specimen. Original illustrations were completed in addition to production of digital images using an Auto-Montage version 1.2 with Micropublisher 5.0 RTV digital camera from the Q Imaging Company, Canada, installed on a Leica microscope type MZ 12.5 (Leica Company, Germany). All images were enhanced using Adobe Photoshop CS5.

The following information is provided for each species: current combinations of names and their synonymies, brief diagnosis (for new records only), remarks, material examined, country and world distribution (after Kovář 2007, for abbreviations see Table 1), and published records in KSA. The data for the material examined are arranged as follows: name of the province, followed by locality, the coordinates, elevation above sea level, the date of collection (months in Roman numerals), the collection methods (handpicking (HP), light trap (LT), malaise trap (MT), pitfall trap (PT), beating sheet (BS), sweeping net (SN), sucking (SU), and sticky traps (ST)), collector names, and the number of examined specimens, followed by sex (♂, ♀ for identified sex and ex(s) for unidentified sex). A brief diagnosis of each new record species was added. New record species are marked with an asterisk. Geographical coordinates were obtained from GPS for specimens that were collected by the authors or obtained from the label data under the investigated individuals for specimens that were collected in KSMA or ANMA.

Species were identified using original description and all available literature reports such as Fürsch (1979; 1989), Raimundo and van Harten (2000), Raimundo et al. (2006), and Raimundo et al. (2007) and by examining the reference collection from ANMA and KSMA. Taxonomy on the tribe and genus levels follows Nedvěd (2020). Detailed accounts for each species and their ecological notes are also provided.

**Table 1.** Acronyms for the countries are given following Kovář (2007).

**Acronyms of countries**			
**Europe**			
AB: Azerbaijan	EN: Estonia	KZ: Kazakhstan	RO: Romania
AL: Albania	FA: Faeroe Island	LS: Liechtenstein	RU: Russia
AN: Andorra	FI: Finland	LT: Lithuania	SK: Slovakia
AR: Armenia	FR: France	LU: Luxembourg	SL: Slovenia
BE: Belgium	GB: Great Britain	MA: Malta	SP: Spain (Gibraltar)
BH: Bosnia Herzegovina	GE: Germany	MC: Macedonia	SR: Svalbard (Spitzbergen)
BU: Bulgaria	GG: Georgia	MD: Moldavia	ST: Russia (South European Territory)
BY: Belarus	GR: Greece	NL: The Netherlands	SV: Sweden
CR: Croatia	HU: Hungary	NR: Norway	SZ: Switzerland
CT: Russia (Central European Territory)	IC: Iceland	NT: Russia (North European Territory)	TR: Turkey
CZ: Czech Republic	IR: Ireland	PL: Poland	UK: Ukraine
DE: Denmark	IT: Italy (Sardinia, Sicily, San Marino)	PT: Portugal	YU: Yugoslavia (Serbia, Montenegro)
**North Africa**			
AG: Algeria	EG: Egypt	MO: Morocco	TU: Tunisia
CI: Canary Islands	LB: Libya	MR: Madeira Archipelago	
**Asia**			
AE: Arab Emirates	PAL: Palestine	NC: North Korea	SI: Egypt (Sinai)
AF: Afghanistan	JA: Japan	OM: Oman	SY: Syria
BA: Bahrain	JO: Jordan	PA: Pakistan	TD: Tajikistan
CH: China	KI: Kyrgyzstan	QA: Qatar	TM: Turkmenistan
CY: Cyprus	KU: Kuwait	RU: Russia	TR: Turkey
FS: Russia (Far East)	KZ: Kazakhstan	SA: Saudi Arabia	UZ: Uzbekistan
IN: Iran	MG: Mongolia	SC: South Korea	YE: Yemen including Socotra
IQ: Iraq	NP: Nepal	SD: India (Sikkim, Darjeeling District)	

### Zoogeographical regions

Acronyms for Zoogeographical regions used throughout text:

**AFR** Afrotropical region

**AUR** Australian region

**ORR** Oriental region

**NAR** Nearctic region

**NTR** Neotropical region

**PAR** Palaearctic region

## Results

In total, 5400 specimens including both fresh collections from KSA and preserved materials in KSMA and ANMA were examined, of which 51 species were recognized in this investigation, including eight new records to the country (Table 2). Two endemic species *Scymnus agrumi* and *S. arabicus* were recorded.

**Table 2.** Checklist of Coccinellidae species recorded from the Kingdom of Saudi Arabia.

Species	Reference
*Brumoides adenensis* Fürsch, 1987***	Kovář 2007
*Brumoides nigrosuturalis* (Kapur, 1959)	Talhouk 1982
*Bulaea lividula bocandei* Mulsant, 1850	Kapur 1959; Fürsch 1979; Kovář 2007; Abdel-Dayem et al. 2017
*Cheilomenes lunata lunata* (F., 1775) *	Current study
*Cheilomenes lunata yemenensis* Fürsch, 1989	Raimundo et al. 2006 reported from YE; Kovář 2007
*Cheilomenes propinqua vicina* (Mulsant, 1850)	Kapur 1959; Beccari 1971; Martin 1972; Fürsch 1979; Talhouk 1982; Kovář 2007
*Chilocorus bipustulatus* (L., 1758)	Beccari 1971; Martin 1972; Talhouk 1982; Kovář 2007
*Chilocorus distigma* Klug, 1835	Kovář 2007
*Clitostethus arcuatus* Rossi, 1794 **	Current study
*Coccinella septempunctata* (L., 1758)	Beccari 1971; Martin 1972; Fürsch 1979; Talhouk 1982; Walker and Pittaway 1987; Kovář 2007; Abdel-Dayem et al. 2017
*Coccinella undecimpunctata menetriesi* Mulsant, 1850	Beccari 1971; Martin 1972; Talhouk 1982; Kovář 2007; Abdel-Dayem et al. 2017
*Diomus rubidus* (Motschulsky, 1837)	Fürsch 1979; Kovář 2007; Abdel-Dayem et al. 2017
*Harmonia axyridis* (Pallas, 1773)	Biranvand et al. 2019
*Chnootriba elaterii orientalis* (Zimmermann, 1936)	Kapur 1959; Beccari 1971; Martin 1972; Fürsch 1979; Walker and Pittaway 1987; Kovář 2007
*Henosepilachna hirta* (Thunberg, 1781)	Shalaby 1962; Beccari 1971
Hippodamia (Adonia) variegata (Goeze, 1777)	Beccari 1971; Talhouk 1982; Kovář 2007; El-Hawagry et al. 2013; Abdel-Dayem et al. 2017
*Hyperaspis polita* Weise, 1855	Kovář 2007
*Hyperaspis pumila pumila* Mulsant, 1850	Kovář 2007
*Hyperaspis vinciguerrae* Capra, 1929	Martin 1972; Fürsch 1979; Abu-Thuraya 1982; Talhouk 1982; Kovář 2007; Abdel-Dayem et al. 2017
Nephus (Bipunctatus) conjunctus (Wollaston, 1870)***	Fürsch 1979; Kovář 2007
Nephus (Bipunctatus) wittmeri Fürsch, 1979***	Fürsch 1979; Kovář 2007; Abdel-Dayem et al. 2017
Nephus (Geminosipho) arcuatus Kapur, 1959	Fürsch 1979; Kovář 2007
Nephus (Geminosipho) fenestratus (Sahlberg, 1913)***	Fürsch 1979; Kovář 2007
Nephus (Nephus) crucifer Fleischer, 1900	Fürsch 1979; Kovář 2007
Nephus (sidis) hiekei (Fürsch 1965)	Martin 1972; Fürsch 1979; Abu-Thuraya 1982; Talhouk 1982; Kovář 2007
*Nephus* (sidis) levaillanti (Mulsant, 1850)	Martin 1972; Abu-Thuraya 1982
*Nephus ornatulus* Korscefsky, 1931*	Current study
*Nephus nigricans* (Weise, 1879)*	Current study
*Oenopia oncina* (Olivier, 1808)***	Kovář 2007
Parexochomus (Exochomus) nigripennis Erichson, 1843	Shalaby 1962; Beccari 1971; Martin 1972; Fürsch 1979; Talhouk 1982; Kovář 2007
Parexochomus (Exochomus) nigromaculatus (Goeze, 1777)	Raimundo et al. 2006 reported from YE
*Parexochomus pubescens* (Kuster, 1848)	Kapur 1959; Fürsch 1979; Kovář 2007; Abdel-Dayem et al. 2017
*Parexochomus sjoestedti* (Weise, 1910)***	Kovář 2007
*Pharoscymnus arabicus* Fürsch,1979***	Fürsch 1979; Kovář 2007
*Pharoscymnus c-luteum* (Sicard, 1907)	Fürsch 1979; Kovář 2007
*Pharoscymnus fleischeri* (Weise, 1883)*	Current study
*Pharoscymnus numidicus* (Pic, 1900)	Fürsch 1979; Kovář 2007
*Pharoscymnus ovoideus* Sicard, 1929	Martin 1972; Abu-Thuraya 1982
*Pharoscymnus pharoides* (Marsuel, 1868)***	Kovář 2007
*Pharoscymnus setulosus* (Chevrolat, 1861)	Martin 1972; Fürsch 1979; Abu-Thuraya 1982; Kovář 2007
*Pharoscymnus smirnovi* Dobzhanskiy, 1927	Kovář 2007
*Pharoscymnus tristiculus* Sicard, 1907	Abu-Thuraya 1982
*Psyllobora bisoctonotata* (Mulsant, 1850)	Shalaby 1962; Beccari 1971; Fürsch 1979; Talhouk 1982; Kovář 2007
*Novius argodi* Sicard, 1909	Martin 1972; Abu-Thuraya 1982
*Novius cardinalis* (Mulsant, 1850)	Abu-Thuraya 1982
*Novius yemenensis* Raimundo & Fürsch, 2006*	Current study
Scymnus (Pullus) agrumi Fürsch, 1970	Fürsch 1970; Martin 1972; Abu-Thuraya 1982; Talhouk 1982; Kovář 2007
Scymnus (Pullus) arabicus Fürsch, 1989***	Fürsch 1989; Kovář 2007
Scymnus (Pullus) auritus Thunberg, 1795***	Kovář 2007
Scymnus (Pullus) ebneri Weise, 1926	Martin 1972; Abu-Thuraya 1982; Talhouk 1982
Scymnus (Pullus) latemaculatus Motschulsky, 1858	Fürsch 1979
Scymnus (Pullus) luxorensis Fürsch, 1989	Fürsch 1989; Abdel-Dayem et al. 2017
Scymnus (Pullus) subvillosus (Goeze, 1777)	Fürsch 1979; Kovář, 2007; Abdel-Dayem et al. 2017
Scymnus (Pullus) syriacus (Marsuel, 1868)	Martin 1972; Talhouk 1982; Fürsch 1989; El-Hawagry et al. 2013; Abdel-Dayem et al. 2017
Scymnus (Pullus) yemenensis Kapur, 1959	Fürsch 1979; Fürsch 1989; Kovář 2007; Abdel-Dayem et al. 2017
Scymnus (Scymnus) interruptus (Goeze, 1777)	
Scymnus (Scymnus) nubilus Mulsant, 1850	Fürsch 1979; Kovář 2007; Abdel-Dayem et al. 2017
Scymnus (Scymnus) scapuliferus Mulsant, 1850*	Current study
*Serangium buettikeri* Fürsch, 2000	Fürsch 2000; Kovář 2007
*Stethorus gilvifrons* (Mulsant, 1850)	Martin 1972; Fürsch 1979; Abu-Thuraya 1982; Talhouk 1982; Kovář 2007
*Stethorus endrodyi* Fürsch,1970*	Current study
*Tetrabrachys arabicus* Kapur, 1948***	Kapur 1948; Fürsch 1979; Kovář 2007
*Tetrabrachys minutus* (Pic, 1903)***	Kapur 1948; Kovář 2007
*Tetrabrachys tenebrosus* (Weise, 1910)***	Kapur 1948; Kovář 2007
*Xanthadalia effusa rufescens* (Mulsant, 1850)***	Kovář 2007

Species with single marked asterisk * are new species record; species marked with (**) are new genera records; and species with (***) are found in old literature but not recorded in current work.

### Coccinellinae Latreille, 1807

#### Chilocorini Mulsant, 1846

***Brumoides* Chapin, 1965**

##### Brumoides adenensis

Taxon classificationAnimaliaColeopteraCoccinellidae

Fürsch, 1987

E468A724-82BE-512E-850F-479375C02A3A

Brumoides adenensis Fürsch, 1987: 44.

###### Local distribution.

This species was reported from KSA by Kovář (2007), but not collected during the present study.

###### World distribution.

**Asia**: AE, IN, SA, and YE; **AFR** (Kovář 2007).

##### Brumoides nigrosuturalis

Taxon classificationAnimaliaColeopteraCoccinellidae

(Kapur, 1959)

FAD32E66-D4A9-568B-96C3-7174556F1A0B

Brumus nigrosuturalis Kapur, 1959: 293.

###### Material examined.

**Asir**: Al Majardah, Wadi Yabah, 19°16.27'N, 41°48.46'E, 411 m, 2.VI.2012, BS, Al Ansi, A., 1♂1♀; Al Majardah Thalooth Al Mandhar, Wadi Baqrah, 18°47.57'N, 42°01.12'E, 433 m, 4.XI.2013, HP, Abdel-Dayem, M., 1ex; 12.X.2013, HP, El Torkey, A., 1ex; 12.X.2013, SN, Khan, S., 1ex; Maraba Wadi Ramlan, 17°47.18'N, 42°22.95'E, 180 m, 10.II.2016, SU, Al Ansi, A., 1ex; Maraba, Wadi Itwad, 17°48.25'N, 42°21.64'E, 149 m, 8.II.2016, SU, Al Ansi, A., 4exs; 8.II.2016, SN, Fadl, H., 1ex; Maraba, Wadi Reem, 17°52.55'N, 42°16.66'E, 136 m, 9.II.2016, SN, Fadl, H., 2exs; **Jizan**: Ahd Al Masareha Rd., 17°02.28'N, 42°52.38'E, 11.III.2010, LT, Al Dhafer, H. and El Gharbawy, A., 1♂; Aiban, Wadi Dafa, 17°22.50'N, 43°04.53'E, 870 m, 12.XI.2012, SN, Al Dhafer, H., 1♀; Sabya-Hurub Rd., 17°16.94'N, 42°17.54'E, 97 m, 24.V.2012, SN, Al Ansi, A., 2♂; Aiban, Haqu Fifa, 17°07.95'N, 43°02.59'E, 253 m, 11.XI.2012, BS, Fadl, H. and Abdel-Dayem, M., 1♂2♀; Jizan-Abu Arish Rd, 17°04.25'N, 42°47.05'E, 24.II.2015, SN, Al Harbi, M., 4exs; Jizan-Addarab Wadi Baiz, 17°37.56"N, 42°22.24"E, 249 m, 24.II.2015, HP, Al Harbi, M., 2ex.

###### Local distribution.

This species was collected from southwest of KSA, Asir, and Jizan and was previously reported by Talhouk (1982) from Jizan.

###### World distribution.

**Asia**: SA and YE; **AFR** (Kovář 2007).

#### *Chilocorus* Leach, 1815

##### Chilocorus bipustulatus

Taxon classificationAnimaliaColeopteraCoccinellidae

(Linnaeus, 1758)

A9A7DBF2-AE09-5F60-9A04-964BCA73C97C

Coccinella bipustulata Linnaeus, 1758: 367.

###### Remarks.

This species is known as the biocontrol agent of the scale insect *Parlatoria blanchardi* (Targioni Tozzetti 1892), a date palm pest, and *Aonidiella orientalis* (Newstead 1894), a pest of *Citrus* spp. (Martin 1972), and is widely distributed throughout the world. In the present study, its adults were collected from date palm orchards on the grasses and shrubs under the palm tree and in the agroecosystem on the grasses.

###### Material examined.

old collection from ANMA, **Riyadh**: Ad Diriyah, Education farm, 24°40'N, 46°35'E, 26.III.1975, 1♂, hand written label (date palm orchard); Wadi Hanifah, 24°45'N, 46°36'E, 26.I.1977, Al Shaqaty, M., ANMA, 1ex; Riyadh, 24°43.01'N, 46°38.65'E, 4.III.1979, Sarhan, K. and Shalaby, F., 29exs, hand written label [grasses]; V.1966, ANMA, 1ex.

###### Local distribution.

This species was found in Riyadh region and listed by Martin (1972) and reported by Beccari (1971) from Riyadh and by Talhouk (1982) from Riyadh and Eastern region.

###### World distribution.

**Asia**: AF, CY, FE, GAN, IN, IQ, PAL, JO, KI, KZ, LE, MG, SA, SI, SY, TR, UZ, and XIN; **Europe**: AB, AL, AR, AU, AZ, BE, BH, BY, CZ, CT, DE, EN, FI, FR, GB, GE, GG, GR, HU, IR, IT, LA, LT, LU, MA, MC, MD, NL, NR, NT, PL, PT, RO, SK, SL, SP, ST, SV, SZ, TR, UK, and YU; **North Africa**: AG, EG, LB, MO, MR, TU; AFR, and NAR (Kovář 2007).

##### Chilocorus distigma

Taxon classificationAnimaliaColeopteraCoccinellidae

Klug, 1835

012F8EF9-4137-5CF2-B368-35CB3FB52D5B

Chilocorus distigma Klug, 1835: 49.

###### Remarks.

This is a rare species and found only in natural ecosystems. It was found only during February and March and was collected using a SN.

###### Material examined.

**Asir**: Al Majardah, Wadi Qanunah, 19°24.67'N, 41°36.39'E, 348 m, 11.III.2012, SN, Al Dhafer et al., 2♀4♂71exs; 11.III.2012, LT, Al Dhafer et al., 1♀7exs; Baisha, Wadi Targ, 19°37.37'N, 42°18.02'E, 1370 m, 14.III.2012, SN, Al Dhafer et al., 1ex; Muhail, Wadi Hali, 18°30.12'N, 42°02.21'E, 440 m, 11.II.2016, SN, Fadl, H., 1ex.

###### Local distribution.

This species was collected from Asir and was previously listed by Kovář (2007).

###### World distribution.

**Asia**: SA; **AFR** (Kovář 2007); YE (Raimundo and van Harten 2000).

#### *Parexochomus* Barovskij, 1922

##### Parexochomus nigripennis

Taxon classificationAnimaliaColeopteraCoccinellidae

(Erichson, 1843)

527427AE-A05D-510A-8CCC-FEC3B88021DA

Chilocorus nigripennis Erichson, 1843: 267.

###### Remarks.

This species was reported as a predator of mealybugs (Talhouk 1982) and is known as the biocontrol agent of aphids such as *Aphis nerii* Boyer de Fonscolombe, 1841, a pest of *Nerium oleander* L., and a predator on the soft scale insect *Najacoccus serpentinus* Green, 1919, a pest of *Tamarix articulata* Vahl (Martin 1972).

###### Material examined.

**Asir**: Khamis Mushait, Wadi Bisha, 18°20.01'N, 42°42.13'E, 1990 m 27.IV.2011, SN, Al Ansi, A., 1ex; **Najran**: Rijla, Wadi Najran, 17°31.56'N, 44°13.65'E, 1257 m, 15.I.2013, BS, Al Ansi et al., 4exs; Al Mofejah, 17°27.25'N, 44°03.48'E, 1263 m, 16.I.2013, BS, Al Ansi et al., 1♂1♀; **Baha**: Wadi Bawah, 20°43.93'N, 41°16.82'E, 1347 m, 8.XI.2012, BS, Fadl, H., 1ex; **Riyadh**: Dirab, 23°30'N, 46°51'E, 4.V.1987, SN, 1♀1ex; 9.XII.1990, SN, 1ex; Al Kharj, 20°24'N, 46°29"E, III.1980, SN, 1♂; Huraymila, 25°09'N, 46°08'E, 13.II.1988, SN, Al Dawood, A., 1♀2exs; Al Waseel, 24°48.40'N, 46°30.42'E, 20.X.2011, SN, Al Rashedi, H., 1ex; Ad Diriyah, 24°40'N, 46°35'E, 8.XII, SN, 1ex; 29.XII.2009, SN, Abdel-Gayyed, 1ex; **Tabouk**: Tabuk-Dhuba Rd., 28°18.39'N, 36°02.87'E, 824 m, 15.IX.2011, SN, Al Ansi, A., 1ex; **Qaseem**: Buraydah, 26°12.954'N, 44°02.48'E, 633 m, 17.IX.2011, SN, Al Ansi, A., 4exs; Riyadh, 24 43.01"N, 46 38.65"E, 1972, ANMA, 6exs; 18.IV.1978, SN, A.Talhouk et al., ANMA, 8exs, det. Fürsch, 1979; 2.II.1985, Abdulkader A., ANMA, 54 exs.

###### Local distribution.

This species was found throughout the KSA and collected from Asir, Baha, Najran, Riyadh, Tabouk, and Qaseem during this study. It was also reported by Shalaby (1962) from Asir, listed by Martin (1972), and reported by Beccari (1971) from Asir, by Fürsch (1979) from Riyadh, and by Talhouk (1982) from Riyadh and Qaseem regions.

###### World distribution.

**Asia**: AE, AF, IN, IQ, PAL, LE, PA, SA, SI, and SY; **Europe**: IT, PT, and SP; **North Africa**: AG, CI, EG, LB, MA, MO, and TU; **AFR** (Kovář 2007); YE (Raimundo and van Harten 2000).

##### Parexochomus nigromaculatus

Taxon classificationAnimaliaColeopteraCoccinellidae

(Goeze, 1777)

622EF56E-93B6-5709-AA8D-411C5F514086

Coccinella nigromaculata Goeze, 1777: 248.

###### Remarks.

This species is known as a predator of mealybugs and aphids as mentioned by R. D. Pope (1969) on the label data and was collected from wild vegetation and natural habitats. It was found from February to May and in September.

###### Material examined.

**Asir**: Abha, Sodah, 18°16.27'N, 42°21.52'E, 24.IV.2011, SN, Al Ansi, A., 1♀; Khamis Mushait, Wadi Bisha, 18°20.01'N, 42°42.13'E, 1990 m 27.IV.2011, SN, Al Ansi, A., 1♂; Abha, Habalah, 18°02.05'N, 42°51.49'E, 25.IV.2011, SN, Al Ansi, A., 1ex; Ahd Rifidh, 18°06.33'N, 42°53.82'E, 16.I.2013, SN, Al Ansi, A., 1♀; Al Majardah, Wadi Khat, 19°05.37'N, 41°58.37'E, 13.III.2012, BS, Al Dhafer et al., 1♀; 12.X.2013, SN, Sonbati, S., 1ex; Al Majardah, Wadi Qanunah, 19°24.67'N, 41°36.39'E, 348 m, 11.III.2012, SN, Al Dhafer et al., 2♀2♂; Muhail, Wadi Hali, 18°30.12'N, 42°02.21'E, 440 m, 11.II.2016, SN, Fadl, H., 2exs; El Torkey, A., SN, 1ex; **Baha**: Wadi Galah, 20°08.08'N, 41°20.56'E, 16.V.2011, SN, Fadl, H., 1♀; Al Mandaq, Wadi Turubah, 20°14.37'N, 41°15.23'E, 3.VI.2012, BS, Al Ansi, A., 1♂; 27.IX.2013, Al Dhafer, H., 2exs; Biljuraishi, 19°55.76'N, 41°26.59'E, 2053 m, 14.IV.2016, SN, Al Ansi, A., 1ex; Thee Ain, 19°55.78'N, 41°26.60'E, 741 m, 12.IV.2016, SU, Al Ansi, A., 2exs; 11.IV.2016, BS, Al Ansi et al., 2exs; Raghdan, 20°34.25'N, 41°45.11'E, 10.IV.2016, SN, Al Ansi, A., 1ex; **Makkah**: Taif, 21°08'N, 40°58'E, 10.IX.1969, R. D. Pope, ANMA, 3exs, on mealy bug.

###### Local distribution.

Adults of this species were collected from different localities of Asir, Baha, and Makkah. This species was previously listed by Fürsch (1979).

###### World distribution.

**Asia**: AF, CY, ES, FE, IN, IQ, PAL, KI, KZ, LE, PA, SY, TD, TR, UZ, WS, and XIN; **Europe**: AB, AL, AR, AU, BE, BH, BU, CR, CZ, DE, EN, FN, FI, FR, GB, GE, GG, GR, HU, IT, KI, KZ, MC, NL, NT, RO, SK, SL, SP, ST, SV, TR, UK, and YU (Kovář 2007); YE (Raimundo and van Harten 2000).

##### Parexochomus pubescens

Taxon classificationAnimaliaColeopteraCoccinellidae

(Kuster, 1848)

B535FF5E-11D2-594A-BDA1-1FB001C91FB7

Exochomus pubescens Kuster, 1848: 94.

###### Remarks.

This species is widely distributed in the kingdom and found in both natural and agroecosystems throughout the year. It was collected from *Acacia ehrenbergiana* Hayne, *A. gerrardii Benth*., *Calotropis procera* (Aiton) W. T. Aiton, *Lycium shawii* Roem. and Schult., and *Ziziphus nummularia* (Burm.f.) Wight and Arn. (Abdel-Dayem et al. 2017). It is primarily known as a predator of aphids (Kapur 1959).

###### Material examined.

**Asir**: Al Majardah, Wadi Al Talalie, 19°05.19'N, 41°47.78'E, 286 m, 1.VI.2012, BS, Al Ansi, A., 1♂; Al Majardah, Wadi Yabah, 19°16.27'N, 41°48.46'E, 411, 2.VI.2012, BS, Al Ansi, A., 1♀; 2.VI.2012, LT, Abdel-Dayem, M., 1ex; 12.III.2012, LT, 1ex; 12.III.2012, SN, Al Dhafer et al., 1ex; Al Majardah, Wadi Khat, 19°05.37'N, 41°58.37'E, 31.V.2012, BS, Al Ansi, A., 1♂; 13.III.2012, BS, Al Dhafer et al., 1ex; Al Majardah, Wadi Qanunah, 19°24.67'N, 41°36.39'E, 348 m, 11.III.2012, BS, Al Dhafer et al., 1♂; 11.III.2012, SN, Al Dhafer et al., 1ex; Abha, Sodah, 18°16.27'N, 42°21.52'E, 24.IV.2011, SN, Al Ansi et al., 1ex; Al Majardah, Thalooth Al Mandhar, Wadi Baqrah, 18°47.57'N, 42°01.12'E, 433 m, 31.V.2012, BS, Al Ansi, A., 1ex; 10.XI.2012, BS, Fadl, H., 11exs; 13.III.2012, BS, Abdel-Dayem et al., 1ex; **Baha**: Al Mandaq, Wadi Turubah, 20°14.37'N, 41°15.23'E, 3.VI.2012, LT, Al Ansi, A., 1♂2♀; 27.IX.2013, Al Dhafer, H., 2exs; Thee Ain, 19°55.78'N, 41°26.60'E, 741 m, 3.VI.2012, SN, Kondrateiff et al., 1♂4exs; 18.V.2010, BS, Sharaf, M., 1♂; 10.III.2012, BS, Al Dhafer et al., 1ex; 11.IV.2016, BS, Al Ansi et al., 1♂; Al Mikhwah, 19°49.44'N, 41°22.85'E, 430 m, 7.III.2013, BS, Al Harbi et al., 2exs; Wadi Al Zaraeib, 15.V.2010, BS, Sharaf, M., 1ex; **Jizan**: Al Aydabi, Wadi Qasi, 17°26.52'N, 42°57.32'E, 284 m, 23.V.2012, BS, Al Ansi, A., 1♂; Al Aydabi, 17°21.67'N, 43°02.70'E, 272 m, 22.V.2012, SN, Al Ansi, A., 1♂; Al Aydabi, Haqu Fifa, 17°07.95'N, 43°02.59'E, 253 m, 21.V.2012, BS, Al Ansi, A., 1♂1ex; Aiban-Sabya Rd., Wadi Shahdan, 17°28.27'N, 42°51.19'E, 433 m, 13.XI.2012, BS, Fadl, H., 1♂11exs; Abu Arish-Al Aridhah Rd., Wadi Jizan, 17°01.34'N, 42°56.73'E, 127 m, 11.XI.2012, BS, Fadl, H., 7exs; Jizan-AbuArish Rd, 17°04.25'N, 42°47.05'E, 24.II.2015, SN, Al Harbi, M., 1ex; Sabya, City Center, 17°14.27'N, 42°46.42'E, 43 m, 23.V.2012, BS, Al Ansi, A., 4exs; Ayban-Sabya Rd., Wadi Qasi, 17°26.52'N, 42°57.32'E, 284 m, 12.XI.2012, BS, Fadl, H., 2exs; Aiban, Haqu Fifa, 17°07.95'N, 43°02.59'E, 253 m, 11.XI.2012, BS, Fadl, H., 1ex; Al Aridhah, Wadi Al Rad, 17°04.10'N, 43°04.33'E, 192 m, 21.V.2012, BS, Al Ansi, A., 1ex; Hurub, Wadi Qasi, 17°26.52'N, 42°57.32'E, 284 m, 24.V.2012, BS, Al Ansi, A., 1ex; **Najran**: Wadi Shuaib Barran, 17°28.94'N, 44°05.52'E, 1325 m, 16.I.2013, BS, Al Ansi et al., 1ex; Hubuna, Lahumah, 17°50.47'N, 44°16.82'E, 1212 m, 14.I.2013, BS, Al Ansi et al., 1ex; **Riyadh**: Huraymila, 25°09'N, 46°08'E, 1.XII.1988, SN, 1ex; Shuaib Huraymila, 25°06.10'N, 46°04.22'E, 804 m, 22.IV.2012, BS, Al Ansi, A., 5exs; Rawdhet Khoraim, 25°22.98'N, 47°16.71'E, 559 m, 89exs were collected by BS and SU on branches of *A. ehrenbergiana*, *A. gerrardii*, *C. procera*, *L. shawii* and *Z. nummularia*; through IX-XII 2011; I-V, XI.2012; I, III.2013. **Qaseem**: Buraydah, 26°12.954'N, 44°02.48'E, 633 m, 17.IX.2011, SN, Al Ansi et al., 1♂1♀.

###### Local distribution.

This species was collected from a variety of habitats in Asir, Baha, Jizan, Najran, Riyadh, and Qaseem provinces and previously reported by Kapur (1959) from Hejaz; Fürsch (1979) from Riyadh, Asir, Jizan, and Makkah; and Abdel-Dayem et al. (2017) from Riyadh.

###### World distribution.

**Asia**: AF, IN, IQ, PAL, and SA; **Europe**: FR, GR, IT, and SP; **North Africa**: AG, EG, LB, MO, and TU; **AFR** (Kovář 2007); YE (Raimundo and van Harten 2000) and AE (Raimundo et al. 2007).

##### Parexochomus sjoestedti

Taxon classificationAnimaliaColeopteraCoccinellidae

Weise, 1910

530864AF-8102-515E-9538-3AFE811D86B4

Parexochomus sjoestedti Weise, 1910: 260.

###### Remarks.

This is a rare species and Afrotropical in distribution. There is no literature to confirm its presence in Saudi Arabia, except the Catalogue of Kovář (2007), and it was also not collected during this study.

###### World distribution.

**A**: SA; **AFR** (Kovář 2007).

#### Tetrabrachini Kapur, 1948

***Tetrabrachys* Kapur, 1948**

All the three species are restricted to the Arabian Peninsula and some Afrotropical countries. Although several researchers (Kapur 1948; Fürsch 1979; Kovář 2007) have reported these three species from the KSA, not a single species was recorded during the present study.

##### Tetrabrachys arabicus

Taxon classificationAnimaliaColeopteraCoccinellidae

Kapur, 1948

EA826C55-AFB7-561C-9F9F-A46B2C00FBB8

Tetrabrachys arabicus Kapur, 1948: 329.

###### Remarks.

This species is restricted to the Arabian Peninsula. It was recorded from the KSA by Kapur (1948), Fürsch (1979), and Kovář (2007) but not found during the present study.

###### Local distribution.

Al Hejaz (Kapur 1948); Asir (Fürsch 1979).

###### World distribution.

**Asia**: SA and YE (Kovář 2007).

##### Tetrabrachys minutus

Taxon classificationAnimaliaColeopteraCoccinellidae

(Pic, 1903)

22AF0567-F140-55C3-BBB0-DBE221D1F804

Lithophilus minutus Pic, 1903: 170.

###### Remarks.

This is also a rare species that was recorded from the KSA by Kapur (1948) and Kovář (2007) without definite localities. It was not found in the present study.

###### World distribution.

**Asia**: SA; **North Africa**: TU (Kovář 2007).

##### Tetrabrachys tenebrosus

Taxon classificationAnimaliaColeopteraCoccinellidae

(Weise, 1910)

4428CE16-9FAA-5836-97E5-2B3491241630

Lithophilus tenebrosus Weise, 1910: 53.

###### Remarks.

This is a rare species found in the Arabian Peninsula and Africa; it was recorded from the KSA by Kapur (1948) and from the KSA and Yemen by Kovář (2007) without definite localities. It was not found in the present study.

###### World distribution.

**Asia**: SA and YE; **AFR** (Kovář 2007).

#### Coccinellini Latreille, 1807

***Bulaea* Mulsant, 1850**

##### Bulaea lividula bocandei

Taxon classificationAnimaliaColeopteraCoccinellidae

Mulsant, 1850

F8402CCB-1495-5F7E-BABC-BE66E72CCFDF

Bulaea bocandei Mulsant, 1850: 71, 1016.

###### Remarks.

Members of *Bulaea* are phytophagous and feed on the pollen of plants. This species was collected on *Aerva javanica* (Burm. f.) Juss. ex Schult (Amaranthaceae), and it was also known to feed on leaves and pollen (Raimundo et al. 2007).

###### Material examined.

**Asir**: Al Majardah, Wadi Khat, 19°05.37'N, 41°58.37'E, 31.V.2012, BS, Al Ansi, A., 1♀12exs; Wadi Qanunah, 19°24.67'N, 41°36.39'E, 348 m, 11.V.2011, SN, Fadl, H.; Setyaningrum et al., 1ex; Wadi Yabah, 19°16.27'N, 41°48.46'E, 411, 15.IV.2016, SN, Soliman, A., 2exs; Maraha, Wadi Reem, 17°52.55'N, 42°16.66'E, 136 m, 9.II.2016, SN, Fadl, H., 2exs; Maraha, Wadi Ramlan, 17°47.18'N, 42°22.95'E, 180 m, 9.II.2016, SN, Al Ansi, A., 3exs; SU, Al Ansi, A., 12exs; **Baha**: Thee Ain, 19°55.78'N, 41°26.60'E, 741 m, 14.IV.2016, SN, Soliman, A., 1ex; **Jizan**: Abu Arish Wadi Jizan, 17°01.34'N, 42°56.73'E, 127 m, 21.V.2012, BS, Al Ansi, A., 577exs; 21.V.2012, BS, Al Ansi, A., 4♂2♀; Hurub-Al Sahaleel Rd., 17°47.34'N, 42°85.65'E, 456 m, 24.V.2012, BS, Al Ansi, A., 7exs; Al Aydabi Jaorat Aiban, 17°25.53'N, 43°03.50'E, 343 m, 23.V.2012, SN, Al Ansi, A., 2exs; 11.XI.2012, BS, Fadl, H., 21exs; Al Aydabi Haqu Fifa, 17°21.54'N, 43°02.62'E, 237 m, 21.V.2012, SN, Al Ansi, A., 1ex; Aiban Sabya Rd. Wadi Shahadan, 13.XI.2012, BS, Fadl, H., 6exs; Jizan-Addarab Wadi Baiz, 17°37.56'N, 42°22.24'E, 249 m, 24.II.2015, HP, Al Harbi, M., 6exs; Jizan, 10.II.1982, SN, Talhouk et al., ANMA, 22exs; 25.XII.1980, SN, Talhouk et al., ANMA, 3exs; 15.VIII.1982, SN, Talhouk et al., ANMA, 1ex. **Riyadh**: Al Uyaynah, 24°53'N, 46°22'E, 24.V.2007, SN, 1ex; Rawdhet Khoraim, 25°22.98'N, 47°16.71'E, 559 m, 29.VIII.2013, BS, 1ex, on *Ziziphus nummularia*; Wadi Ad Dawasir, 20°25.83'N, 44°56.53'E, 641 m, 17.I.2013, BS, Al Ansi et al., 10exs.

###### Local distribution.

This species was found in Asir, Baha, Jizan, and Riyadh, but the majority of specimens of *B. lividula bocandei* were collected from several localities of Jizan province. This species was previously reported by Kapur (1959) from Asir, by Fürsch (1979) from the Eastern region and by Abdel-Dayem et al. (2017) from Riyadh.

###### World distribution.

**Asia**: AE, IN, IQ, PAL, JO, PA, SA, SY, and YE; **North Africa**: AG, EG, and MO; **AFR** (Kovář 2007).

#### *Cheilomenes* Chevrolat, 1836

##### Cheilomenes lunata lunata

Taxon classificationAnimaliaColeopteraCoccinellidae

(Fabricius, 1775) *

4BB164A5-23E1-559F-B75C-4702AE9EB08D

Figure 2

Coccinella lunata lunata Fabricius, 1775: 86.

###### Diagnosis.

Pronotum is black with white anterolateral angles and anterior margin; elytra are black with large and rounded yellow-reddish spots. Pattern of spots on each elytron: separated spots on the shoulder, but mostly shoulder spot united with scutellar spot to a semi-circular spot outside the humeral callus; two rounded spots in the elytral centre: one near the suture and one in a more marginal position; two spots on the posterior half of elytron: one forming an inverted horizontal C and the other one stretching along the posterolateral elytral margin.

**Figure 2.**
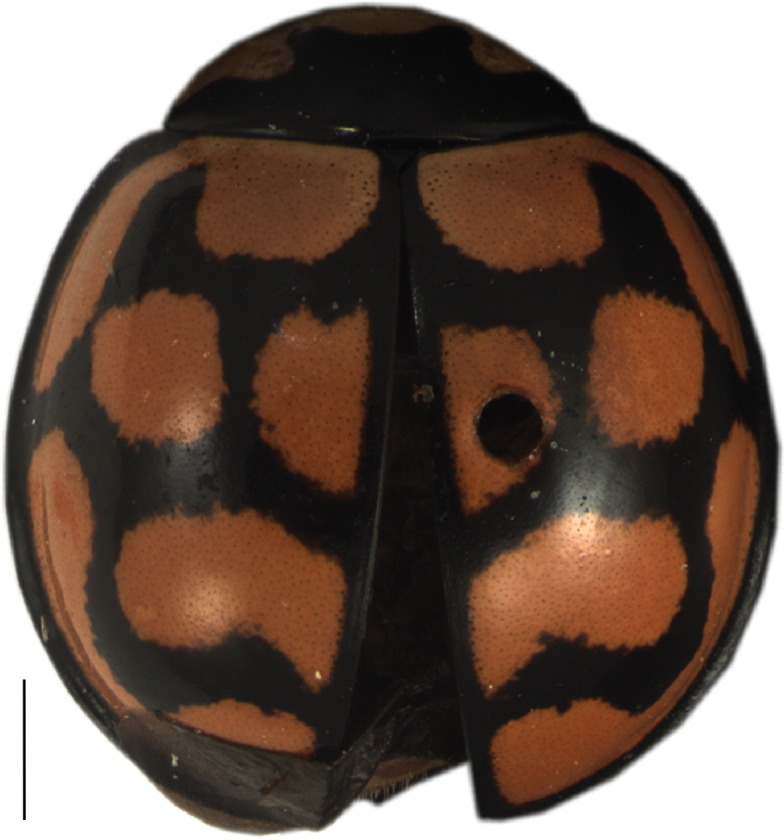
Dorsal view of *Cheilomenes lunata lunata*. Scale bar: 1 mm.

###### Material examined.

**Asir**: Abha, Raydah, 18°12.10'N, 42°24.54'E, 2578 m, 16.IV.2016, SN, Soliman, A., 1♀; Rijal Alma, Wadi Sabin, 17°48.25'N, 42°21.64'E, 194 m, 10.II.2016, SU, Al Ansi, A., 1♂1♀; **Baha**: Wadi Turubah, 20°14.37'N, 41°15.23'E, 10.V.2011, SN, Fadl et al., 1♂.

###### Local distribution.

This species was previously recorded from Yemen by Raimundo et al. (2006) and collected from Asir and Baha provinces during the present study.

###### World distribution.

**North Africa**: EG; **AFR** (Kovář 2007); **Asia**: YE (Raimundo et al. 2006) and a new country record for the KSA.

##### Cheilomenes lunata yemenensis

Taxon classificationAnimaliaColeopteraCoccinellidae

Fürsch, 1989

B1C5C6F6-4E1F-537F-81D3-5C353342B247

Cheilomenes lunata yemenensis Fürsch, 1989 (2): 37–38.

###### Remark.

A rare subspecies that is confined only to the Arabian Peninsula and was described from Yemen. In Saudi Arabia, it is widely distributed in both natural habitats and agroecosystems.

###### Materials examined.

**Asir**: Abha, Sodah, 18°16.27'N, 42°21.52'E, 24.IV.2011, SN, Al Ansi et al., 1ex; Al Majardah, Wadi Yabah, 19°16.27'N, 41°48.46'E, 411, 2.VI.2012, BS, Al Ansi, A., 1♂; 12.III.2012, SN, Al Dhafer et al., 1ex; Wadi Turubah, 20°14.37'N, 41°15.23'E, 14.V.2011, SN, Fadl et al., 1♂; 14.X.2010, SN, Al Dhafer et al., 1♀; Abha, Raydah, 18°12.10'N, 42°24.54'E, 2578 m, 8.II.2016, SU, Al Ansi, A., 1ex; 16.IV.2016, SN, Soliman, A., 2exs; Al Ansi et al., SU, 1ex; Rijal Alma, Wadi Sabin, 17°48.25'N, 42°21.64'E, 194 m, 10.II.2016, SN, Fadl, H., 1ex; **Baha**: Wadi Dhahyan Dam, 20°05.51'N, 41°24.11'E, 883 m, 9.V.2011, SN, Fadl et al., 1ex; Ghabat Amdhan, 20°06.32'N, 41°17.15'E, 19.V.2010, SN, El Torkey, A., 1ex; Shada Al Aala, 19°50.58'N, 41°18.69'E, 1666 m,14.IV.2016, HP, Abdel-Dayem, M., 1ex; Thee Ain, 19°55.78'N, 41°26.60'E, 741 m, 12.IV.2016, SN, Al Ansi, A., 1ex; Raghdan, 20°34.25'N, 41°45.11'E, 10.IV.2016, SU, Al Ansi, A., 1ex; **Jizan**: Fifa, 17°16'N, 43°05'E, 1.II.1983, SN, Talhouk et al., ANMA, 15exs; 25.I.1983, SN, ANMA, 38exs; **Makkah**: Taif Rd., 12.X.2010, SN, Al Dhafer et al., 2exs.

###### Local distribution.

Specimens of this species were collected from Asir, Baha, Jizan, and Makkah provinces. It was previously reported from the KSA by Kovář (2007) but with no distributional range.

###### World distribution.

**Asia**: This species is confined to the Arabian Peninsula and was recorded only from SA and YE (Kovář 2007).

##### Cheilomenes propinqua vicina

Taxon classificationAnimaliaColeopteraCoccinellidae

(Mulsant, 1850)

84723CFA-500A-5687-9390-BCA2294833B6

Cydonia propinqua vicina Mulsant, 1850: 411.

###### Remark.

This species is associated with a wide range of both natural habitats and agroecosystems, predating on other arthropods. It was known to predate particularly on aphids such as *Rhopalosiphum maidis* (Fitch, 1856), a pest of *Setaria italica* (L.) (Martin 1972; Raimundo and van Harten 2000).

###### Materials examined.

**Asir**: Khamis Mushait, Wadi Bisha, 18°20.01'N, 42°42.13'E, 1990 m 27.IV.2011, SN, Al Ansi, A., 1ex; Al Majardah Wadi Yabah, 19°16.27'N, 41°48.46'E, 411, 12.V.2012, Al Ansi, A., LT 2exs, SN 5exs; Al Majardah, Wadi Al Talalie, 19°05.19'N, 41°47.78'E, 286 m, 12.III.2012, SN, Al Ansi, A., 2exs; Al Majardah, Wadi Khat, 19°05.37'N, 41°58.37'E, 13.III.2012, BS, Al Dhafer et al., 2exs; Khamis Mushait, 18°18'N, 42°45'E, 4.X.2005, SN, 1ex; Wadi Abha, 18°22.03'N, 42°50.82'E, 1990 m, 28.IV.2011, SN, Al Ansi, A., 2exs; Habalah, 18°02.05'N, 42°51.49'E, 25.IV.2011, SN, Al Ansi, A., 1ex; Al Majardah Wadi Qanunah, 19°24.67'N, 41°36.39'E, 348 m, 11.III.2012, SN, Al Dhafer et al., 4exs; 11.V.2011, SN, Fadl, H., 1♂6exs; Ahd Rifidh, 18°06.33'N, 42°53.82'E, 16.I.2013, SN, Al Ansi et al., 1ex; Muhail, Wadi Hali, 18°30.12'N, 42°02.21'E, 440 m, 11.II.2016, SU, Al Ansi, A., 4exs; Rijal Alma, Wadi Sabin, 17°48.25'N, 42°21.64'E, 194 m, 10.II.2016, SN, Fadl, H., 1ex; Maraha, Wadi Reem, 17°52.55'N, 42°16.66'E, 136 m, 9.II.2016, SU, Al Ansi, A., 5exs; Maraha, Wadi Ramlan, 17°47.18'N, 42°22.95'E, 180 m, 10.II.2016, SU, Al Ansi et al., 2exs; Maraha, Wadi Itwad, 17°48.25'N, 42°21.64'E, 149 m, 8.II.2016, SU, Al Ansi, A., 2exs; **Baha**: Wadi Turubah, 20°14.37'N, 41°15.23'E, 14.V.2011, SN, Fadl, H., 2exs; 14.III.2012, SN, Fadl, H., 1ex; Thee Ain, 19°55.78'N, 41°26.60'E, 741 m, 11.V.2011, SN, Fadl, H., 8exs; 14.IV.2016, SN, Soliman, A., 1ex; 12.IV.2016, SU, Al Ansi, A., 3exs; SN, 1ex; 15.V.2011, SN, Fadl, H., 2♂8exs; 3.VI.2012, HP, Abdel-Dayem, M., 1ex; Wadi Milan, 19°50.79'N, 41°21.01'E, 481 m, 7.III.2013, SN, Al Harbi et al., 2exs; Al Mikhwah-Muhail Rd. Hadba Hamra, 19°13.19'N, 41°46.67'E, 374 m,15.IV.2016, SN, Al Ansi, A., 1ex; Bani Hassan, 20°20.99'N, 41°21.85'E, 2214 m,12.IV.2016, SN, Soliman, A., 1ex; **Jizan**: Jizan-Ahd Al Masareha Rd., 17°02.28'N, 42°52.38'E, 12.III.2010, SN, Al Dhafer et al., 1ex; 11.III.2010, LT, Al Dhafer et al., 1♂; Hurub-Al Sahaleel Rd., 17°47.34'N, 42°85.65'E, 456 m, 24.V.2012, BS, Al Ansi, A., 2exs; Jizan city, 16°54'N, 42°29'E, 10.II.1982, SN, Talhouk et al., ANMA, 34exs; same data except 10.II.1981, SN, ANMA, 11exs; same data except 1.II.1983, SN, ANMA, 1ex; same data except 20.I.1979, SN, ANMA, 3exs; same data except 16.VIII.1977, SN, Fathy S., ANMA, 1ex.; **Makkah**: Jedah-Makkah Rd., 21°25.14'N, 39°19.49'E, 15.XII.2006, SN, 2exs; Baha-Taif Rd. Bani Malik, Wadi Rakhmat, 20°42.15'N, 40°59.46'E, 1616 m, 20°42.15'N, 40°59.46'E, 1616 m, 4.VI.2012, SN, Al Ansi, A., 1♂2exs; **Najran**: Rijla Wadi Najran, 17°31.56'N, 44°13.65'E, 1257 m, 15.I.2013, BS, Al Ansi et al., 3exs; Wadi Shuaib Barran, 17°28.94'N, 44°05.52'E, 1325 m, 16.I.2013, BS, Al Ansi et al., 3♂2exs; **Riyadh**: Riyadh, SN, Abu Yaman, 1ex; Education Farm, 24°40'N, 46°35'E, 26.III.1975, SN, 1ex; Ad Diriyah, 24°40'N, 46°35'E, 15.XII.2005, SN, 1ex; Dirab 23°30'N, 46°51'E, 13.III.2003, SN, 1ex.

###### Local distribution.

Specimens of this species were collected from Asir, Baha, Jizan, Makkah, Najran, and Riyadh and previously reported by Kapur (1959) from Asir and Al Hajaz, by Beccari (1971) from Jizan and Madinah, by Fürsch (1979) from Asir, and by Talhouk (1982) from Jizan, and listed by Martin (1972).

###### World distribution.

**Asia**: IQ, PAL, LE, SA, and YE; **North Africa**: AG, EG, and LB; **AFR** (Kovář 2007).

#### *Coccinella* L., 1758

##### Coccinella septempunctata

Taxon classificationAnimaliaColeopteraCoccinellidae

L., 1758

301DD973-6B70-5F6C-92E3-9A6FC281E528

Coccinella septempunctata L., 1758: 365.

###### Remark.

This species is widely distributed around the world and found in a wide range of habitats. Both adults and larvae are voracious predators of aphids (Sattar et al. 2008). It is a biocontrol agent of *Aphis craccivora* C.L. Koch, 1854, and *Therioaphis trifolii* (Monell, 1882), pests of alfalfa, *Medicago sativa* L. (Martin 1972).

###### Material examined.

**Eastern Province**: Al Ahsa, 22°17.53'N, 50°40.46'E, 4.III.2011, SN, Al Ansi, A. 1ex, on alfalfa; 4.III.2011, SN, Al Ansi, A., 1ex; **Riyadh**: Al Ammariah, 24°49'N, 46°26'E, 28.I.2009, SN, Widman, A., 1ex, on alfalfa; 28.I.2008, SN, Valenza, B., 2exs, on alfalfa; 28.I.2009, SN, Setyaningrum, H., 1♀1ex; Ad Diriyah, 24°40'N, 46°35'E, 29.X.2008, SN, Al Ahmari, A., 1♀, on alfalfa; 29.X.2008, SN, Bel Hareth, H., 2exs, on alfalfa; 16.I.2009, SN, Setyaningrum, H., 1ex; 29.X.2008, SN, Al Sebaiei, M., 4exs, on alfalfa; 29.X.2008, SN, Al Qahtani, M., 1ex, on alfalfa; 17.IV.2003, SN, Al Habeeb, A., 1ex, on alfalfa; 17.X.2003, SN, Al Saiari, M., 1ex; 30.XII.1989, SN, Amro, 1ex, on pepper; 21.II, SN, Al Essa, M., 1ex; Al Kharj, 20°24'N, 46°29'E, 24.III.2010, SN, Al Othman, A., 1ex; 26.XI.2008, SN, Sofan, A., 3exs, on water melon; 26.XI.2008, SN, Widman, A., 1♂, on alfalfa; 14.XI.2007, SN, 1ex; 26.X.1991, SN, Al Ahmadi, F., 1ex, on alfalfa; 9.III.2001, SN, Al Baqami, A., 1ex; Al 16.X, SN, Al Sharaf, M., 1ex; Al Uyaynah, 24°53'N, 46°22'E, 10.III.2010, SN, Al Hashel, A., 1ex; 30.XI.2010, SN, Al Ansi, A., 1ex, on corn; 11.XI.2008, SN, Setyaningrum, H., 3exs; 11.XI.2008, SN, Setyaningrum, H., 1ex; 26.II.2004, SN, Droshy, R., 1ex, on alfalfa; 7.III.2004, SN, Jasim, D., 1♂; 4.V.2005, SN, Turkestani, S., 1ex; 2.X.2003, SN, Al Haj, M., 1ex; 9.IV.2008, SN, Al Tork, 1ex, on alfalfa; Al Waseel, 24°48.40'N, 46°30.42'E, 12.XI.2008, SN, Al Qahtani, M., 1ex, on alfalfa; 1.XII.2010, SN, Al Abdul Muhsen, M., 1ex; 12.XI.2008, SN, Namoosy, S., 2exs, on alfalfa; 20.XI.2011, SN, Al Mater, A., 1ex; 11.X, SN, Al Farhan, 1ex, on alfalfa; 20.XI.2011, SN, Al Sahwan, K., 1ex; 12.XI.2008, SN, Baziyad, A., 3exs, on alfalfa; Riyadh, SN, Abu Yaman, 1ex; Dirab, 23°30'N, 46°51'E, 30.XII.2009, SN, Setyaningrum, H., 1ex, on grass; 7.III.2011, SN, Salah, A., 1ex, on fig plant; 25.I.1990, SN, 1ex, on alfalfa; 10.X.2011, SN, Al Amro, S., 1ex; Hautet Bani Tamim, 23°27.26'N, 46°41.13'E, 4.IV.2008, SN, Al Dhafer et al., 2exs; Otaiqah, 24°35.46'N, 46°42.24'E, 16.XI.1989, SN, 1ex, on Squash; Rawdhet Khoraim, 25°22.98'N, 47°16.71'E, 559 m, 11.IV.2011, SN, 1ex; Al Uayainnah, 25.II.2015, SN, Al Sofayan, M., 1ex; Riyadh, 15.III.1978, SN, Talhouk et al., ANMA, 5exs;**Tabuk**: Tabuk-Dhuba Rd., 28°18.39'N, 36°02.87'E, 824 m, 15.IX.2011, SN, Al Ansi, A., 1ex; Tabuk-Madinah Rd., 28°23.47'N, 36°51.96'E, 808 m, 14.IX.2011, SN, Al Ansi, A., 1ex.

###### Local distribution.

Its specimens were collected from Eastern Province, Riyadh and Tabouk; this species was previously listed by Martin (1972), Talhouk (1982), and Walker and Pittaway (1987) and reported by Beccari (1971) from Riyadh, by Fürsch (1979) from Riyadh, and by Abdel-Dayem et al. (2017) from Riyadh.

###### World distribution.

**Asia**: AF, ANH, BEI, BT, CY, FE, FUG, GAN, GUA, GUI, GUX, HEB, HEI, HEN, HUB, HUN, IN, IQ, PAL, JA, JIA, JIL, JIX, JO, KA, KI, KU, KZ, LE, LIA, NIN, NMO, MG, NC, NP, PA, QIN, SA, SCH, SC, SD, SHA, SHG, SHN, SHX, SI, SY, TAI, TD, TIA, TM, TR, UP, UZ, XIN, XIZ, YUN, and ZHE; **Europe**: AB, AL, AN, AR, AU, BE, BH, BU, BY, CR, CT, CZ, DE, EN, FI, FR, GB, GE, GG, GR, HU, IR, IT, LA, LS, LT, LU, MC, MD, NL, NR, NT, PL, RO, SK, SL, SP, ST, SV, SZ, TR, UK, and YU; **North Africa**: MR; AFR, NAR, and ORR (Kovář 2007).

##### Coccinella undecimpunctata menetriesi

Taxon classificationAnimaliaColeopteraCoccinellidae

Mulsant, 1850

52406F8E-5A15-522D-90D6-D805759E6399

Coccinella undecimpunctata menetriesi Mulsant, 1850: 104.

###### Remark.

This species with several subspecies is widely distributed throughout the world. Both adults and larvae are known as predators of aphids. It is commonly found in the KSA throughout the year. It is known as a good biocontrol agent of *Aphis craccivora* C.L. Koch, 1854, and *Therioaphis trifolii* (Monell, 1882), pests of alfalfa, *Medicago sativa* L. (Martin 1972).

###### Material examined.

**Asir**: Wadi Abha, 18°22.03'N, 42°50.82'E, 1990 m, 28.IV.2011, SN, Al Ansi et al., 1ex; Al Majardah, Wadi Yabah, 19°16.27'N, 41°48.46'E, 411, 2.VI.2012, SN, Al Ansi, A.M 1ex; 12.III.2012, SN, Al Dhafer et al., 1ex; Wadi Targ, 19°37.37'N, 42°18.02'E, 1370 m, 14.III.2012, HP, Abdel-Dayem, M., 1ex; **Baha**: Wadi Bawah, 20°43.93'N, 41°16.82'E, 1347 m, 20°44.98'N, 41°14.85'E, 1310 m, 8.XI.2012, SN, Fadl, H., 1ex; 8.XI.2012, SN, Abdel-Dayem, M., 1ex; Wadi Turubah, 20°14.37'N, 41°15.23'E, 14X.2010, SN, Al Dhafer et al., 1ex; Taif, 21°08'N, 40°58'E, 20.I.1993, SN, Al Saad, B., 1ex; Baha-Taif Rd. Bani Malik Wadi Rakhmat, 20°42.15'N, 40°59.46'E, 1616 m, 20°42.15'N, 40°59.46'E, 1616 m, 4.VI.2012, SN, Al Ansi, A., 1ex; **Eastern Province**: Abu Hadriyah-Dammam Rd. North Rawabi Farm, 27°34.21'N, 48°50.53'E, 15 m, 3.III.2011, SN, 1ex; Hafuf, 25°15'N, 49°32'E, 19.II.1980, SN, 2exs; Dammam-Riyadh Rd., 250 km before Riyadh, 25°50.00'N, 49°50.23'E, 174 m, 25°50.00'N, 49°50.23'E, 174 m, 5.III.2011, SN, 21exs; An Nuayriyah, 27°25.26'N, 48°27.20'E, 67 m, 2.III.2011, SN, 3exs; 2.III.2011, SN, 20exs; Abu Hadriyah-Dammam Rd., 27°34.21'N, 48°50.53'E, 15 m, 2.III.2011, SN, 3exs; Al Ahsa, 22°17.53'N, 50°40.46'E, 4.III.2011, SN, 5exs, on alfalfa; 4.III.2011, SN, 6exs, on weeds; 4.III.2011, SN, 14exs, on weeds; 4.III.2011, SN, 3exs, on weeds; Qatif, 26°45'N, 49°58'E, 20.VIII, SN, Al Salem, S., 1ex; same V.1966, ANMA, 1ex, predator on *Parlatoria blanchardii*; 4.III.1979, SN, Sarhan et al., ANMA, 29exs; 25.IX.1978, SN, Talhouk et al., ANMA, 13exs; 15.IX.1978, SN, Shalbay, F., ANMA, 1ex; SN, Buttikeri, W., ANMA, 1ex; **Najran**: As Sullayil-Najran Rd., 340 km before Najran, 18°01.35'N, 42°45.81'E, 619 m, 13.I.2013, SN, Al Ansi et al., 1ex; **Riyadh**: Rawdat Khoraim, 25°25.94'N, 47°13.86'E, 520 m, collected through the year 2011–2013 using SN, BS, PT, SU and LT on *Rhazya stricta*, *L. shawii*, *Ziziphus nummularia*, and *A. gerrardii*, Al Dhafer et al., 66exs; Wadi Namar, 24°32.18'N, 46°34.60'E, 6.IV.2012, SN, Al Othman, A., 1ex; 29.II.2012, SN, Al Ansi, A., 5exs; Otaiygah, 16.XI.1989, SN, 6exs, on alfalfa; Al-Quway’iyah, 24°20'N, 45°09'E, 18.V.2012, SN, Al Ghamdi, R., 1ex; Al Muzahimiyah, Al Khararah, 24°24.21'N, 46°14.40'E, 17.IV.2012, SN, Al Dhafer et al., 4exs; 3.IV.2012, SN, Al Dhafer et al., 8exs; 21.II.2012, SN, Al Dhafer et al., 2exs; 17.IV.2012, LT, Al Dhafer et al., 1♂1♀2exs; 17.IV.2012, SN, Abdel-Dayem, M., 1ex; 3.X.2011, SN, Al Dhafer et al., 1ex; 25.VI, Jubari, 2exs; 26.IV.2011, SN, Al Deryhim et al., 1ex; Shuaib Huraymila, 25°06.10'N, 46°04.22'E, 804 m, 22.IV.2012, SN, Rasool, I., 1ex; Huraymila, 25°09'N, 46°08'E, 12.X.2000, SN, Al Osimi, 1ex; 13.II.1988, SN, Dawood, 1ex, on weeds; Ilaisha, 24°37.58'N, 46°41.14'E, 18.XII.1989, SN, 1ex, on cowpea; Sajir, 24°51.23'N, 45°42.39'E, 17.XI.2011, SN, Setyaningrum, H., 6exs; Al Kharj, 20°24'N, 46°29'E, 18.XI.2009, SN, Al Dhafer et al., 5exs, on *Calotropis*; 24.III.2010, SN, Al Mutairi, T., 3exs; 24.III.2010, SN, Al Hori, M., 1ex; 20.XI.2009, SN, Sofan, A., 1ex; 26.XI.2008, SN, Namosi, S., 1ex; 2.V.2007, SN, 2exs;14.XI.2007, SN, Ahmed, 2exs; 8.XI.1986, SN, 1ex, on alfalfa; 26.XI.2008, SN, Valenza, B., 1ex; 26.X.1996, SN, Al Ahmed, F., 3exs on alfalfa; 17.IV.2003, SN, Al Habeeb, A., 2exs, on alfalfa; 9.III.2011, SN, Al Hazmi, H., 1ex, on weeds; 26.X.2008, SN, Setyaningrum, H., 3exs, on vegetables; 1.IV.2009, SN, Al Zahrani, M., 2exs; 1.IV.2009, SN, Al Amri, A., 2exs; 2.I.2008, SN, Al Bohmi, 1ex; 24.III.2010, SN, Al Dawsari, M., 1ex; 16.X, SN, Mostafa, 1ex; 26.X.1991, SN, Al Ahmed, F., 2exs, on Alfalfa; III.1980, SN, 1ex, on wheat; Al Kharj Al Rafei, 20°24'N, 46°29'E, 16.X.2003, SN, Al Hamad, M., 1ex; 29.XII.2002, SN, Saif, A., 1ex, on sorghum; 4.IV.2007, SN, Al Shehri, 1ex; 4.IV.2007, SN, Al Hamzi, A., 1ex; 17.V.2007, SN, Al Greed, S., 1ex; Al Haair, 24°27'N, 46°51'E, 31.I.1989, SN, Salem, 14exs, on barley; 25.IX.1990, SN, Salem, 1ex, on weeds; 14.V.1989, SN, Salem, 1ex on weeds; 13.XI, SN, Al Mutlaq, 1ex, on eggplant; 13.XI.1990, SN, Al Qarni, 1ex; 13.XI, SN, Al Jaberi, 1ex, on vegetables; 1.II.2009, SN, Setyaningrum, H., 1ex, on alfalfa; 2.XII.1988, SN, Al Fuhaid, M., 1ex; Wadi Hanifah, 24°45'N, 46°36'E, 26.I.1977, SN, Al Shaqaty, M., 1ex; Al Ammariah, 24°49'N, 46°26'E, 13.III.2013, SN, Al Qahtani, F., 1ex; 4.X.2012, SN, Hussain, S., 3exs; 4.X.2012, SN, Rasool, I., 2exs; 22.II.2012, SN, Al Atwi, M., 1ex; 23.I, SN, Al Awadhi, A., 4exs; 8.IV.2007, SN, Bin Jaber, A., 1ex; 11.X, SN, 1ex, on Weeds; 15.XII.2011, SN, Al Omar, A., 1ex; 18.XI.2009, SN, Sofan, A., 1ex; 18.XI.2009, SN, Setyaningrum, H., 2exs; 28.I.2009, SN, Arya, 2exs; 28.I.2009, SN, Setyaningrum, H., 1ex; 21.X.2010, SN, Koko, 4exs; 30.XII.2008, SN, Abdel-Gayed, 2exs; 11.XI.2009, SN, Al Ansi, A., 2exs; 1.I.2010, SN, Koko, 1ex; 1.I.2010, SN, Single, A., 1ex; 22.V.2011, SN, Al Ahmari, S., 2exs; Hawtet Bani Tammem, 23°28.10'N, 46°36.35'E, 7.V.2012, SN, Al Ansi, A., 15exs; 7.V.2012, SN, Al Dhafer et al., 2exs; 10.XI.2011, SN, Al Abdullah, I., 1ex; 21.III.2008, SN, Al Dhafer et al., M., 2exs; 4.IV.2008, SN, Al Dhafer et al., 9exs; Ad Diriyah, Education Farm, 24°40'N, 46°35'E, 19.XII.2002, SN, 1ex; 25.XII.2010, SN, Al Otaibi, F., 1ex; Ad Diriyah, 24°40'N, 46°35'E, 20.III.2009, Husain, M., 1ex; 20.IV.1992, SN, Al Asmari, D., 1ex; 11.X.2010, SN, Abdel-Gayed, 1ex; 30.XII.1989, SN, Amro, 4exs, on vegetables; 21.X.1993, SN, Hatem, 1ex; 5.V, SN, Aseri, M., 1ex; 19.III.1989, SN, 1ex, on alfalfa; 2.III.1989, SN, 1ex, on grasses; 29.III.1989, SN, 2exs, on grasses; 24.III, SN, 5exs, on vegetables; 8.X.2009, SN, Single, A., 1ex; 22.XII.2002, SN, 1ex; 9.XII, SN, 1ex, on alfalfa; 7.XI.1990, SN, Al Saleh, 1ex, on vegetables; 16.X.1989, SN, 5exs, on weeds; 23.X.1989, SN, 1, on alfalfa; 7.XI, SN, Al Qarni, 1ex, on vegetables; 5.XII.2010, SN, Al Turkey, I., 1ex; 2.VII.2009, SN, Al Zahrani, M., 1ex; 24.XII.1991, SN, Ghazi, 2exs; 7.III.2012, SN, Basahih, J., 1ex; 4.VII, SN, Al Qathban, M., 1ex, on alfalfa; 5.X.2004, SN, Al Otaibi, B., 1ex, on alfalfa; 8.XII, SN, Al Saab, 1ex, on alfalfa; 2.XII.1988, SN, Al Fuhaid, M., 1ex, feed on aphid; 16.X.2003, SN, Al Dhubaib, N., 2exs; 4.V.2005, SN, Turkestani, S., 1ex; III.1980, 1ex, on date palm, det. T. G. Vazirani, 1983; 15.XII.1973, SN, Atiyat Allah, A., 1ex; 29.X.2008, SN, Al Ahmari, A., 1ex; 30.XI.2011, SN, Al Dehaaesh, N., 1ex; XI.1989, SN, 2exs, on alfalfa; 16.III.1989, SN, Salem, 1ex, on alfalfa; 7.VII.1991, SN, Al Fudhail, M. 1ex, on alfalfa; 6.X.2003, SN, Al Hammad, M., 1ex; 12.IV.1987, SN, 1ex, on alfalfa; 5.VI.1993, SN, Aziz, 1ex; 21.I, SN, Al Harbi, S., 2exs, on alfalfa; 30.X.1989, SN, 2exs, on alfalfa; 29.X.2008, SN, Al Qahtani, M., 3exs, on alfalfa; 16.X.2000, SN, Al Khurigi, 3exs; 7.XI.1990, SN, Al Mutlaq, 1ex, on vegetables; 20.IV.1992, SN, Saif, A., 1ex, on alfalfa; 15.IV.2000, SN, Hatan, 1ex; 28.IX.2003, SN, Al Otaibi, A., 2ex; 16.I, SN, Moshal, 1ex, on alfalfa; 14.IV.1993, SN, Aziz, 1ex; 12.IV.1993, SN, Aziz, 1ex; 15.IV.1993, SN, 1ex, on crops; 19.VIII, SN, Aiman, 2ex; 8.II.2001, SN, Al Qarni, M., 1ex; 23.XI.2011, SN, Al Kassem, A., 1ex; 24.III, 1ex, on vegetables; 16.X.1989, SN, 1ex, on grasses; 13.XI.1989, SN, 1ex, on wheat; 29.III.1989, 1ex, on weeds; XII.1980, SN, 1ex, on alfalfa; Irqah, 8.XII.2010, SN, Al Dhahbani, I., 1ex; Dirab, 23°30'N, 46°51'E, 5.V.1987, SN, 1ex, on alfalfa; 24.II.1986, LT, 1ex; 1.V.1997, 1ex; 25.XI.1990, Amr, 1ex, on alfalfa,; 17.XI., Al Bathi, F., 1ex, on alfalfa; 9.I, 2exs, on alfalfa; 16.V.2007, SN, Sharaf Addeen, A., 2exs; 16.III.2005, SN, 1ex;18.III.2004, SN, Yaser, 1ex; 18.IV.1982, 1ex, on sorghum; 13.III.2003, Al Habeeb, A., 1ex, on alfalfa; 2.I.1987, SN, 1ex; 4.VIII, SN, Al Osimi, 1ex; 24.III.1987, 1ex, on alfalfa; 5.I.1987, 1ex, on alfalfa; 19.V.2009, SN, Al Moteb, M., 1ex; IX.1989, 2exs, on alfalfa; 7.IV, 5exs, on alfalfa; 14.IV.1987, 1ex, on alfalfa; 9.V.1991, SN, Al Fudhail, M., 1ex; 3.III.1987, Amro, 3exs, on alfalfa; 31.III.1987, 3exs, on alfalfa; 17.X.2011, SN, Al Khudhairi, H., 3exs; 10.X.2011, SN, Al Amro, S., 2exs; 30.XII.2009, Setyaningrum, H., 3exs, on grasses; 7.IV.1987, 4exs, on alfalfa; 17.X.2011, SN, Jubran, R., 1ex; 21.IV, 1ex, on alfalfa; 12.IV.1993, SN, Al Saleh, 1ex; 25.XII.2003, SN, Al Yaaqub, A., 1ex; 13.III.2003, SN, Al Shathri, B., 2exs; 9.V, SN, Moshal, 2exs, on alfalfa; 10.III.1986, SN, 1ex;13.X.1986, 2exs, on alfalfa; 10.XI.1986, 1ex, on weeds; 25.XI.1990, Amro, 1ex, on alfalfa; 2.III.1989, Amro, 1ex, on grasses; 7.IV.1987, Amro, 2exs, alfalfa; 24.X.1992, 1ex, on alfalfa; 15.VIII.1993, Aseri, M., 1ex, on alfalfa; 19.XII.2010, SN, Setyaningrum, H. and Al Dhafer, H., 1ex; 30.XII.2009, SN, Sofan, A., 6exs; 31.X.2010, SN, Al Ansi, A., 9exs; 17.X.2010, SN, Setyaningrum, H., 1ex; 14.III.2010, SN, Al Dhafer, H.; El Gharabawy, A. and El Torkey, A., 2exs; Al Waseel, 24°48.40'N, 46°30.42'E, 1.XII.2010, SN, Al Abdul Muhsen, N., 1ex; 11.X.2012, SN, Hussain, S., 1ex; 1.X.2012, SN, Sonbati, S., 1ex; 14.III.2012, SN, Al Atwi, Z., 1ex; 14.III.2012, SN, Al Edwani, M., 1ex; 11.X, 1ex, on weeds; 11.X, Aseri, H., 1ex, on alfalfa; 12.XI.2008, Al Qahtani, M. 2exs, on alfalfa; 25.III.2004, Al Aati, 1ex, on alfalfa; 7.XI.2007, SN, Al Salman, 1ex; 20.XI.2011, SN, Al Sahwan, K., 1ex; 12.XI.2008, SN, Namosi, S., 2exs; 1.XII.2010, S.N.,Al Harbi, S., 1ex; 27.V.2009, SN, Adyatma, 1ex; 21.XI.2009, SN, Al Ansi, A., 1ex; 12.XI.2008, SN, Bel Hareth, H., 1ex; Al Uyaynah, 24°53'N, 46°22'E, 28.IV.2010, SN, Al Yusif, A., 1ex; 10.III.2010, SN, Al Salem, B., 1ex; 28.IV.2010, SN, Al Hashel, A., 1ex; 10.III.2010, SN, Al Hashel, A., 1ex; 1.XII.2010, SN, Al Ghunaim, A., 1ex; 3.XI.2010, SN, Al Ghunaim, A., 1ex; 27.IX.2003, SN, Mostafa, 2exs; 3.XI.2010, SN, Abdul Mohsen, 1ex; 30.XI.2010, SN, Al Dhafer, H. and Al Ansi, A., 1ex; 9.IV.2008, SN, Al Turk, 3exs; 26.II.2004, SN, Derwish, K., 1ex; 9.IV.2008, SN, Al Shahrani, 1ex; 27.II.2008, SN, Al Shamrani, 1ex; 12.X.2011, Al Husani, 2exs, on alfalfa; 24.X.2007, SN, Al Salman, 1ex; 18.IX.2011, SN, Al Amro, S., 2exs; 18.IX.2011, SN, Al Turki, I., 1ex; 12.X.2011, SN, Al Omar, A., 3exs; 19.X.2011, SN, Al Omar, A., 1ex; 8.VI.1993, 1ex, on alfalfa; 25.IX.1993, 1ex, on alfalfa; 6.IV.1993, 1ex, on weeds; 12.X.2011, SN, Al Hamad, Y., 1ex; 15.XII.2010, SN, Al Dhahbani, I., 2exs; 5.VIII, SN, Aiman, 1ex; 7.II.2005, SN, Al Oqail, 1ex; 8.II.2012, SN, Al Ghamdi, M., 1ex; 8.II.2012, SN, Al Wajaan, A., 1ex; 18.IX.2011, SN, Al Amro, S., 1ex; Wadi Ad Dawasir-As Sullayil Rd., 20°25.30'N, 45°05.65'E, 632 m, 17.I.2013, Al Ansi et al., 1ex; Wadi Ad Dawaser Nadec Company, 3.X.2012, SN, Al Dryhim, Y., 24exs, on alfalfa; **Tabuk**: Tabuk-Dhuba Rd., 28°18.39'N, 36°02.87'E, 824 m, 15.IX.2011, SN, Al Ansi et al., 6♂40exs; Tabuk-Madinah Rd., 28°23.47'N, 36°51.96'E, 808 m, 14.IX.2011, SN, Al Ansi et al., 1♀1ex; 14.IX.2011, SN, 11exs; **Qaseem**: 1.I.2001, SN, Al Jamhan, 2exs; Sakaka, 29°58.15'N, 40°12.18'E, 21.XI.2003, SN, Al Aqal, F., 1ex.

###### Local distribution.

Specimens of this species are found throughout the kingdom and were collected from Asir, Baha, Eastern province, Makkah, Riyadh, Tabouk, and Qaseem in the present study. This species was previously listed from Saudi Arabia by Martin (1972) and Walker and Pittaway (1987) and reported by Beccari (1971) from Riyadh, Talhouk (1982) from Riyadh and Makkah, and Abdel-Dayem et al. (2017) from Riyadh.

###### World distribution.

**E**: GR, IT, and ST; **N**: AG, AZ, EG, LB, and TU; **A**: AE, AF, ES, GAN, GUI, HEB, IN, IQ, PAL, JO, KI, KU, KZ, MG, NMO, NIN, PA, SA, SCH, SHA, SHX, SI, SY, TR, UP, WS, and XIN (Kovář 2007).

#### *Harmonia* Mulsant, 1846

##### Harmonia axyridis

Taxon classificationAnimaliaColeopteraCoccinellidae

(Pallas, 1773)

6CC06C88-5497-536D-8A60-84E003DF933B

Coccinella axyridis Pallas, 1773: 716.

###### Remark.

This species is a predator of aphids (Koch 2003).

###### Material examined.

**Riyadh**: Ad Diriyah, 24°40'N, 46°35'E, 4.V.2005, SN, Turkestani, S., 1ex.

###### Local distribution.

This species was recently recorded by Biranvand et al. (2019) from Riyadh as a new record to SA.

###### World distribution.

**Asia**: ES, FE, GAN, GUA, GUI, GUX, HKG, HEB, HEI, HEN, HKG, HUB, HUN, JA, JIA, JIL, JIX, KZ, LIA, MG, NC, NIN, NMO, SC, SCH, SD, SHA, SHN, SHX, TAI, WS, XIN, YUN, and ZHE; introduced to **Europe**: CZi, FRi, GBi, and GEi; **NAR** and **ORR** (Kovář 2007); SA (Biranvand et al. 2019), widely distributed as an invasive species in most continents (Roy et al. 2016).

#### *Hippodamia* Chevrolat, 1836

##### Hippodamia variegata

Taxon classificationAnimaliaColeopteraCoccinellidae

(Goeze, 1777)

6586C2DB-46B6-55F4-B3E7-865BA28560C8

Adonia variegata Goeze, 1777: 247.

###### Remark.

This is a common species found almost throughout Saudi Arabia in a wide range of habitats. Both larvae and adults of this species are known as predators of aphids.

###### Material examined.

**Asir**: Abha, Sodah, 18°16.27'N, 42°21.52'E, 24.IV.2011, SN, Al Ansi, A., 3exs; Al Majardah, Wadi Yabah, 19°16.27'N, 41°48.46'E, 411, 2.VI.2012, SN, Al Dhafer et al., 1ex; 1.III.2012, LT, 1ex; Khamis Mushait, Wadi Bisha, 18°20.01'N, 42°42.13'E, 1990 m27.IV.2011, SN, Al Ansi, A., 6exs; Wadi Abha, 18°22.03'N, 42°50.82'E, 1990 m, 28.IV.2011, SN, Al Ansi, A., 15exs; Khamis Mushait, 18°18'N, 42°45'E, 4.X.2005, SN, Hafedh, A., 1ex; **Baha**: Wadi Turubah, 20°14.37'N, 41°15.23'E, 10.V.2011, SN, Fadl, H., 5exs; 14.V.2011, SN, Fadl et al., 14exs; 1.VI.2011, SN, Al Dhafer et al., 1ex; Thee Ain, 19°55.78'N, 41°26.60'E, 741 m, 11.V.2011, SN, Fadl et al., 11exs; 3.VI.2012, SN, Al Ansi, A., 1ex; 15.V.2011, SN, Fadl et al., 4exs; Wadi Galah, 20°08.08'N, 41°20.56'E, 16.V.2011, SN, Fadl et al., 37exs; Baha Mountain, 25.V.2011, SN, El Hawagry, M., 22exs; Wadi Qanonah, 19°24.67'N, 41°36.39'E, 348 m, 11.V.2011, SN, Fadl et al., 15exs; Raghadan, 20°34.25'N, 41°45.11'E, 13.V.2011, SN, Fadl et al., 1ex; **Eastern Province**: Al Ahsa, 22°17.53'N, 50°40.46'E, 4.III.2011, SN, Al Ansi, A., 1♂50exs; 4.III.2011, MT, Al Ansi, A., 4exs; 4.III.2011, SN, Al Ansi, A., 22exs, on alfalfa; An Nuayriyah, 27°25.26'N, 48°27.20'E, 67m, 2.III.2011, SN, Al Ansi, A., 7exs, on weeds; Qatif, 26°45'N, 49°58'E, 21.II.1980, 1ex; **Hail**: Tayma-Hail Rd., 27°43.51'N, 38°12.94'E, 913 m, 16.IX.2011, SN, Al Ansi, A., 8exs; **Makkah**: Al Shefa-Taif Rd., 21°10.33'N, 40°23.24'E, 21°08.21'N, 40°21.50'E, 3.VI.2011, SN, Al Dhafer et al., 2exs; **Riyadh**: Al Ammariah, 24°49'N, 46°26'E, 18.IV.2007, SN, Ben Jaber, A., 1ex; 14.V.2007, SN, 1ex; Riyadh; 23.I, SN, 1ex; 22.XI.2012, SN, Al Atwi, M., 7exs; 22.XI.2012, SN, Al Agmi, B., 3exs; 29.XII.2008, SN, Abdel-Gayed, A., 2exs; 1.I.2010, SN, Al Ansi, A., 28exs; 11.XI.2009, SN, Al Ansi, A., 6exs; 26.XI.2004, SN, Al Abdulateef, 1ex; 22.XI.2012, SN, Al Wagaan, A., 1ex; 11.XI.2009, SN, Al Shamrani, S., 2exs, on vegetables; 15.III.2011, SN, Setyanengrum, H., 1ex, on weeds; 21.X.2010, SN, Koko, 2exs; 3.V.2011, LT, Koko, 2exs; 22.V.2011, SN, Al Ahmari, S., 6exs; 19.V.2009, SN, Ba Zeiad, A., 1♀2exs; 28.I.2009, SN, 1ex; 18.XI.2009, SN, Setyanengrum, H., 1♂2exs; 13.III.2013, SN, Al Qahtani, F., 5exs; 17.IV.2013, SN, Al Qahtani, F., 2exs; Rawdhet Khoraim, 25°22.98'N, 47°16.71'E, 559 m, 201exs were collected by SN and B on branches of *A. ehrenbergiana*, *A. gerrardii*, *C. proceraL. shawii*, *R. stricta* and *Z. nummularia*; and PT under canopy of the previous plants; also by MT and LT; through IV.2011; I, III, IV.2012; III.2013; 9.XI.2008, HP, Mutairy, T., 1ex, on weeds; Wadi Namar, 24°32.18'N, 46°34.60'E, 29.II.2012, HP, Al Ansi, A., 2♂3exs, feed on aphids; 6.IV.2012, SN, Al Othman, A., 9exs; Hutet Bani Tamim, 23°27.26'N, 46°41.13'E, 8.XI.2010, SN, Al Dhafer et al., 2exs; 28.V.2010, SN, Al Dhafer et al., 1ex, on *C. procera*; 7.V.2012, SN, Al Ansi, A., 25exs; 4.IV.2008, SN, Al Dhafer et al., 1ex; 15.IV.2010, HP, Al Othman, A., 1ex, on Alfalfa; 10.XI.2011, SN, Al Abdullah, I., 1ex; Al Hariq, 8.V.2012, HP, Abdel-Dayem, M., 1ex; Shuaib Huraymila, 25°06.10'N, 46°04.22'E, 804 m, 22.IV.2012, HP, Abdel-Dayem, M., 1ex; 26.V.1993, SN, Asseri, M., 1ex; Huraymila, 25°09'N, 46°08'E, 4.VI.1993, SN, Asseri, M., 1ex; 22.IV.2012, HP, 1ex; 4.VI.1993, SN, Hatem, 1ex; Al Haair, 24°27'N, 46°51'E, 21.IV.2009, HP, Al Ansi, A., 2exs; 13.V.2009, SN, Al Ansi, A., 3exs; 1.II.2009, SN, Al Harbi, S., 1ex; Sajir, 24°51.23'N, 45°42.39'E, 17.XI.2011, SN, Setyanengrum, H., 5exs; Al Muzahmiyah Al Khararah, 24°24.21'N, 46°14.40'E, 29exs were collected by SN and LT; III-V, VIII, X.2011; IV.2012; 31.III.1993, SN, Al Saleh, 1ex, on weeds; Al Uyaynah, 24°53'N, 46°22'E, 27.II.2013, SN, Al Qahtani, F., 5ex; 13.III.2013, SN, Al Qahtani, F., 4exs; 15.XII.2010, SN, Al Abdul Muhsen, 2exs; 30.XI.2010, SN, Al Ansi, A., 1ex, on alfalfa; 3.V.2011, SN, Al Ahmari, A., 3exs; 25.VI.2011, SN, Al Hazmy, 3exs; 13.III.2011, SN, Al Shehri, 1ex; 14.V.2011, SN, Al Hazmy, 1ex; 9.V.2011, LT, Al Ahmari, A., 1ex; 23.III.2011, SN, Al Ahmari, A., 3exs; 22.V.2011, SN, Al Ahmari, A., 2exs; 28.IV.2010, SN, Al Hashel, A., 4exs; 7.IV.2010, SN, Al Dawsari, M., 1ex; 10.III.2010, SN, Al Hashel, A., 1ex; 10.III.2010, SN, Al Haori, M., 5exs; 10.III.2010, SN, Al Mutairy, A., 3exs; 10.III.2010, SN, Al Othman, A., 6exs; 10.III.2010, SN, Al Otaibi, A., 4exs; 12.V.2010, SN, Al Hashel, A., 1ex; 10.III.2010, SN, Al Salem, B., 1ex; 7.IV.2010, SN, Al Mutairy, A., 1ex; 28.IV.2010, SN, Al Otaibi, A., 2exs; 10.III.2010, SN, Al Hazazi, T., 2exs; 28.IV.2010, SN, Al Othman, A., 1ex; 27.II.2008, SN, Al Amodi, 2exs; 14.III.2007, SN, Al Ahmadi, M., 3exs; 7.IV.2010, SN, Al Yousif, A., 2exs; 27.II.2008, SN, Al Tork, 2exs; 7.V.2008, SN, Al Tork, 2exs; 9.IV.2008, SN, Al Sharani, 2exs, on alfalfa; 28.IV.2009, SN, Al Ansi, A., 3exs; 30.XI.2010, SN, Al Ansi, A., 3exs; 12.V.2010, SN, Al Yousif, A., 3exs; 7.IV.2010, SN, Al Salem, B., 1ex; 24.II.2010, SN, Al Yousif, A., 1ex; 10.III.2010, SN, Al Dawsari, M., 1ex; 28.IV.2010, SN, Al Yousif, A., 1ex; 15.II.2010, SN, Al Yousif, A., 3exs; 15.XII.2010, SN, Al Harbi, S., 2exs; 3.XI.2010, SN, Al Ghunaim, A., 1ex; 15.XII.2010, SN, Al Dhabani, L., 1ex; 1.XI.2010, SN, Al Ghunaim, A., 1ex; 12.X.2011, SN, Al Omar, 2exs; 28.IX.2011, SN, Al Omar, 1ex; 13.III.2007, SN, Al Raqbah, R., 1ex; 12.X.2005, SN, Al Yami, M., 1ex; 4.VI.1993, SN, 2exs, on weeds; 6.V.1993, SN, 2exs, on weeds; 2.IV.1993, SN, 2exs, on weeds; 8.VI.1993, SN, 2exs, on weeds; 4.V.1993, SN, 1ex, on weeds; 25.V.1993, SN, 1ex, on weeds; 25.II.2004, SN, Hatem, 1ex, on alfalfa; 7.III.2012, SN, Al Edwani, M., 5exs; 4.IV.2012, SN, Al Ghamedi, R., 1ex; 8.II.2012, SN, Al Ghamedi, R., 4exs; 7.III.2012, SN, Al Ghamedi, R., 10exs; 8.II.2012, SN, Al Qahtani, R., 14exs; 4.IV.2012, SN, Al Wagaan, A., 2exs; 7.III.2012, SN, Al Atwi, M., 4exs; 8.II.2012, SN, Al Wagaan, A., 4exs; 8.II.2012, SN, Al Agmi, B., 2exs; 7.III.2012, SN, Al Agmi, B., 2exs; 4.IV.2012, SN, Al Agmi, B., 1ex; Ben Jabr, 15.V.2011, LT, Al Shahri, A., 2exs; Al Waseel, 24°48.40'N, 46°30.42'E, 12.XI.2008, SN, Ba Zeiad, A., 1♂, on alfalfa; 5.V.2004, SN, 1ex, on alfalfa; 13.IV.1993, SN, 1ex; 14.III.2012, SN, Al Atwi, M., 5exs; 14.III.2012, SN, Al Wagaan, A., 1ex; 14.III.2012, SN, Al Edwani, M., 2exs; 21.XI.2009, SN, Al Ansi, A., 13exs; 21.XI.2009, SN, Single, A., 2exs; 20.X.2011, SN, Al Matter, E., 1ex; 20.X.2011, SN, Al Rashedi, 1ex; 20.XI.2011, SN, Al Matter, E., 4exs; 26.V.2010, SN, Al Yousif, A., 1ex; 26.V.2010, SN, Al Othman, A., 1ex; 1.XII.2010, SN, Al Dhahbani, L., 1ex; 11.V.2010, SN, Husain, M., 1ex; Wadi Laban, 24°38.39'N, 46°37.20'E, 21.XI.1990, SN, Al Mutlaq, 1ex, on vegetables; Riyadh, 6.XII.2006, ST, 1ex; Nesah, 24°43.32'N, 46°41.12'E, 30.XI.2005, SN, 1ex; Al-Quway’iyah, 24°20'N, 45°09'E, 18.V.2012, SN, Al Ghamedi, R., 12exs; Tumayer, 25°42.36'N, 45°52.11'E, 9.V.2011, LT, Al Ahmari, S., 9exs; 10.II.2010, MT, Al Dhafer, H., 1ex; 25.I.2010, MT, Al Dhafer, H., 1ex; Al Kharj, Ar Rafai, 20°24'N, 46°29'E, 4.IV.2007, SN, Shraf Addeen, A., 9exs, on alfalfa; 16.V.2007, SN, Al Grid, S., 4exs; 4.IV.2007, SN, Al Shehri, 3exs; Al Kharj, 20°24'N, 46°29'E, 14.XI.2007, SN, Al Salman, 3exs; 26.XI.2008, SN, Valenza, B., 1ex; 1.IV.2009, SN, Al Zahrani, M., 2exs; 23.XII.2008, SN, Setyanengrum, H., 1ex, on vegetables; 24.III.2010, SN, Al Haori, M., 2exs; 24.III.2010, SN, Al Dawsari, M., 1ex; 24.III.2010, SN, Al Mutairy, A., 1ex; 9.III.2011, SN, Al Hazmy, H., 1ex; 1.IV.2009, SN, Al Amri, A., 17exs; 2.V.2007, SN, 3exs; 1.IV.2009, SN, Al Sebiai, M., 1ex; 2005, SN, 2exs; Ad Diriyah, 24°40'N, 46°35'E, 23.XI.2011, LT, Al Kassem, A., 1ex; 23.X.1989, SN, 1ex; 8.XII, SN, 1ex, on alfalfa; 18.III.2009, SN, Al Sebiai, M., 4exs, on alfalfa; 29.X.2008, SN, Al Ahmari, A., 1ex, on alfalfa; 11.III.2009, SN, Al Harbi, M., 5exs; 2.VI.2009, SN, Al Zahrani, M., 1ex; 29.X.2008, SN, Ba Zeiad, A., 2exs, on alfalfa; 5.XII.2010, SN, Abu Al Raha, W., 2exs; 8.X.2000, SN, Hatan, 2exs; 14.X.2003, SN, Al Dhubaib, 1ex; 9.X, SN, Mustafa, 1ex; 15.X.2003, SN, Al Yaqoob, 1ex; 14.X.2003, SN, Al Saiary, 1ex; 26.IX.2011, SN, Al Omar, 1ex; 1.XII.2010, SN, Abdel Gayed, A., 1ex; 29.X.2008, SN, Al Qahtani, M., 1ex, on alfalfa; 23.XI.2011, LT, Al Kassem, A., 1ex; 23.IV.2009, SN, Husain, M., 1ex; 14.V.2009, SN, Sofan, A., 1ex; 7.IV.2010, SN, Al Salem, B., 1ex; 7.IV.2010, SN, Hazazi, T., 1ex; 17.III, SN, 5exs, on vegetables; 24.III, SN, 10exs, on vegetables; 22.III, SN, 1ex, on vegetables; 15.X.2000, SN, Al Khurigi, M., 1ex; 22.XI.1990, SN, Asseri, M., 1ex, on alfalfa; 25.IX.2005, SN, 1ex, on alfalfa; 1.XI, SN, Al Qarni, A., 3exs; 20.X.1993, SN, Al Aseri, 2exs, on alfalfa; 5.VIII, SN, Al Qarni, A., 1ex; 17.III.1989, SN, 1ex, on barley; 16.X.1989, SN, 1ex, on alfalfa; 15.IV. SN, Adel, 1ex, on vegetables; 20.XI.1991, SN, Ghazi, 1ex, on alfalfa; 30.X.1989, SN, 1ex, on barley; 29.III.1989, SN, 4exs, on Barley; 14.IV.1993, SN, Aziz, A., 4exs, on Alfalfa; XI.1989, SN, 1ex, on alfalfa; V.1986, SN, 1ex, on alfalfa; 14.XII.1992, SN, 1♀3exs, on alfalfa; 22.XI.1991, SN, Ghazi, 1ex; 17.V.1989, SN, 1ex; 7.XI.1990, SN, Al Qarni, A., 2exs, on vegetables; 20.IV.1992, SN, Saif, A., 2exs, on alfalfa; 26.IV.1992, SN, Saif, A., 2exs; 9.IV.2003, SN, Al Habeeb, A., 1ex; 5.VI.1993, SN, Aziz, A., 1ex; 25.IX.2005, SN, 1ex; 21.IV, SN, Nabeel, 1ex; 30.X.1989, SN, 1ex, on alfalfa; 4.XII.1992, SN, 1ex, on alfalfa; 30.XII.1989, SN, Amro, 1ex, on vegetables; 7.XI, SN, Al Jaber, 1ex, on vegetables; Education Farm, 24°40'N, 46°35'E, 18.V.2013, SN, Al Qahtani, F., 1ex; 25.X.2009, SN, Al Ahmari,S., 1ex, on alfalfa; Ad Diriyah Erqah, 11.II, SN, Al Issa, M., 2exs; 16.XI.1989, SN, 1ex, on alfalfa; 21.XI, SN, Al Jaberi, 2exs, on vegetables; 10.III.1987, SN, 1ex, on alfalfa; 12.IV.1993, SN, 1ex; 9.I, SN, 1ex, on alfalfa; 18.V.1993, SN, 2exs, on alfalfa; 4.V.1978, SN, 1ex, on alfalfa; 9.V, SN, Moshal, 1ex, on alfalfa; 17.III.2004, SN, Al Hugail, Y., 1ex; 12.III.2003, SN, Al Awadhi, A., 1ex, on alfalfa; 24.III.2003, SN, Al Harbi, 1ex, on alfalfa; 12.III.2003, SN, Al Habeeb, A., 1ex, on alfalfa; 4.III, SN, 1ex, alfalfa; 9.I, SN, 1ex, on alfalfa; 16.III.2005, SN, 1ex; 4.VIII, SN, 1ex; 17.V.2011, SN, Al Etwy, Y., 1ex; 23.X.1992, SN, 1ex, on alfalfa; 30.XII.2009, SN, Sofan, A., 2exs; 21.IV.1987, SN, 2exs, on alfalfa; 5.V.1987, SN, 1ex, on alfalfa; 4.V.1987, SN, 1ex, on alfalfa; 8.XI.1989, SN, 1ex, on alfalfa; 12.IV.1993, SN, 1ex, on alfalfa; 23.IV, SN, 1ex, on alfalfa; 7.IV.1987, SN, 11exs, on alfalfa; 15.XI.2006, SN, Al Qahtani, A., 2exs, on alfalfa; 19.V.2009, SN, Al Motaeb, M., 9exs, on alfalfa; 2.X.1985, SN, 1ex, on alfalfa; 15.XI.2006, SN, Al Dawood, A., 1ex; 16.V.2007, SN, Shraf Addeen, A., 1ex, on alfalfa; 8.VI.2009, SN, Belhareth, H., 1ex, on alfalfa; 2.VI.2009, SN, Al Ghamedi, R., 1ex; 25.VII.1998, SN, Al Qarni, A., 1ex; 5.I.1687, SN, Amro, 3exs, on alfalfa; 14.III.2010, ST, Al Dhafer, H.; El Gharabawy, A. and El Torkey, A., 1ex; 10.IV.2001, SN; 5.VII, SN, Al Qarni, A., 1ex; 29.XII.1986, SN, 1ex, on alfalfa; 19.I.1987, SN, Amro, 2exs, on weeds; 12.III.2003, SN, Al Shathri, 1ex; 12.I.1987, SN, 5exs; 31.III.1987, SN, 3exs; 12.IV.1993, SN, 1ex; 24.XI.1986, SN, 1ex;17.X.2011, SN, Al Khudairi, H., 1ex; 2.V.2010, SN, Al Dhafer et al., 4exs; 30.XII.2009, SN, Sofan, A., 1ex;19.XII.2010, SN, Al Ansi, A., 1ex, on alfalfa; 31.X.2010, SN, Al Ansi, A., 2exs, on alfalfa; 7.III.2011, SN, Abdel-Gayed, 4exs, on fig plant; 28.III.2010, PT, Al Dhafer et al.,1ex; 10.III.1987, SN, 1ex; 24.III.1987, SN, 1ex; 5.V.1987, SN, 1ex; Wadi Ad Dawaser Nadec Company, 3.X.2012, SN, Al Dryhim, Y., 8exs, on alfalfa; Riyadh, 11.IX.1969, SN, R. D. Pope, ANMA, 2exs, feed on aphids; same location 21.IV.1980, SN, Talhouk et al., ANMA, 16exs; same data except 13.V.1980, SN, ANMA, 8exs; **Tabuk**: Tabuk-Dhuba Rd., 28°18.39'N, 36°02.87'E, 824 m, 15.IX.2011, SN, Al Ansi, A., 1♂2♀; 15.IX.2011, SN, Al Ansi, A., 3exs; 15.IX.2011, SN, Al Ansi, A., 28exs; 15.IX.2011, SN, Al Ansi, A. 155exs, (preserved in alcohol); Tabuk-Madinah Rd., 28°23.47'N, 36°51.96'E, 808 m, 14.IX.2011, SN, Al Ansi, A., 10exs; 14.IX.2011, SN, Al Ansi, A., 8exs; 14.IX.2011, SN, Al Ansi, A., 1ex; 14.IX.2011, SN, Al Ansi, A., 4exs; 14.IX.2011, SN, Al Ansi, A., 1ex.

###### Local distribution.

Asir, Baha, Eastern Province, Makkah, Riyadh, and Tabuk. It was previously recorded from Jizan by Beccari (1971), from Riyadh and Makkah by Talhouk (1982) and by El-Hawagry et al. (2013), from Baha and Abdel-Dayem et al. (2017) from Riyadh and listed by Walker and Pittaway (1987).

###### World distribution.

**Asia**: AE, AF, BEI, BT, FE, FUJ, GAN, HEB, HEN, HP, HUN, IN, IQ, PAL, JIL, JO, KA, KI, KZ, LE, LIA, MG, NC, NMO, NIN, NP, PA, SA, SC, SCH, SHA, SHX, SD, SI, SY, TD, TM, TR, UP, UZ, WS, YE, YUN, XIN, and XIZ; **Europe**: AB, AE, AL, AN, AR, AU, BE, BH, BU, BY, CR, CT, CZ, DE, EN, FI, FR, GB, GE, GG, GR, HU, IT, LA, LS, LT, LU, MC, MD, NL, NT, PL, PT, RO, SK, SL, SP, ST, SV, SZ, TR, UK, and YU; **North Africa**: AG, CI, EG, LB, MO, MR, and TU; **AFR, NAR**, and **ORR** (Kovář 2007).

#### *Oenopia* Mulsant, 1850

##### Oenopia oncina

Taxon classificationAnimaliaColeopteraCoccinellidae

(Olivier, 1808)

EBBBB8A1-EF87-536F-BC4B-6FE258A95F9D

Coccinella oncina A. G. Olivier, 1808: 1048.

###### Remark.

Presence of this species could not be confirmed, although it was listed from the KSA by Kovář (2007).

###### World distribution.

**Asia**: AE, AF, GAN, IN, IQ, PAL, JO, KA, KI, KU, KZ, LE, MG, PA, SA, SI, SY, TD, TM, TR, and UZ; **Europe**: AB, AR, BU, GR, and TR; **North Africa**: EG (Kovář 2007).

#### *Psyllobora* Chevrolat, 1836

##### Psyllobora bisoctonotata

Taxon classificationAnimaliaColeopteraCoccinellidae

(Mulsant, 1850)

48B68F97-1EDE-50F7-A446-5AF96686FBFA

Thea bisoctonotata Mulsant, 1850: 204.

###### Remarks.

This species was found in a variety of habitats from greenhouses, open crop fields, and in natural habitats from low to highlands. Both adults and larvae of this species feed on fungi (Raimundo and van Harten 2000).

###### Material examined.

**Asir**: 8–20 km outside from Abha-Taif Rd., 1976, Wittmer et al., ANMA, 1ex; Abha, Sodah, 18°16.27'N, 42°21.52'E, 19.III.2009, SN, Al Dhafer et al., 5♂2♀, on squash plants; 24.IV.2011 SN, Al Ansi et al., 2♂; Abha, Raydah, 18°12.10'N, 42°24.54'E, 2578 m, 8.II.2016, SU, Al Ansi, A., 1ex; Ahd Rifidh, 18°06.33'N, 42°53.82'E, 16.I.2013 BS, Al Ansi et al., 7♀4♂; **Baha**: Thee Ain, 19°55.78'N, 41°26.60'E, 741 m, 15.V.2011, SN, Fadl et al., 1♂; 13.X.2010, SN, Al Dhafer et al., 1♂; 12.IV.2016, SU, Al Ansi, A., 2exs; Wadi Turubah, 20°14.37'N, 41°15.23'E, 14.V.2011, SN, Fadl et al., 1♂1ex; 27.IX.2013, Al Dhafer, H., 12exs; Al Atawlah, 20°18.37'N, 41°20.52'E, 2160 m, 24.IV.2013, BS, Al Ansi et al., 11exs; **Makkah**: Taif, 21°08'N, 40°58'E, 10.IX.1969, SN, ANMA, 4exs; **Najran**: Rijila, Wadi Najran, 17°31.56'N, 44°13.65'E, 1257 m, 15.I.2013, BS, Al Ansi et al., 20♂8♀; 15.I.2013, SN, Al Ansi et al., 1♂; Wadi Shuaib Barran, 17°28.94'N, 44°05.52'E, 1325 m, 16.I.2013, BS, Al Ansi et al., 1♂; **Riyadh**: Ad Diraiyah, 5.VIII.1983, Abu Thoria, ANMA, 1ex; 21.XII.1963, SN, Abu Thoria, ANMA, 1ex.

###### Local distribution.

This species is widely distributed in the KSA and was collected from Asir, Baha, Makkah, Najran, and Riyadh. It was previously reported by Shalaby (1962) from Makkah, Beccari (1971) from Makkah, Fürsch (1979) from Asir, and Talhouk (1982) from Makkah.

###### World distribution.

**Asia**: AE, AF, IN, IQ, PAL, JO, KA, PA, SA, SY, UP, and YE; **North Africa**: EG; **AFR** and **ORR** (Kovář 2007).

#### *Xanthadalia* Crotch, 1874

##### Xanthadalia effusa rufescens

Taxon classificationAnimaliaColeopteraCoccinellidae

(Mulsant, 1850)

ACF2920B-FE16-54CB-A574-542EA80BE1D0

Xanthadalia effusa (Erichson, 1843)
Harmonia rufescens Mulsant, 1850: 76.

###### Remark.

This is a rare species, limited to only Afrotropical region; its presence in the KSA was not confirmed, and only Kovář (2007) enlisted it from the country.

###### World distribution.

**Asia**: SA and YE; **North Africa**: EG; **AFR** (Kovář 2007).

#### Epilachnini Mulsant, 1846

***Henosepilachna* Li, 1961**

##### Henosepilachna hirta

Taxon classificationAnimaliaColeopteraCoccinellidae

(Thunberg, 1781)

965E1101-62F6-5D9B-A618-8B2B4E85C885

Coccinella hirta Thunberg, 1781: 23.

###### Remark.

This species was found from high elevated lands of Asir province and exhibited an Afrotropical distribution. It is a phytophagous coccinellid.

###### Material examined.

**Asir**: Abha, 18°14'N, 42°31'E, 27–28. VIII.1978, Shalaby, F., ANMA, 2exs; Abha Raydah, 18°12.10'N, 42°24.54'E, 2578 m, 4.XI.2013, HP, Abdel-Dayem et al., 1♀; 8.II.2016, SU, Al Ansi, A., 3♀5♂.

###### Local distribution.

Specimens of this species were collected from Raydah mountains of Asir and previously reported by Shalaby (1962) from Asir and by Beccari (1971) from Asir.

###### World distribution.

**Asia: SA** (Shalaby 1962; Beccari 1971); South, West, and Central Africa (Fürsch 1991).

#### *Chnootriba* Chevrolat, 1837

##### Chnootriba elaterii orientalis

Taxon classificationAnimaliaColeopteraCoccinellidae

(Zimmermann, 1936)

81715A81-5234-538F-B9BB-04E7795C2CFF

Coccinella elaterii Rossi, 1794
Coccinella chrysomelina Fabricius, 1775 (1): 277.

###### Remark.

This is a phytophagous coccinellid. Members of this species were found to be associated with squash and cucurbits. This species is phytophagous and has been reported as a serious pest of Cucurbitaceae (Raimundo and van Harten 2000).

###### Material examined.

**Asir**: Khamis Mushait Wadi Bin Hashbal, 18°35.44'N, 42°39.01'E, 1892 m, 26.IV.2011, SN, Al Ansi, A., 1ex; Khamis Mushait, 18°18'N, 42°45'E, 17.IV.2016, SN, Soliman, A., 1ex; Abha Raydah, 18°12.10'N, 42°24.54'E, 2578 m,5.XII.2013, HP, Khan, S., 1ex; 16.IV.2016, HP, Al Dhafer, H., 1ex; Thalooth Al Mandhar Wadi Baqrah, 18°47.57'N, 42°01.12'E, 433 m, 12.X.2013, HP, El Torkey, A., 6exs; HP, Rasool, I., 2exs; **Baha**: Wadi Ghanuna, 19°24.67'N, 41°36.39'E, 348 m, 11.V.2011, SN, Fadl, H., 2exs; Thee Ain, 19°55.78'N, 41°26.60'E, 741 m, 11.V.2011, SN, Fadl, H., 1ex; 11.IV.2016, SN, Al Ansi, A., 1ex; Wadi Al Arg Qilwah-Adhom Rd., 20°29.98'N, 40°48.95'E, 429 m, 9.XI.2012, BS, Fadl, H., 1♂1ex; **Jizan**: Baysh, 17°42.71'N, 42°53.19'E, 102 m,23.II.2015, HP, Al Harbi et al., 1ex; Wadi Gowra Aiban, 17°17.57'N, 43°04.21'E, 451 m, 11.XI.2012, SN, Fadl, H., 1ex; Jizan city, 16°54'N, 42°29'E, 20.I.1979, SN, Talhouk et al., ANMA, 12exs, det. Fürsch, 1979. **Makkah**: Al-Taif, 21°08'N, 40°58'E, 19.II.1980, 2exs; **Riyadh**: Hutet Bani Tamim, 23°27.26'N, 46°41.13'E, 30.XII.2010, HP, Al Dryhim et al., 2exs; 2.VI.2007, SN, Al Dryhim et al., 1ex; Huraymala, 27.VI.1988, SN, Al Dawood, A., 13exs, on weeds; 8.V.1987, 3exs, on cucurbits; 14.IV, 5exs; Al Dawadmi, 24°30'N, 44°24'E, 22.II.1980, 1ex; Dirab, 23°30'N, 46°51'E, 17.XI.1989, 1ex; 17.XI.1986. Amro, 1ex, on lantana; 17.XI, SN, Al Fehaid, M.,1ex; Al-Hair, 13.I.1989, Salem, 9exs, on colocynth; Al Kharj, 20°24'N, 46°29'E, 31.X.2002, Saif, A., 1ex, on Squash; Tumayer, 25°42.36'N, 45°52.11'E, 9.V.2011, LT, Al Ahmari, S., 1ex; Riyadh, 2.XI.2013, HP, Salman, S., 1ex; **Qaseem**: Buraydah 26°12.954'N, 44°02.48'E, 633 m, 17.IX.2011, SN, Al ansi et al., 1ex.

###### Local distribution.

This species is widely distributed in Saudi Arabia and was collected from Asir, Bahah, Jizan, Makkah, Riyadh, and Qaseem. It was previously recorded from Zilfi approximately 160 miles northwest of Riyadh by Kapur (1959); from Eastern Province, Riyadh, Makkah, and Tabouk by Beccari (1971); and from Riyadh by Fürsch (1979) and listed by Martin (1972) and Walker and Pittaway (1987).

###### World distribution.

**Asia**: AF, CY, IN, IQ, PAL, JO, SI, SA, SY, PA, TR, and YE; **North Africa**: EG and LB; **AFR** (Kovář 2007).

#### Noviini Mulsant, 1850

##### Novius

Taxon classificationAnimaliaColeopteraCoccinellidae

Mulsant, 1850

A44285D6-9DE7-5E54-9D3E-57BD9B70AE49

###### Remark.

The species of genus *Rodolia* have been placed in *Novius* based on the latest paper by Pang et al. (2020).

##### Novius argodi

Taxon classificationAnimaliaColeopteraCoccinellidae

(Sicard, 1909)

1926E388-D947-558E-A440-762AFDE91927

Rodolia argodi Sicard, 1909: 142.

###### Remark.

This species is known as a predator of scale insects, including *Icerya purchasi* Maskell, as mentioned in the label data by Fürsch (1968); it is a pest of *Citrus* spp. and *Casuarina* sp. (Martin 1972).

###### Material examined.

**Riyadh**: Specimen was taken from ANMA collection that was collected from Riyadh city, V.1966, ANMA, 1ex, det. Fürsch 1968.

###### Local distribution.

This species was listed by Martin (1972) and reported from Asir and Makkah by Abu-Thuraya (1982).

###### World distribution.

**Asia**: SA (Martin 1972; Abu-Thuraya 1982); it is widespread in Africa (Raimundo and van Harten 2000); AE (Raimundo et al. 2007).

##### Novius cardinalis

Taxon classificationAnimaliaColeopteraCoccinellidae

(Mulsant, 1850)

5ED13A28-C219-510C-89D7-0F06EF3F2258

Vedalia cardinalis Mulsant, 1850: 906.

###### Remark.

This species became famous at the end of the 19^th^ century when it was introduced from its native country Australia to California for the control of cottony cushion scale (*I. purchasi*) in citrus orchards, producing the first major success in biological control. This species is now introduced to all continents, except Antarctica (Raimundo and van Harten 2000).

###### Material examined.

**Asir**: Khamis Mushait, Wadi Baisha, 27.IV.2011, SN, Al Ansi, A., 1♀; **Baha**: Al Mandaq, Wadi Turubah, 20°14.37'N, 41°15.23'E, 3.VI.2012, BS, Al Ansi, A., 2♂1♀; Raghdan, 20°34.25'N, 41°45.11'E, 16.V.2010, SN, Al Dhafer et al., 1♀; Rahwan, 20°04.03'N, 41°27.01'E, 2272 m, 24.V.2013, BS, Al Ansi et al., 2exs; King Saud Rd., 20°00.28'N, 41°27.91'E, 2132 m, 25.V.2013, BS, Al Ansi et al., 3exs.

###### Local distribution.

Specimens of this species were collected from Asir and Baha provinces and previously reported from Najran by Abu-Thuraya (1982).

###### World distribution.

**Asia**: AE, FUG, HAI, GUA, HEN, HKG, HUB, IN, PAL, JA, JIA, JIX, JO, PA, SC, SCH, SHA, SHG, TAI, TR, YUN, and ZHE; **Europe**: AL, BU, CR, FR, GR, IT, MA, PT, SP, and TR; **North Africa**: CI, AG, EG, LB, MO, and TU; **AFR**, **AUR**, **NAR**, **NTR**, and **ORR** (Kovář 2007).

##### Novius yemenensis

Taxon classificationAnimaliaColeopteraCoccinellidae

(Raimundo & Fürsch, 2006)*

25DBD7BE-C99F-5EBD-80B1-A96093E67136

Figure 3

Rodolia yemenensis Raimundo & Fürsch, 2006: 219–220.

###### Diagnosis.

This species is restricted to the Arabian Peninsula and can be identified by the following characters: body entirely reddish to reddish brown, some specimens have blackish head and pronotum, without black patterns on elytra and with white semi-erect short setae. Punctures on elytra are larger than those on pronotum. Base of elytra is slightly concave with rounded shoulders.

**Figure 3.**
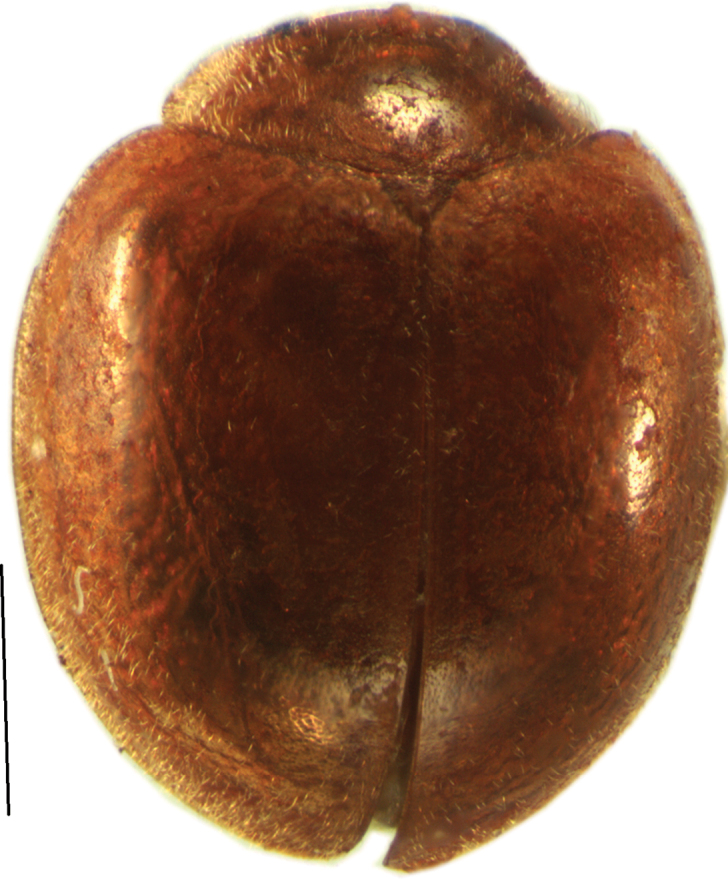
Dorsal view of *Novius yemenensis*. Scale bar: 1 mm.

###### Material examined.

**Asir**: Thalooth Al Mandhar Wadi Baqrah, 18°47.57'N, 42°01.12'E, 433 m, 12.X.2013, HP, Abdel-Dayem, M., 1♀; Rijal Almaa, Wadi Sabin, 17°48.25'N, 42°21.64'E, 194 m, 10.II.2016, SU, Al Ansi, A., 1♀; **Jizan**: Sabya-Hurub Rd., 17°16.94'N, 42°17.54'E, 97m, 24.V.2012, SN, Al Ansi, A., 1♂; **Riyadh**: El-Jola Riyadh-Makkah Rd., 24°20.42'N, 45°47.29'E, 11.X.2010, SN, Al Dhafer et al., 1♂; King Abdullah street, in front of King Abdulaziz city, 24 43.01'N, 46 38.65'E, 21.III.2010, HP, El Gharbawy, A., 1♂; Dirab, 23°30'N, 46°51'E, 1.XI.2009, ST, Al Dhafer et al., 1♀.

###### Local distribution.

This species was collected from Asir, Jizan, and Riyadh provinces.

###### World distribution.

**Asia**: YE (Raimundo et al. 2006); newly recorded from SA.

#### *Diomus* Mulsant, 1850

##### Diomus rubidus

Taxon classificationAnimaliaColeopteraCoccinellidae

(Motschulsky, 1837)

27202BD6-0DE0-5EC1-9C0A-CEC0EB5537C3

Scymnus rubidus Motschulsky, 1837: 418.

###### Material examined.

**Riyadh**: As Sullayyil-Najran Rd., 18°01.35'N, 42°45.81'E, 619 m, 17.I.2013, BS, Al Ansi, A., 1♂; Rawdhet Khoraim, 25°22.98'N, 47°16.71'E, 559 m, 14.IV.2012, PT, 1♂1♀, under *C. procera*, 14.IV.2012, PT, 1♂1♀, under *L. Shawii*; 23.VI.2012, PT, 1♀, under *C. procera*.

###### Local distribution.

It was collected from Riyadh (Abdel-Dayem et al. 2017) and previously reported by Fürsch (1979) from Riyadh.

###### World distribution.

**Asia**: IN, IQ, PAL, LE, SA, SY, and YE; **Europe**: AR, CR, FR, GR, and IT; **North Africa**: AG, EG, LB, MO, and TU (Kovář 2007); AE (Raimundo et al. 2007).

#### Hyperaspidini Mulsant, 1846

***Hyperaspis* Chevrolat, 1836**

##### Hyperaspis polita

Taxon classificationAnimaliaColeopteraCoccinellidae

Weise, 1855

C32CA13C-9B03-5FB4-AE20-43792F0FB580

Hyperaspis polita Weise, 1855: 60.

###### Remark.

This species is a predator of the mealybug *Nipaecoccus viridis* (Newstead, 1894) (Alizadeh et al. 2013).

###### Materials examined.

Riyadh, 2.II.1980, Talhouk et al., ANMA, 1♀.

###### Local distribution.

It was collected from Riyadh and previously listed in the taxonomic key by Fürsch (1979).

###### World distribution.

**Asia**: AF, CY, PAL, SY, TD, TM, and TR; **North Africa**: LB; **AFR** (Kovář 2007), SA (Fürsch 1979).

##### Hyperaspis pumila pumila

Taxon classificationAnimaliaColeopteraCoccinellidae

Mulsant, 1850

05164073-9661-5649-A970-B9BEB28C7DCC

Hyperaspis pumila Mulsant, 1850: 655.

###### Material examined.

**Asir**: Thee Ain, 19°55.78'N, 41°26.60'E, 741 m, 15.V.2011, SN, Fadl et al., 1♀; Thalooth Al Mandhar Wadi Baqrah, 18°47.57'N, 42°01.12'E, 433 m, 4.XI.2013, LT, Al Dhafer et al., 1♀; Muhail Wadi Hali, 18°30.12'N, 42°02.21'E, 440 m, 11.II.2016, SU, Al Ansi, A., 1♀; Dirab, 23°30'N, 46°51'E, 19.III.2014, PT, Al Harbi, M., 1♀, under palm tree.

###### Local distribution.

This species was collected from Asir, Baha, and Riyadh provinces. It was previously catalogued only by Kovář (2007) with no information about its distribution in the KSA.

###### World distribution.

**Asia**: SA and YE; **North Africa**: LB; **AFR** (Kovář 2007).

##### Hyperaspis vinciguerrae

Taxon classificationAnimaliaColeopteraCoccinellidae

Capra, 1929

CE6FEE33-CEB6-546D-930E-96B49E757F09

Hyperaspis vinciguarae Capra, 1929: 241.

###### Remark.

This species is a predator of aphids (Raimundo et al. 2007) and Pseudococcidae (mealybugs) such as *Maconellicoccus hirsutus* Green, 1908, and *Planococcus* sp., pests of *Ficus carica*, and on *Nipaecoccus vastator* (Maskell, 1895), a pest of *Citrus* spp. (Martin 1972).

###### Material examined.

**Asir**: Al Majardah, Wadi Al Talalie, 19°05.19'N, 41°47.78'E, 286 m, 1.VI.2012, BS, Al Ansi, A., 6♂; Wadi Yabah, 19°16.27'N, 41°48.46'E, 411, 15.IV.2016, SN, Al Ansi, A., 2♂1♀; Thee Ain, 19°55.78'N, 41°26.60'E, 741 m, 5.V.2015, HP, Soliman, A., 1♂; **Baha**: Wadi Ghanuna, 19°24.67'N, 41°36.39'E, 348 m, 11.V.2011, SN, Fadl et al., 2♂; **Jizan**: Farasan Island, 16°41.46'N, 42°07.22'E, 9.II.2012, SN, Deeming, J., 1♂; **Najran**: Hubuna Lahumah, 17°50.47'N, 44°16.82'E, 1212 m, 14.I.2013, BS, Al Ansi et al., 1♀; **Riyadh**: Dirab, Education Farm KSU, 24°40'N, 46°35'E, 24.IX.2011, LT, Fadl, H., 1♂; Ad Diriyah, 24°40'N, 46°35'E, 10.XI, SN, Farhan, 1♂; Rawdhet Khoraim, 25°22.98'N, 47°16.71'E, 559 m, II-VIII.2012, 13♂,1♀,1ex, using BS, MT, SN, and LT, on on *C. procera*, *Rhazya steica* and *L. shawi*; Al Ammariyah, 17.X.2013, SN, Sonbati, S., 1♀; Riyadh, 18.IV.1978, SN, Talhouk et al., ANMA, 3exs; 28.X.1977, SN, ANMA, 6exs; 15.IX.1977, SN, ANMA, 3exs; **Tabuk**: Tabuk-Dhuba Rd., 28°18.39'N, 36°02.87'E, 824 m, 15.IX.2011, SN, Al Ansi, A., 2♂; **Qaseem**: Buraydah, 26°12.954'N, 44°02.48'E, 633 m, 17.IX.2011, Al Ansi, A., 2♂.

###### Local distribution.

It is widely distributed in Saudi Arabia and was collected from Asir, Baha, Jizan, Najran, Riyadh, Tabuk, and Qaseem provinces. Before this study, it was reported by Fürsch (1979) from Riyadh, by Abu-Thuraya (1982) from Riyadh and Madinah, by Talhouk (1982) from Riyadh, and by Abdel-Dayem et al. (2017) from Riyadh; it was listed by Martin (1972).

###### World distribution.

**Asia**: SA and YE; **North Africa**: LB; **AFR** (Kovář 2007); AE (Raimundo et al. 2007), IN (Biranvand et al. 2017).

#### Scymnini Mulsant, 1846

***Clitostethus* Weise, 1885**

##### Clitostethus arcuatus

Taxon classificationAnimaliaColeopteraCoccinellidae

(Rossi, 1794)*

6705F6D6-8B97-5FDB-86AE-CB0FD8522D96

Figure 4

Coccinella arcuata Rossi, 1794: 88.

###### Remark.

This species is reported as a predator on the whitefly *Siphoninus phillyreae* (Haliday, 1835) (Hemiptera: Aleyrodidae) in Iran (Tavadjoh et al. 2010).

**Figure 4.**
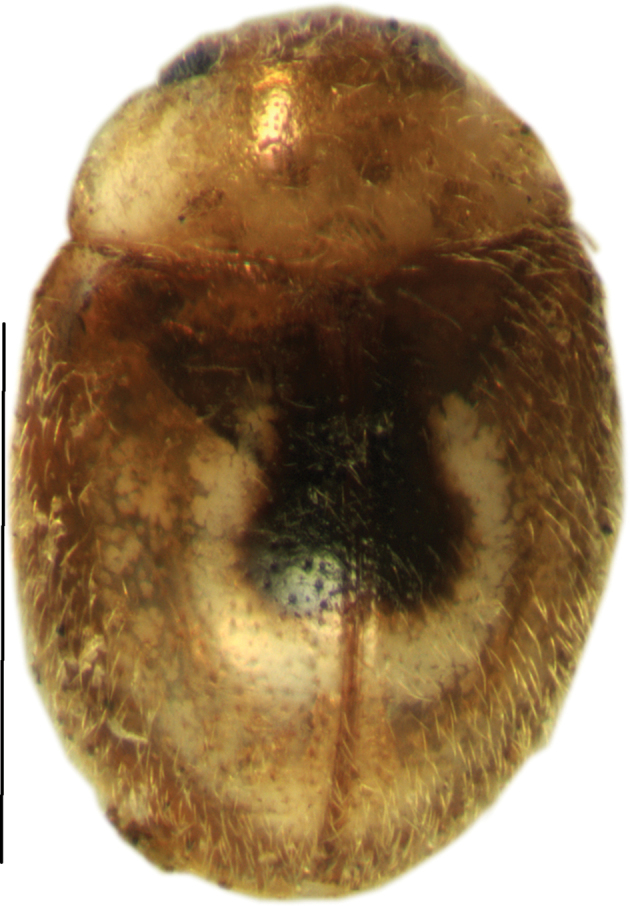
Dorsal view of *Clitostethus arcuatus*. Scale bar: 1 mm.

###### Diagnosis.

Body is dark brown to black, covered with rather long, whitish hairs; head is black; pronotum is whitish, at its middle with several black spots; elytra black or brown, with two arched concentric horseshoe-shaped lines, crossing the suture with their posterior part.

###### Materials examined.

**Asir**: Ahd Rifidh, 18°06.33'N, 42°53.82'E, 16.I.2013, BS, Al Ansi, A., 1ex.

###### Local distribution.

This is a newly recorded genus from Saudi Arabia; it was collected from Asir province.

###### World distribution.

**Asia**: IN, IQ, SY, and TR; **Europe**: AL, AR, AU, AZ, BH, CR, CZ, FR, GB, GE, GG, GR, HU, IT, NL, RO, SK, ST, SZ, UK, and YU; **North Africa**: CI, and MR; **AFR** and **NAR** (Kovář 2007); new record for SA.

#### *Nephus* Mulsant, 1846

##### Nephus (Bipunctatus) conjunctus

Taxon classificationAnimaliaColeopteraCoccinellidae

(Wollaston, 1870)

B4A57B47-F3D5-532F-8419-958E988B6542

Scymnus conjunctus Wollaston, 1870: 248.

###### Remark.

Despite extensive visits throughout the kingdom, this species was not collected during this study period. This species is a predator of aphids (Raimundo et al. 2007).

###### Local distribution.

Asir (Fürsch 1979).

###### World distribution.

**E**: IT, PT, and SP; **N**: AG, CI, EG, MO, and TU; **A**: PAL, SA, and TR; **AFR** (Kovář 2007); AE (Raimundo et al. 2007).

##### Nephus (Bipunctatus) nigricans

Taxon classificationAnimaliaColeopteraCoccinellidae

(Weise, 1879)*

E511993F-6554-5742-9ED2-FBD31ADECE5F

Figure 5

Scymnus (Nephus) bipunctatus nigricans Weise, 1879: 154.

###### Diagnosis.

Body is oval, shining, convex; antennae are 9-segmented; pubescent body, overall black species; each elytron has one large reddish spot in the posterior half.

**Figure 5.**
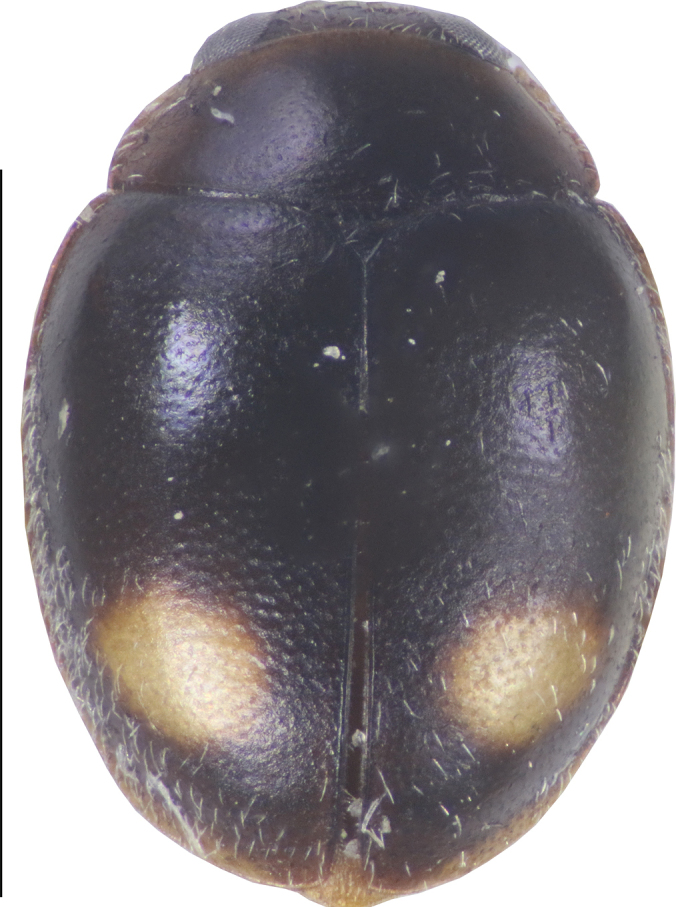
Dorsal view of *Nephus nigricans*. Scale bar: 1 mm.

###### Materials examined.

**Asir**: Al Majardah Wadi Baqrah Amer Dam, 18°47.57'N, 42°01.12'E, 433 m, 31. V.2012, BS, Al Ansi, A., 1♂; **Baha**: Bani Farwah, 20°02.13'N, 41°29.37'E, 2065 m, 25.IV.2013, BS, Al Ansi et al., 1♀; Baidah Dam, 20°11.88'N, 41°24.06'E, 1880 m, 24.IV.2013, BS, Al Ansi et al., 1♂1♀.

###### Local distribution.

Members of this species were collected from Asir and Baha provinces.

###### World distribution.

**Asia**: TR and YE (Raimundo and van Harten 2000), new country record for Saudi Arabia; **Europe**: AL, CR, FR, GR, IT, and TR (Kovář 2007), HU, SK (Haviar and Merkl 2004).

##### Nephus (Bipunctatus) ornatulus

Taxon classificationAnimaliaColeopteraCoccinellidae

Korschefsky, 1931*

7B287350-6F05-5297-9955-B67E1946EE05

Figure 6

Nephus (Bipunctatus) ornatulus Korscefsky, 1931: 152.

###### Diagnosis.

Body is elongate oval, covered with whitish pubescence; elytra are generally black, each with two yellowish spots: the anterior one is small or inconspicuous, sometimes invisible, located on the disc, and the posterior one is subapical, large, circular, or transverse.

**Figure 6.**
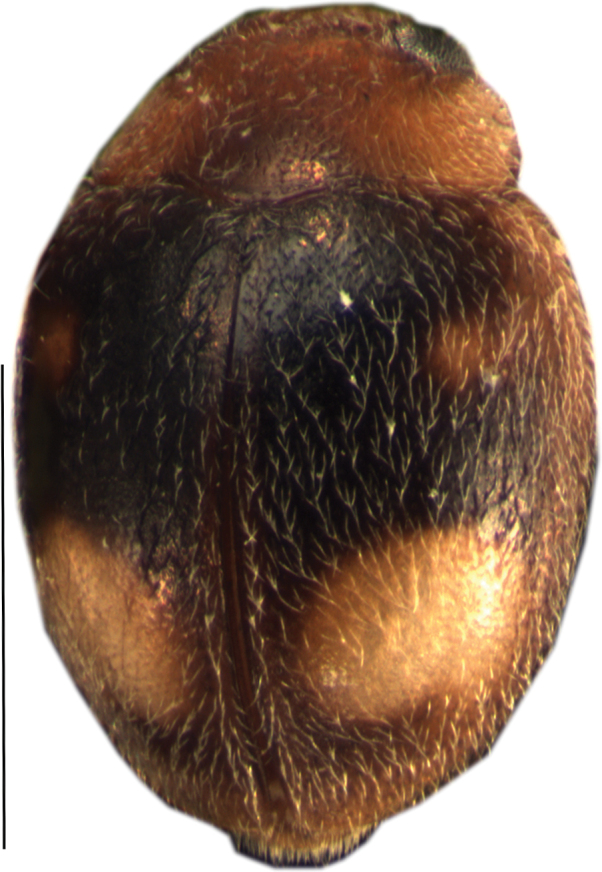
Dorsal view of *Nephus ornatulus*. Scale bar: 1 mm.

###### Materials examined.

**Asir**: Wadi bin Hashbal, 18°35.44'N, 42°39.01'E, 1892 m, 26.IV.2011, SN, Al Ansi et al., 1♀; Al Magardah Wadi Yabah, 19°16.27'N, 41°48.46'E, 411, 2.VI.2012, BS, Al Ansi, A., 1♂.

###### Local distribution.

Members of this species were collected from Asir; it was not previously known in Saudi Arabia.

###### World distribution.

**Asia**: YE, new country record for Saudi Arabia; **Africa**: Mauritania, Sierra Leone, Cameroon, and Senegal (Raimundo et al. 2006).

##### Nephus (Bipunctatus) wittmeri

Taxon classificationAnimaliaColeopteraCoccinellidae

Fürsch, 1979

CD52F63D-DFDB-5C45-8548-78A2EA12C139

Nephus wittmeri Fürsch, 1979: 245.

###### Remark.

This is a rare species reported only from the KSA and in a few Afrotropical countries.

###### Materials examined.

**Asir**: Al Magardah Wadi Yabah, 19°16.27'N, 41°48.46'E, 411, 2.VI.2012, BS, Al Ansi, A., 1♂; **Baha**: Thee Ain, 19°55.78'N, 41°26.60'E, 741 m, 11. V.2011, SN, Fadl et al., 1ex; **Riyadh**: Nursery, 29.IX.2007, SN, Al Dhafer et al., 1ex; Rawdhet Khoraim, 25°22.98'N, 47°16.71'E, 559 m, 18.II.2012, SN, 4exs, on *R. steica*.

###### Local distribution.

It was reported from Riyadh by Fürsch (1979); in the present study, it was collected from Asir and Baha. It was also reported from Riyadh by Abdel-Dayem et al. (2017).

###### World distribution.

**A**: SA; **AFR** (Kovář 2007).

##### Nephus (Geminosipho) arcuatus

Taxon classificationAnimaliaColeopteraCoccinellidae

Kapur, 1959

D623C828-4C84-5B2D-BC66-32F6CD2FD7CF

Nephus (Geminosipho) arcuatus Kapur, 1959: 287.

###### Remark.

This species is found in regions of the Middle East and Africa with an Afrotropical distribution and found in natural habitats. It was generally collected in the months of May and June.

###### Materials examined.

**Asir**: Al Magardah, Wadi Yabah, 19°16.27'N, 41°48.46'E, 411, 2.VI.2012, BS, Al Ansi, A., 2exs; Al Magardah, Wadi Khat, 19°05.37'N, 41°58.37'E, 31. V.2012, BS, Al Ansi, A., 3exs; **Baha**: Thee Ain, 19°55.78'N, 41°26.60'E, 741 m, 3.VI.2012, BS, Al Ansi, A., 1♂5exs; **Jizan**: King Faisal Sport City, 16°48.23'N, 42°59.49'E, 11m, 20.V.2012, BS, Al Ansi, A., 1♀; Hurub, Wadi Hurub, 17°43.12'N, 42°57.88'E, 398 m, 24.V.2012, BS, Al Ansi, A., 1♂; **Najran**: Hubuna, Al Dhaiqah, 17°50.71'N, 44°15.83'E, 1228 m, 14, I.2013, BS, Al Ansi et al., 1ex.

###### Local distribution.

Specimens of this species were collected from Asir, Baha, Jizan and Najran. It was previously reported by Fürsch (1979) from Asir.

###### World distribution.

**Asia**: IN, SA, and YE; **AFR** (Kovář 2007); AE (Raimundo et al. 2007).

##### Nephus (Geminosipho) fenestratus

Taxon classificationAnimaliaColeopteraCoccinellidae

(Sahlberg, 1913)

2A16798F-4EEA-54BC-910C-3ED9C3FCC90B

Scymnus fenestratus Sahlberg, 1913: 83.

###### Remark.

This species was not found during the present study, although it was previously recorded from Riyadh and Jizan provinces.

###### Local distribution.

Riyadh and Jizan (Fürsch 1979).

###### World distribution.

**Asia**: SA, SY, and YE (Raimundo and van Harten 2000); AE, CY, IN, PAL, JO, and AE (Raimundo et al. 2007); **North Africa**: EG; **AFR** (Kovář 2007).

##### Nephus (Nephus) crucifer

Taxon classificationAnimaliaColeopteraCoccinellidae

Fleischer, 1900

85AE716E-4844-5985-ABA1-70231C2ED94A

###### Local distribution.

This species was found in Asir, Baha, and Makkah and was also reported by Fürsch (1979) from Asir province.

###### Materials examined.

**Asir**: Ahd Rifidh, 18°06.33'N, 42°53.82'E, 16.I.2013, BS, Al Ansi et al., 3♂3♀; **Baha**: Al Mikhwah, 19°49.44'N, 41°22.85'E, 430 m, 18.V.2010, SN, Al Dhafer et al., 1♂; Wadi Al Zaraeb, 20°03.60'N, 41°23.19'E, 2123 m, 15.V.2010, BS, Sharaf, M., 1♂; Sad Medhas, 20°13.26'N, 41°16.53'E, 1781 m, 9.III.2012, SN, Al Dhafer et al., 1♂; Wadi Turubah, 20°14.37'N, 41°15.23'E, 9.III.2012, SN, Al Dhafer et al., 1♀; Shabreqah station, 20°08.03'N, 41°23.60'E, 2267 m, 24.IV.2013, BS, Al Ansi et al., 1♂1♀; King Saud Rd., 20°00.28'N, 41°27.91'E, 2132 m, 25.IV.2013, BS, Al Ansi et al., 2♂2♀; Shuaib Wadi Turubah, 20°14.37'N, 41°15.23'E, 24.IV.2013, BS, Al Ansi et al., 1♂; Midan Al Shuhadaa, 20°00.00'N, 41°27.99'E, 2124 m, 25.IV.2013, BS, Al Ansi et al., 1♀; Rahwan 10 km outside of Baha, 20°04.03'N, 41°27.01'E, 2272 m, 24.IV.2013, BS, Al Ansi et al., 1♀; Baidah Dam, 20°11.88'N, 41°24.06'E, 1880 m, 24.IV.2013, BS, Al Ansi et al., 1♂; Al Lishtah Village 20 km outside of Baha, 26.IV.2013, BS, Al Ansi et al., 1♂; Makkah: Baha-Taif Rd. Bani Malik Wadi Rakhmat, 20°42.15'N, 40°59.46'E, 1616 m, 20°42.15'N, 40°59.46'E, 1616 m, 4.VI.2012, BS, Al Ansi, A., 1♀.

###### World distribution.

**Asia**: IN, SA, TD, UZ, and KZ; **AFR** (Kovář 2007).

##### Nephus (Sidis) hiekei

Taxon classificationAnimaliaColeopteraCoccinellidae

(Fürsch, 1965)

B0668772-3C86-5AAC-B9D8-930F6D5408B4

Scymnus hiekei Fürsch, 1965: 201.

###### Remark.

This species is known as a predator of the mealybug *Planococcus citri* (Risso, 1813 (Fürsch 1970, Abu-Thuraya 1982, Raimundo and van Harten 2000).

###### Materials examined.

**Riyadh**: Ad Diriyah, 24°40'N, 46°35'E, 17.XI.2011, SN, Al Omar, A., 1ex; 15.IX.2011, SN, Al Omar, A., 1ex; Al Ammariah, 24°49'N, 46°26'E, 24.XI.2009, SN, Sharaf, M., 1ex.

###### Local distribution.

It is restricted to Riyadh province and previously reported from Riyadh by Fürsch (1979) and Abu-Thuraya (1982) and listed by Martin (1972) and Talhouk (1982). In this study, it was collected from Riyadh.

###### World distribution.

**Asia**: AE, IN, IQ, PAL, LE, SA, and TR; **Europe**: IT, GR, and SP; **North Africa**: AG, EG, MO, and TU (Kovář 2007).

##### Nephus (Sidis) levaillanti

Taxon classificationAnimaliaColeopteraCoccinellidae

(Mulsant, 1850)

84EDCD5D-666E-52F8-87B0-A1262CA4DA5E

Scymnus levaillanti Mulsant, 1850: 964.

###### Remark.

The specimens were collected by PT on the branches of *Acacia gerrardii* and under the shrub *Z. nummularia*. Some specimens were also collected under the canopies of *Acacia ehrenbergiana* during April and July. This species is a predator on *Aphis gossypii* Glover, 1877 (Fürsch 1970) and *A. orientalis* (Abu-Thuraya 1982).

###### Materials examined.

**Riyadh**: Rawdhet Khoraim, 25°22.98'N, 47°16.71'E, 559 m, 28.IV.2012, PT, 1♀, under *A. ehrenbergiana*; 28.IV.2012, PT, 1♂, under *A. gerrardii*; 1.VII.2012, BS, 1♀, on *A. gerrardii*; 14.IV.2012, BS, 1ex, on *Z. nummularia*.

###### Local distribution.

Specimens were collected from Riyadh (Abdel-Dayem et al. 2017) and also previously was reported from Riyadh and Eastern provinces by Abu-Thuraya (1982) and listed by Martin (1972).

###### World distribution.

**Asia**: SA (Raimundo and van Harten 2000); AF, FE, IN, PAL, JA, JO, LE, PA, SHA, and TAI; **Europe**: GR and IT; **North Africa**: EG; **AFR** and **ORR** (Kovář 2007).

#### *Scymnus* Kugelann, 1794

##### Scymnus (Pullus) agrumi

Taxon classificationAnimaliaColeopteraCoccinellidae

Fürsch, 1970

9EF49D4C-820D-532B-A889-13BFA3AD1C94

Scymnus (Pullus) agrumi Fürsch, 1970: 109.

###### Remark.

This is a very rare species that is endemic to Saudi Arabia and found to be a predator of the mealybug *P. citri* (Fürsch 1970, Martin 1972; Abu-Thuraya 1982).

###### Material examined.

**Eastern region**: Qatif, 26°45'N, 49°58'E, 1969, Fürsch, ANMA, 1ex, paratype.

###### Local distribution.

It was described from the Eastern province of KSA by Fürsch (1970), reported by Abu-Thuraya (1982) also from Eastern Province, and listed by Martin (1972).

###### World distribution.

**Asia**: Endemic to SA (Fürsch 1970; Kovář 2007).

##### Scymnus (Pullus) arabicus

Taxon classificationAnimaliaColeopteraCoccinellidae

Fürsch, 1989

827AA0DB-5C6B-5F6C-A428-E4C825936879

Scymnus arabicus Fürsch, 1989: 116.

###### Remark.

This is also a rare species that has not been collected since its description by Fürsch (1989). It is endemic to Saudi Arabia.

###### Local distribution.

It was found in Asir province (Fürsch 1989).

###### World distribution.

**Asia**: Endemic to SA (Fürsch 1989; Kovář 2007).

##### Scymnus (Pullus) auritus

Taxon classificationAnimaliaColeopteraCoccinellidae

Thunberg, 1795

368B5704-2172-57EB-A668-78F857BDBAB8

Scymnus (Pullus) auritus Thunberg, 1795: 105.

###### Remark.

Presence of this species was not confirmed, except in the catalogue of Kovář (2007) with insufficient details regarding its distribution in Saudi Arabia. However, it is well distributed around the world.

###### World distribution.

**Asia**: ANH, CY, ES, FE, FUJ, GUA, IN, SA, SCH, SY, TR, and WS; **Europe**: AL, AU, BH, BU, BY, CR, CT, CZ, DE, EN, FI, FR, GB, GE, GG, GR, HU, LA, LT, LU, MC, MD, NR, NT, PL, PT, RO, SK, SL, SP, ST, SV, SZ, TR, UK, and YU (Kovář 2007).

##### Scymnus (Pullus) ebneri

Taxon classificationAnimaliaColeopteraCoccinellidae

Weise, 1926

3D7753EC-2FB7-513A-AD65-1BAE7F5D5371

Scymnus ebneri Weise, 1926: 228.

###### Remark.

This is also a rare species and known as a predator of aphids (Abu-Thuraya 1982; Talhouk 1982).

###### Material examined.

**Eastern province**: Qatif, 26°45'N, 49°58'E, 5.VIII.1970, det. R. D. Pope, 1971, ANMA, 1ex, on alfalfa.

###### Local distribution.

It was reported from Riyadh by Abu-Thuraya (1982) and listed by Martin (1972) and Talhouk (1982).

###### World distribution.

**Asia**: AE, IN, and SA (Abu-Thuraya 1982). **North Africa**: EG; **AFR** (Kovář 2007).

##### Scymnus (Pullus) latemaculatus

Taxon classificationAnimaliaColeopteraCoccinellidae

Motschulsky, 1858

79F6C05E-192B-53AE-9731-7868EB78A9D2

Scymnus (Pullus) latemaculatus Motschulsky, 1858: 121.

###### Remark.

Specimens of this species could not be examined due to the unavailability of material.

###### Local distribution.

This species was reported from Riyadh by Fürsch (1979).

###### World distribution.

**Asia**: AF, FUJ, GUA, GUX, IN, PA, TAI, ZHE, and SA; **North Africa**: LB; **ORR** (Kovář 2007); AE (Raimundo et al. 2007).

##### Scymnus (Pullus) luxorensis

Taxon classificationAnimaliaColeopteraCoccinellidae

Fürsch, 1989

981506E8-C043-59E1-9E1F-4193CBE25F43

Scymnus (Pullus) luxorensis Fürsch, 1989: 116.

###### Remark.

This is a rare species with limited geographical distribution. It was collected from *L. shawii* using a beating sheet in October; it is also not common in its type locality range.

###### Material examined.

**Riyadh**: Rawdhet Khoraim, 25°22.98'N, 47°16.71'E, 559 m, 30.X.2011, BS, Al Ansi, A., 1♂, on *L. shawii*.

###### Local distribution.

It was described from Riyadh by Fürsch (1989) and also collected again from Riyadh by Abdel-Dayem et al. (2017).

###### World distribution.

**Asia**: It was described from SA (Fürsch 1989) and also known in **North Africa**: EG (Kovář 2007).

##### Scymnus (Pullus) subvillosus

Taxon classificationAnimaliaColeopteraCoccinellidae

(Goeze, 1777)

2DAF6FFF-B404-50FE-82BA-4AA7A0677830

Coccinella subvillosus Goeze, 1777: 247.

###### Remark.

This is a very common species found throughout the year in SA and also widely distributed throughout the most world countries. This species was primarily collected from the habitats of *A. ehrenbergiana*, *A. gerrardii*, *C. procera*, *L. shawii*, *R. stricta*, and *Z. nummularia*.

###### Material examined.

**Asir**: Al Majardah Wadi Al Talalie, 19°05.19'N, 41°47.78'E, 286 m, 1.VI.2012, BS, Al Ansi, A., 1ex; Al Majardah Wadi Khat, 19°05.37'N, 41°58.37'E, 13.III.2012, BS, Abdel-Dayem et al., 3exs; 31.V.2012, BS, Al Ansi, A., 4exs; Al Majardah Wadi Qanunah, 19°24.67'N, 41°36.39'E, 348 m, 11.III.2012, SN, Al Dhafer et al., 1ex; 11.III.2012, LT, Al Dhafer et al., 1ex; Al Majardah, Wadi Yabah, 19°16.27'N, 41°48.46'E, 411, 2.VI.2012, SN, Al Ansi, A., 2♀30exs; 2.VI.2012, BS, Al Ansi, A., 1♂4♀37exs; 12.III.2012, SN, Al Dhafer et al., 1ex; Ahd Rifidh, 18°06.33'N, 42°53.82'E, 16.I.2013, SN, Al Ansi et al., 2exs; **Baha**: Thee Ain, 19°55.78'N, 41°26.60'E, 741 m, 11.V.2011, SN, Fadl et al., 1ex; 10.III.2012, SN, Al Dhafer et al., 1♂,2exs; 3.VI.2012, BS, Al Ansi, A., 1ex; **Jizan**: Al Aydabi Jaorat Aiban, 17°25.53'N, 43°03.50'E, 343 m, 17°25.53'N, 43°03.50'E, 343 m, 23.V.2012, BS, Al Ansi, A., 2exs; **Najran**: Rijla Wadi Najran, 17°31.56'N, 44°13.65'E, 1257 m, 15.I.2013, BS, Al Ansi et al., 15exs; Wadi Shuaib Barran, 17°28.94'N, 44°05.52'E, 1325 m, 16.I.2013, BS, Al Ansi et al., 7exs; Hubuna Al Dhaiqah, 17°50.71'N, 44°15.83'E, 1228 m, 14.I.2013, BS, Al Ansi et al., 11exs; Hubuna Lahumah, 17°50.47'N, 44°16.82'E, 1212 m, 14.I.2013, BS, Al Ansi et al., 75exs; **Riyadh**: Al Muzahimiyah Al Khararah, 24°24.21'N, 46°14.40'E, 30.III.2011, SN, Al Dryhim et al., 9exs; 17.IV.2012, LT, Al Dhafer et al., 3exs; Rawdhet Khoraim, 25°22.98'N, 47°16.71'E, 559 m, 182 exs collected by SN and B on branches of and by PT under canopy of *A. ehrenbergiana*, *A. gerrardii*, *C. procera*, *L. shawii*, *R. stricta*, *Z. nummularia*; ; also by LT; XII.2011; I, III-VII, IX, XI.2012; I-IX.2013; 11.IV.2011, SN, Al Dryhim et al., 1ex; 11.IV.2011, SN, Al Dryhim et al., 1ex; Hutet Bani Tamim Al Hareeq Rd., 23°32.03'N, 46°47.33'E, 584 m, 8.V.2012, LT, Al Ansi, A., 1ex; Hutet Bani Tamim Ibex Reserve National Park, 23°28.10'N, 46°36.35'E, 28.XII.2007, SN, Al Dryhim et al., 1ex; 19.IV.2008, SN, 4exs; 19.V.2007, SN, 2exs; 21.III.2008, ST, 1ex; 21.III.2008, SN, 1ex; 4.IV.2008, SN, 2exs; Wadi Ad Dawasir, 20°25.83'N, 44°56.53'E, 641 m, 17.I.2013, BS, Al Ansi et al., 1ex; Wadi Namar, 24°32.18'N, 46°34.60'E, 29.II.2012, HP, Al Ansi, A., 1ex; Dirab, 23°30'N, 46°51'E, 26.IV.2010, SN, Rashid, O., 1ex; 14.III.2010, ST, Al Dhafer et al., 1ex; 25.IV.2010, ST, Al Dhafer et al.,1ex; 28.III.2010, ST, Al Dhafer et al.,2exs; 20.XII.2009, ST, Al Dhafer et al.,1ex; Riyadh Nursery, 16.V.2007, SN, Al Dhafer et al., 1ex; 29.IX.2007, SN, Al Dhafer et al.,1ex; Al Waseel, 24°48.40'N, 46°30.42'E, 20.X .2011, SN, Al Rashide, H., 1ex; Ad Diriyah Education Farm, 24°40'N, 46°35'E, 21.X.2009, SN, Salah, A., 1ex; Al Ahsa, 22°17.53'N, 50°40.46'E, 28.XII.1982, Al Shuaaei, M., ANMA, 1ex.

###### Local distribution.

It was collected from different localities of Asir, Baha, Eastern Province, Jizan, Najran, and Riyadh provinces and previously reported by Fürsch (1979) and Abdel-Dayem et al. (2017).

###### World distribution.

**Asia**: AE, AF, CY, IN, IQ, PAL, JO, KI, KU, KZ, LE, PA, QA, SA, SI, SY, TD, TR, UZ, and YE; **Europe**: AB, AL, AN, AR, AU, AZ, BH, BU, CR, CZ, FR, GE, GG, GR, HU, IT, MC, PT, RO, SK, SL, SP, ST, SZ, TR, UK, and YU; **North Africa**: AE, AG, CI, EG, LB, MO, MR, and TU; **AFR** (Kovář 2007).

##### Scymnus (Pullus) syriacus

Taxon classificationAnimaliaColeopteraCoccinellidae

(Marsuel, 1868)

52DB093F-EF06-50CC-A3EE-162F598A5F89

Nephus syriacus Marsuel, 1868: 216.

###### Remark.

This is a widely distributed species throughout the year in SA and was collected from the habitats of *A. ehrenbergiana*, *A. gerrardii*, *C. procera*, *L. shawii*, *R. stricta*, and *Z. nummularia*. It is a predator on *Aphis fabae* Scopoli, 1840 (Talhouk 1982; Fürsch 1989) and whiteflies and aphids (Martin 1972).

###### Material examined.

**Riyadh**: Hutet Bani Tamim Ibex Reserve National Park, 23°28.10'N, 46°36.35'E, 4.IV.2008, SN, Al Dryhim et al., 2♂30exs; 21.III.2008, SN, 2♀3exs; 28.VII.2007, SN, 1ex; 19.V.2007, SN, 2exs; 19.IV.2008, SN, 1ex; 7.V.2012, LT, Al Ansi, A., 1ex; 28.V.2010, SN, Al Dhafer et al., 2exs; Hutet Bani Tamim-Al Hareeq Rd., 23°32.03'N, 46°47.33'E, 584 m, 8.V.2012, LT, Al Ansi,A., 1♀3exs; Al Hareeq-Ar Rayn Rd., 23°33.88'N, 46°22.53'E, 737 m, 8.V.2012, LT, Al Ansi, A., 1♂; Hutet Bani Tamim, 23°27.26'N, 46°41.13'E, 19.IV.2008, SN, Al Dhafer, H., 7exs; 7.V.2012, HP, Al Ansi, A., 1ex; Rawdhet Khoraim, 25°22.98'N, 47°16.71'E, 559 m, 98exs were collected by SN and B on branches of *A. ehrenbergiana*, *A. gerrardii*, *C. procera*, *L. shawii*, *R. stricta* and *Z. nummularia*; and HP under canopy of the previous plants; also by LT; through X, XII.2011; II-VI, X-XII.2012; III, IV, VI, VII.2013; 11.IV.2011, SN, Al Dryhim et al., 1♀; 11.IV.2011, SN, Al Dryhim et al., 1ex; 16.X.2011, LT, 1ex; 26.V.2012, BS, 1♂1ex; 26.V.2012, LT, 1♂1ex; 18.II.2012, SN, 1ex; 26.V.2012, BS, 1♂1ex; 15.V.2012, SN, 1ex; 14.V.2012, BS, 1ex; 14.V.2012, SU, 2exs; 14.V.2012, BS, 1ex; Wadi Namar, 24°32.18'N, 46°34.60'E, 29.II.2012, BS, Al Ansi et al., 1ex; Al Kharj, 20°24'N, 46°29'E, 18.XI.2009, SN, Al Dryhim et al., 2exs; Wadi Ad Dawasir, 20°25.83'N, 44°56.53'E, 641 m, 17.I.2013, BS, Al Ansi et al., 1ex; Al Muzahimiyah Al Khararah, 24°24.21'N, 46°14.40'E, 30.III.2011, SN, Al Dryhim et al., 1♂1♀4exs; **Najran**: Hubuna Al Dhaiqah, 17°50.71'N, 44°15.83'E, 1228 m, 14.I.2013, BS, Al Ansi et al., 2exs; Rijla Wadi Najran, 17°31.56'N, 44°13.65'E, 1257 m, 15.I.2013, BS, 2exs; Hubuna Lahumah, 17°50.47'N, 44°16.82'E, 1212 m, 14.I.2013, BS, 4exs.

###### Local distribution.

Specimens of this species were collected from Eastern, Najran, and Riyadh provinces and have been previously reported from Eastern Province by Talhouk (1982) and Fürsch (1989), from Eastern province and Baha by El-Hawagry et al. (2013), and from Riyadh by Abdel-Dayem et al. (2017); it was listed by Martin (1972).

###### World distribution.

**Asia**: SA (Fürsch 1989); **Asia**: CY, IN, IQ, PAL, JO, LE, SI, and SY; **North Africa**: EG; (Kovář 2007).

##### Scymnus (Pullus) yemenensis

Taxon classificationAnimaliaColeopteraCoccinellidae

(Kapur, 1959)

55F4B6AF-F4B9-52F3-A7BC-B614B8277CF3

Pullus yemenensis Kapur, 1959: 281.

###### Remark.

This species is distributed only in the Arabian Peninsula and is generally found in the natural habitats of *A. ehrenbergiana* and *C. procera* in the vicinity of hemipterous insects.

###### Material examined.

**Asir**: Abha, Habalah, 18°02.05'N, 42°51.49'E, 25.IV.201 SN, Al Ansi et al., 5♂1♀1ex; Ahd Rifidh, 18°06.33'N, 42°53.82'E, 16.I.2013, BS, Al Ansi et al., 7♂3♀; **Baha**: King Saud Rd., 20°00.28'N, 41°27.91'E, 2132 m, 25.IV.2013, BS, Al Ansi et al., 4♂1♀; Al Leishtah Village 20 km outside of Baha, 20°12.16'N, 41°22.21'E, 2231 m, 26.IV.2013, BS, 1♂; Baidah Dam, 20°11.88'N, 41°24.06'E, 1880 m, 24.IV.2013, BS, 2♂1♀; Midan Al Shuhadaa, 20°00.00'N, 41°27.99'E, 2124 m, 25.IV.2013, BS, 2♂5♀; Wadi Turubah, 20°14.37'N, 41°15.23'E, 26.IV.2013, BS, 2♂2♀; Rahwan, 10 km outside of Baha, 20°04.03'N, 41°27.01'E, 2272 m, 24.IV.2013, BS, 1♀; Thee Ain, 19°55.78'N, 41°26.60'E, 741 m, 25.IV.2013, BS, 1♀; Shabreqah Station, 20°08.03'N, 41°23.60'E, 2267 m, 24.IV.2013, BS, 4♀; Wadi Turubah, 20°14.37'N, 41°15.23'E, 14.V.2011, SN, Al Dhafer, H., Fadl, H., Sharaf, M., 1♂; 14.X.2010, SN, Al Dhafer et al., 1♂1♀; Al Mandaq Wadi Turubah, 20°14.37'N, 41°15.23'E, 3.VI.2012, BS, Al Ansi, A., 1♂; **Najran**: Al Mofejah, 17°27.25'N, 44°03.48'E, 1263 m, 16.I.2013, BS, Al Ansi et al., 1♂; Rijla Wadi Najran, 17°31.56'N, 44°13.65'E, 1257 m, 15.I.2013, BS, 2♂1♀; Hubuna Lahuma, 14.I.2013, BS, 3♂; Najran- Dhahran Al Janub Rd., 17°36.971'N, 43°32.97'E, 2132 m, 16.I.2013, BS, 1♀; **Riyadh**: Hawtet Bani Tameem- Al Hariq Rd. 15 km, 8.V.2012, LT, Al Ansi, A., 1♂; Wadi Namar, 24°32.18'N, 46°34.60'E, 29.II.2012, HP, Al Ansi, A., 1♂; Dirab, 23°30'N, 46°51'E, 30.XII.2009, SN, Sofan, A., 1♀; Rawdhet Khoraim, 25°22.98'N, 47°16.71'E, 559 m, 14.IV.2012, BS, 1♂ on *A. ehrenbergiana*; 28.IV.2012, LT, 1♀; 14.IV.2012, SN, 1♂; 13.X.2012, LT, 1ex, on *C. procera*; 13.X.2012, SN, 5exs, on *C. procera*.

###### Local distribution.

It was collected from Asir, Baha, Najran, and Riyadh and was previously reported by Fürsch (1979) from Asir and Riyadh and from Riyadh by Fürsch (1989) and also by Abdel-Dayem et al. (2017).

###### World distribution.

**Asia**: OM, SA, and YE (Kovář 2007); AE (Raimundo et al. 2007).

##### Scymnus (Scymnus) interruptus

Taxon classificationAnimaliaColeopteraCoccinellidae

(Goeze, 1777)

CC187A04-5A20-599D-853A-574B56F07AF3

Coccinella interrupta Goeze, 1777: 247.

###### Remark.

This species is widely distributed in SA and commonly found in natural habitats.

###### Materials examined.

**Asir**: Muhail Wadi Hali, 18°30.12'N, 42°02.21'E, 440 m, 11.II.2016, SU, Al Ansi, A., 30exs; Maraba Wadi Reem, 17°52.55'N, 42°16.66'E, 136 m, 9.II.2016, SU, Al Ansi, A., 41exs; Maraba Wadi Ramlan, 17°47.18'N, 42°22.95'E, 180 m, 10.II.2016, SU, Al Ansi, A., 19exs; Maraba Wadi Itwad, 17°48.25'N, 42°21.64'E, 149 m,8.II.2016, SU, Al Ansi, A., 29exs; Rijal Alma Wadi Sabin, 17°48.25'N, 42°21.64'E, 194 m, 10.II.2016, SU, Al Ansi, A., 12exs; Wadi Maraba, 18°10.29'N, 42°22.19'E, 1150 m,16.IV.2016, LT, Abdel-Dayem et al., 1ex; Raghdan, 20°34.25'N, 41°45.11'E, 10.IV.2016, SU, Al Ansi, A., 4exs; Abha, Raydah, 18°12.10'N, 42°24.54'E, 2578 m, 16.IV.2016, SN, Soliman, A., 1ex; Thalooth Al Mandhar Wadi Baqrah, 18°47.57'N, 42°01.12'E, 433 m, 12.X.2013, SN, Khan, S., 1ex; Al Mikhwah-Muhail Rd. Hadaba Hamra, 19°13.19'N, 41°46.67'E, 374 m, 15.IV.2016, SN, Al Ansi, A., 1ex; **Baha**: Al Mikhwah Thee Ain, 19°55.78'N, 41°26.60'E, 741 m, 18.V.2010, SN, Al Dhafer et al., 1ex; 12.IV.2016, SU, Al Ansi, A., 27exs; Wadi Turubah, 20°14.37'N, 41°15.23'E, 10.V.2011, SN, Fadl et al., 1ex; Thee Ain, 19°55.78'N, 41°26.60'E, 741 m, 13.X.2010, SN, Al Dhafer et al., 1ex; 7.III.2013, SN, Al Harbi et al., 1ex; 7.III.2013, BS, Al Harbi et al., 1ex; **Jizan**: Sabya-Hurub Rd., 17°16.94'N, 42°17.54'E, 97 m, 25.V.2012, BS, Al Ansi, A., 1♂1♀1ex; Jizan-Abu Arish Rd., 17°04.25'N, 42°47.05'E, 24.II.2015, SN, Al Harbi, M., 2exs; LT, 3exs; HP, 1ex; **Najran**: Rijla Wadi Najran, 17°31.56'N, 44°13.65'E, 1257 m, 15.I.2013, BS, Al Ansi et al., 3exs.

###### Local distribution.

It was collected from Asir, Baha, Jizan, and Najran and was previously reported by Fürsch (1979) in the taxonomic key.

###### World distribution.

**Asia**: IQ, PAL, JO, KZ, MG, TR, SY, and YE; **Europe**: AB, AL, AR, AU, BE, BU, BY, CR, CZ, FR, GE, GG, GR, HU, IT, UZ, WS, XIN, PT, RO, SK, SP, SZ, TR, and UK; **North Africa**: AG, EG, LB, MO, MR, and TU (Kovář 2007).

##### Scymnus (Scymnus) nubilus

Taxon classificationAnimaliaColeopteraCoccinellidae

Mulsant, 1850

2FA6FF88-0E6F-59AD-BE4D-9869D2B61E1A

Scymnus nubilus Mulsant, 1850: 972.

###### Remark.

This species is found in both natural habitats and agroecosystems and collected throughout the year in SA. It is a well-known predator of the sugarcane whitefly, *Aleurolobus barodensis* (Maskell, 1896) (Hodek and Honek 2009) and aphids (Raimundo et al. 2007).

###### Material examined.

**Asir**: Wadi Abha, 18°22.03'N, 42°50.82'E, 1990 m, 28.IV.2011, SN, Al Ansi et al., 1ex; Wadi Ghanuna, 19°24.67'N, 41°36.39'E, 348 m, 11.V.2011, SN, Fadl et al., 1ex; Al Majaridah, Wadi Yabah, 19°16.27'N, 41°48.46'E, 411, 2.VI.2012, SN, Al Ansi, A, 1♂4exs.; 2.VI.2012, BS, Al Ansi, A., 4exs; Al Majaridah, Wadi Al Talalie, 19°05.19'N, 41°47.78'E, 286 m, 1.VI.2012, LT, Al Ansi et al., 1ex; 1.VI.2012, SN, Al Ansi, A., 2exs; Khamis Mushait, Wadi Bisha, 18°20.01'N, 42°42.13'E, 1990 m 27.IV.2011, SN, Al Ansi, A., 3exs; Ahd Rifidh, 18°06.33'N, 42°53.82'E, 16.I.2013, BS, Al Ansi, A., 1ex; 16.I.2013, SN, Al Ansi, A., 4exs; Thalooth Al Mandhar Wadi Baqrah, 18°47.57'N, 42°01.12'E, 433 m, 4.XI.2013, SN, Al Dhafer et al., 1ex; LT, 1ex; 12.X.2013, HP, El Torkey, A., 1ex; **Baha**: Qilwah-Adhom, Wadi Al Arg, 20°29.98'N, 40°48.95'E, 429 m, 9.XI.2012, BS, Fadl, H., 1ex; Thee Ain, 19°55.78'N, 41°26.60'E, 741 m, 15.V.2011, SN, Fadl et al., 3exs; 3.VI.2012, SN, Al Ansi, A., 2exs; Ghabat Raghadan, 20°34.25'N, 41°45.11'E, 13.V.2011, SN, Fadl et al., 2exs; Wadi Turubah, 20°14.37'N, 41°15.23'E, 14.X.2010, SN, Fadl et al., 1ex; Wadi Bawah, 20°43.93'N, 41°16.82'E, 1347 m, 8.XI.2012, BS, Fadl, H., 2exs; 8.XI.2012, HP, Abdel-Dayem, M., 1ex; Wadi Turubah, 20°14.37'N, 41°15.23'E, 14.V.2011, SN, Fadl et al., 1ex; 3.VI.2012, BS, Al Ansi, A., 1♀5exs; 3.VI.2012, SN, Al Ansi, A., 1♂; Thee Ain, 19°55.78'N, 41°26.60'E, 741 m, 13.X.2010, SN, Fadl et al., 1ex; 18.V.2010, SN, Al Dhafer et al., 3exs; 18.V.2010, BS, Sharaf, M., 1ex; Wadi Milan, 19°50.79'N, 41°21.01'E, 481 m, 7.III.2013, SN, Al Harbi et al., 14exs; Bani Farwah, 20°02.13'N, 41°29.37'E, 2065 m, 25.IV.2013, BS, Al Ansi et al., 1ex; Rahwan, 10 km outside of Baha, 20°04.03'N, 41°27.01'E, 2272 m, 24.IV.2013, BS, 1ex; King Saud Rd., 20°00.28'N, 41°27.91'E, 2132 m, 25.IV.2013, BS, 11exs; **Eastern Province**: An Nuayriyah, 27°25.26'N, 48°27.20'E, 67 m, 2.III.2011, SN, 2exs; **Jizan**: Al Aydabi, 17°21.67'N, 43°02.70'E, 272 m, 22.V.2012, SN, Al Ansi, A., 1ex; Al Aydabi, Jaorat Aiban, 17°25.53'N, 43°03.50'E, 343 m, 23.V.2012, BS, Al Ansi, A., 1ex; Al Muruj Village, 16°47.34'N, 42°45.14'E, 11.III.2010, SN, Al Dhafer, H. and El Gharabawy, A., 2exs; Al Aridah, Jizan Dam, 17°02.62'N, 42°59.36'E, 187 m, 21.V.2012, BS, Al Ansi, A., 1ex; Wadi Dhamad, 17°12.32'N, 43°01.58'E, 258 m, 11.XI.2012, LT, Abdel-Dayem, M., 1ex; **Najran**:Wadi Shuaib Barran, 17°28.94'N, 44°05.52'E, 1325 m, 16.I.2013, BS, Al Ansi, A., 10exs; Hubuna, Wadi Hubuna, 17°50.41'N, 44°02.29'E, 1309 m, 14.I.2013, SN, Al Ansi, A., 2exs; **Riyadh**: 15.IX.2007, SN, 2exs; Al Uyaynah, 24°53'N, 46°22'E, 25.VI.2011, SN, Al Ahzmy, H., 1ex; 30.XI.2010, SN, Al Ansi, A., 2exs; 24.III.2006, SN, Al Homaidi, O., 1♀; 27.IV.2011, SN, Al Jreed, Y., 1ex; Ad Diriyah, 24°40'N, 46°35'E, 7.IV.2010, SN, Hazazi, T., 1ex; 17.III, SN, 1ex; 5.II.1986, SN, 1ex; 3.X.1991, SN, 1ex; Riyadh, IX.1989, SN, 1ex; 16.X.2003, SN, Al Dhobaib, N., 1ex; Al Kharj, 20°24'N, 46°29'E, 24.III.2010, SN, Al Hashel, A., 1ex; Sajir, 24°51.23'N, 45°42.39'E, 17.XI.2011, SN, Setyaningrum, H., 1ex; Al Ammariah, 24°49'N, 46°26'E, 19.V.2009, SN, Baziad, A., 1ex; 21.X.2009, SN, Abdel-Gayed, A., 1ex; 19.XII.2002, SN, Saad, F., 1ex; Dirab, 23°30'N, 46°51'E, 2.X.1989, SN, 1ex; 18.IV.1982, SN, 1ex; 20.XI.1986, SN, Amro, 1ex; 28.III.2010, ST, Al Dhafer et al., 1ex; 7.IV.1987, SN, 1ex; 24.XI.1986, SN, 1ex; 21.IV.1987, SN, 1ex; 13.X.2010, ST, Setyaningrum et al., 1ex; 25.IV.2010, ST, Al Dhafer et al., 1ex; 26.IV.2012, SN, Al Qahtani, R., 1ex; 9.V.2012, SN, Al Qahtani, R., 1ex; Al Waseel, 24°48.40'N, 46°30.42'E, 25.III.2004, SN, Al Aati, M., 1ex; 11.V.2010, SN, Khan, M., 1ex; As Sullayyil Al Aflag Rd. 69 Km before Al Aflag, 21°22.92'N, 46°05.29'E, 653 m, 17.I.2013, BS, Al Ansi, A., 1ex; Rawdet Khoraim, 25°22.98'N, 47°16.71'E, 559 m, 18.II.2012, SN, 1ex; 30.VI.2012, SN, 1ex; 27.V.2012, LT, 1ex; 26.V.2012, MT, 1ex; 28.VII.2012, MT, 1ex; 27.VIII.2012, MT, 1ex; 27.VIII.2012, PT, 1ex; **Tabuk**: Tabuk-Dhuba Rd., 28°18.39'N, 36°02.87'E, 824 m, 15.IX.2011, SN, Al Ansi et al., 1♀40exs; Tabuk-Madinah Rd., 28°23.47'N, 36°51.96'E, 808 m, 14.IX.2011, SN, 10ex; Tabuk-Taima Rd., 27°43.51'N, 38°12.94'E, 16.IX.2011, SN, 1ex; Tabuk-Dhuba Rd., 28°18.39'N, 36°02.87'E, 824 m, 15.IX.2011, SN, 3exs.

###### Local distribution.

It is found in Asir, Baha, Eastern, Jizan, Makkah, Najran, Riyadh, and Tabuk provinces and previously reported from Asir by Fürsch (1979) as *S. levaillanti* and from Riyadh by Abdel-Dayem et al. (2017).

###### World distribution.

**Asia**: AE, AF, IN, IQ, PAL, JA (Bonin, Ryukyus), JO, KU, LE, OM, NP, PA, SA, SD, SY, TAI, TR, and UP; **Europe**: AZ, GR, IT, PT, and SP; **North Africa**: CI, EG, and MR; **AFR, AUR**, and **ORR** (Kovář 2007).

##### Scymnus (Scymnus) scapuliferus

Taxon classificationAnimaliaColeopteraCoccinellidae

Mulsant, 1850*

8CB00975-CEAC-5807-9541-A961D790C620

Figure 7

Scymnus scapuliferus Mulsant, 1850: 968.

###### Diagnosis.

Body is elongated and convex; head is pale in males and black with anterior yellow border in females. Pronotum is black except for a narrow testaceous border on the anterior margin. Elytra are black with yellowish, reddish or pale, nearly quadrangular shoulder spot along the proximal two-thirds. Specimens of this species were found mostly in natural ecosystems, although it has been reported as a predator of the cowpea aphid, *Aphis craccivora* Koch, 1854, in Nigeria (Waterhouse 1998).

**Figure 7.**
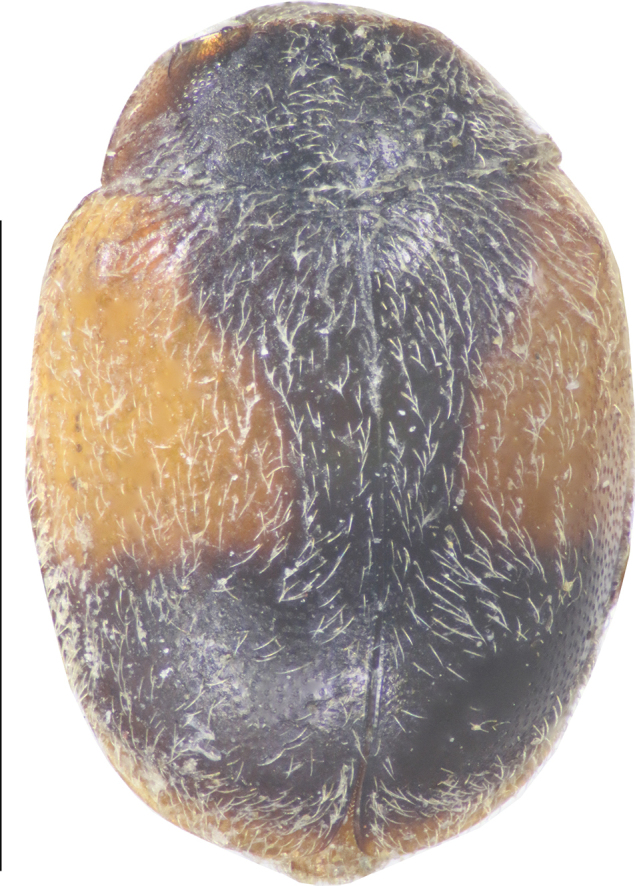
Dorsal view of Scymnus (Scymnus) scapuliferus. Scale bar: 1 mm.

###### Materials examined.

**Asir**: Al Majardah, Wadi Yabah, 19°16.27'N, 41°48.46'E, 411, 2.VI.2012, SN, Al Ansi, A., 2♂2♀; 2.VI.2012, BS, 6♂4♀; Al Majardah, Wadi Khat, 19°05.37'N, 41°58.37'E, 13.III.2012, BS, 1♂; 31.V.2012, BS, 1♂; Wadi Gala, 20°08.08'N, 41°20.56'E, 16.V.2011, SN, Fadl et al., 1♂; **Baha**: Thee Ain, 19°55.78'N, 41°26.60'E, 741 m, 11.V.2011, SN, 4♀; 15.V.2011, SN, 2♂2♀; 10.III.2012, SN, 1♀; 25.IV.2013, BS, Al Ansi et al., 1♂; Al Mikhwah, 19°49.44'N, 41°22.85'E, 430 m, 18.V.2010, SN, Al Dhafer et al., 2♀; Raghadan, 20°34.25'N, 41°45.11'E, 13.V.2011, SN, Fadl et al., 1♀; **Jizan**: Jizan-Ahd Al Masareha Rd., 17°02.28'N, 42°52.38'E, 11.III.2010, LT, Al Dhafer et al., 1♂; 12.III.2010, LT, Al Dhafer et al., 1♀; King Faisal Sport City, 16°48.23'N, 42°59.49'E, 11 m, 20.V.2012, SN, Al Ansi, A., 1♀; Sabya-Hurub Rd., 17°16.94'N, 42°17.54'E, 97 m, 25.V.2012, SN, Al Ansi, A., 1♀.

###### Local distribution.

It was collected from Asir, Baha, and Jizan.

###### World distribution.

This species has been reported from South, Central, and West Africa; Yemen (Raimundo and van Harten 2000); new country record for Saudi Arabia.

#### Stethorini Dobzhansky, 1924

***Stethorus* Weise, 1885**

##### Stethorus gilvifrons

Taxon classificationAnimaliaColeopteraCoccinellidae

(Mulsant, 1850)

3A23C43E-D817-5C66-BBCE-AFDEB19AB21F

Scymnus gilvifrons Mulsant, 1850: 995.

###### Remark.

This species is known as a predator of spider mites (Martin 1972; Abu-Thuraya 1982; Talhouk 1982; Raimundo and van Harten 2000) and is generally found in natural ecosystems.

###### Materials examined.

**Baha**: Wadi Al Zaraeb, 20°03.60'N, 41°23.19'E, 2123 m, 15.V.2010, BS, Sharaf, M., 1ex; Wadi Al Lehian, 20°09.78'N, 41°36.18'E, 1641 m, 25.IV.2013, BS, Al Ansi, A. and Al Harbi, M., 155exs; Al Lieshtah Village 20 km outside of Baha, 20°12.16'N, 41°22.21'E, 2231 m, 26.IV.2013, BS, Al Ansi, A. and Al Harbi, M., 6exs; Baidah Dam, 20°11.88'N, 41°24.06'E, 1880 m, 24.IV.2013, BS, Al Ansi, A. and Al Harbi, M., 1ex; **Eastern Province**: Al Ahsa, 22°17.53'N, 50°40.46'E, 28.XII.1982, Al Shuaaei, M., ANMA, 1ex and **Najran**: Rijla Wadi Najran, 17°31.56'N, 44°13.65'E, 1257 m, 15.I.2013, BS, Al Ansi, A., 4♂126exs; Wadi Shuaib Barran, 17°28.94'N, 44°05.52'E, 1325 m, 16.I.2013, BS, Al Ansi, A., 38exs.

###### Local distribution.

It was collected from Baha, Eastern Province, and Najran and was previously reported by Fürsch (1979) from Asir and Riyadh, by Abu-Thuraya (1982) from Eastern Province and Riyadh, and by Talhouk (1982) from Eastern Province, Riyadh, and Makkah and was listed by Martin (1972).

###### World distribution.

**Asia**: AF, CY, IN, IQ, KA, LE, PA, SA, SY, and TR; **Europe**: AB, AR, CR, BU, FR, GR, and IT; **North Africa**: EG; **ORR** (Kovář 2007) and YE (Raimundo and van Harten 2000); AE (Raimundo et al. 2007).

##### Stethorus endrodyi

Taxon classificationAnimaliaColeopteraCoccinellidae

Fürsch, 1970*

6834BD07-2D51-5BD0-BD51-EC3E2BD42384

Figure 8

Stethorus endrodyi Fürsch, 1970: 98.

###### Diagnosis.

Body is elongated and oval, with dorsal and ventral sides dark, except for the mouth parts, antennae, legs, and frons, which are testaceous; dorsal surface with short, white, semi-erect, not dense pubescence; pronotum is very sparsely and finely punctate (Fürsch 1970).

**Figure 8.**
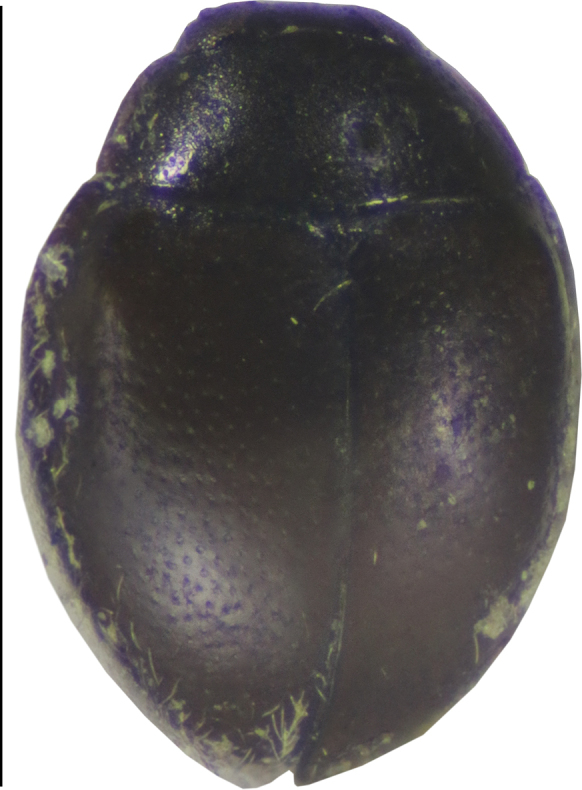
Dorsal view of *Stethorus endrodyi.* Scale bar: 1 mm.

###### Remark.

This is a very rare species, and only one specimen was collected from wild vegetation throughout the study period. It is also not common in the rest of the world.

###### Material examined.

**Asir**: Al Majaredah Wadi Yabah, 19°16.27'N, 41°48.46'E, 411, 2.VI.2012, SN, Al Ansi, A., 1ex.

###### Local distribution.

Individuals of this species were collected from Asir province.

###### World distribution.

YE, Congo, MO, Ivory Coast, Ghana, Angola, and Malawi (Raimundo et al. 2006); new country record for Saudi Arabia.

#### Sticholotidini Weise, 1901

***Pharoscymnus* Bedel, 1906**

##### Pharoscymnus arabicus

Taxon classificationAnimaliaColeopteraCoccinellidae

Fürsch, 1979

E3D6B948-4E2E-5E10-8FC2-4D5CAEE2675D

Pharoscymnus arabicus Fürsch, 1979: 238.

###### Remark.

A rare species that has limited geographical distribution and found only in some countries of the Middle East. It was not collected during the study period.

###### Local distribution.

It was previously reported from Asir by Fürsch (1979).

###### World distribution.

**Asia**: AE, IN, and SA (Kovář 2007).

##### Pharoscymnus c-luteum

Taxon classificationAnimaliaColeopteraCoccinellidae

(Sicard, 1907)

51D3AB08-91D7-57B7-91A2-92CA95234ED9

Pharus c-luteum Sicard, 1907: 417.

###### Remark.

This is a rare species, only known in the Arabian Peninsula, and found as a predator of armoured scale insects (Hemiptera: Diaspididae) (Raimundo and van Harten 2000).

###### Materials examined.

**Asir**: Al Majardah, Wadi Khat, 19°05.37'N, 41°58.37'E, 31.V.2012, BS, Al Dhafer et al., 2exs; Al Majardah, Wadi Al Talalie, 19°05.19'N, 41°47.78'E, 286 m, 1.VI.2012, BS, Al Ansi, A., 1ex; Ahd Rifidh, 18°06.33'N, 42°53.82'E, 16.I.2013, BS, Al Ansi et al., 2exs; Al Majardah, Wadi Yabah, 19°16.27'N, 41°48.46'E, 411, 2.IV.2012, BS, Al Ansi, A., 3♂24exs; 2.IV.2012, LT, Al Ansi, A., 1ex; 2.IV.2012, SN, Al Ansi, A., 1ex; Al Majardah, Wadi Thalooth Al Mandhar, 42°01.12'E, 433 m, 31.V.2012, BS, Al Ansi, A., 6exs; **Baha**: Wadi Turubah, 20°14.37'N, 41°15.23'E, 27.IX.2013, Al Dhafer, H., 2exs; Thee Ain, 19°55.78'N, 41°26.60'E, 741 m, 10.III.2012, BS, Al Dhafer et al., 1ex; 25.IV.2013, BS, Al Ansi et al., 3exs; 3.VI.2012, BS, Al Ansi, A., 3exs; Al Quhman Village, 5 km before Al Atawilah, 20°19.23'N, 41°19.48'E, 2014 m, 24.IV.2013, BS, Al Ansi et al., 2exs; 18 km before Al Atawilah, 20°25.12'N, 41°19.41'E, 1654 m, 24.IV.2013, BS, Al Ansi et al., 2exs; Raghdan, 20°34.25'N, 41°45.11'E, 13.V.2011, SN, Fadl et al., 1ex; Sad Medhas, 20°13.26'N, 41°16.53'E, 1781 m, 9.III.2012, SN, Al Dhfer et al., 4exs; **Jizan**: Hurub Wadi Hurub, 17°43.12'N, 42°57.88'E, 398 m, 24.V.2012, BS, Al Ansi, A., 5exs; Hurub Wadi Qasi, 17°26.52'N, 42°57.32'E, 284 m, 24.V.2012, BS, Al Ansi, A., 1ex.

###### Local distribution.

Specimens of this species were collected from Asir, Baha, and Jizan. It was previously reported by Fürsch (1979) from Asir province.

###### World distribution.

**Asia**: SA and YE (Kovář 2007).

##### Pharoscymnus fleischeri

Taxon classificationAnimaliaColeopteraCoccinellidae

(J. Weise, 1883)*

0AA189EF-DF9F-5E7C-8671-1657A310FDDB

Figure 9

Pharus fleischeri J. Weise, 1883: 67.

###### Remark.

This is a rare species with limited geographical range and found only in the Middle East and Germany. It was attracted to light in the month of May.

###### Material examined.

**Riyadh**: Al Hariq-Ar Raayn Rd., 23°33.88'N, 46°22.53'E, 737 m, 8.V.2012, LT, Al Ansi, A., 1ex.

###### Local distribution.

Riyadh province.

###### World distribution.

**Asia**: IN and TR and newly recorded from SA. **Europe**: GR (Kovář 2007).

**Figure 9.**
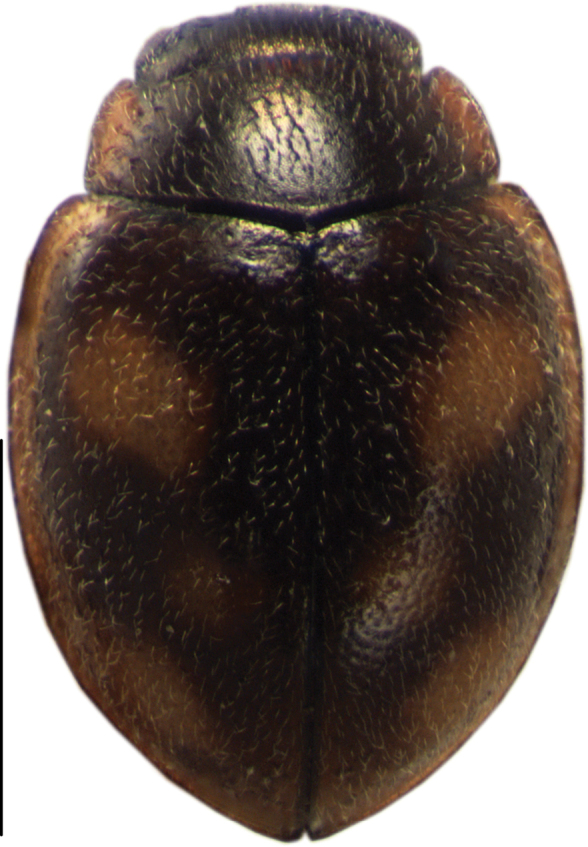
Dorsal view of *Pharoscymnus fleischeri*. Scale bar: 1 mm.

##### Pharoscymnus numidicus

Taxon classificationAnimaliaColeopteraCoccinellidae

(Pic, 1900)

E5CC1ADC-9F6A-5547-A453-378626A00C10

Pharus numidicus Pic, 1900: 91.

###### Remark.

This species was found in the natural ecosystem of the KSA. It is a very good biological control agent and has been introduced into Morocco for the control of *Parlatoria blanchardi* (Targioni Tozzetti, 1892) (Raimundo and van Harten 2000). Specimens were collected using beating sheet.

###### Materials examined.

**Asir**: Al Majardah Wadi Yabah, 19°16.27'N, 41°48.46'E, 411, 2.VI.2012, BS, Al Ansi, A., 1♀13exs; 2.VI.2012, SN, Al Ansi, A., 1ex; 12.III.2012, SN, Al Ansi, A., 2exs; Al Majardah Wadi Thalooth Al Mandhar, 42°01.12'E, 433 m, 31.V.2012, BS, Al Ansi, A., 5exs; Al Majardah, Wadi Qanunah, 19°24.67'N, 41°36.39'E, 348 m, 11.III.2012, SN, Al Dhafer et al., 1♂36exs; Al Majardah, Wadi Baqrah, 18°47.57'N, 42°01.12'E, 433 m, 10.XI.2012, BS, Fadl, H., 2exs; Al Majardah, Wadi Qanunah, 19°24.67'N, 41°36.39'E, 348 m, 11.III.2012, LT, Al Dhafer et al., 1ex; **Baha**: Thee Ain, 19°55.78'N, 41°26.60'E, 741 m, 18.V.2010, BS, Sharaf, M., 1ex; 3.VI.2012, BS, Al Ansi, A., 1ex; **Jizan**: Al Aydabi Wadi Qasi, 17°26.52'N, 42°57.32'E, 284 m, 23.V.2012, BS, Al Ansi, A., 1♀5exs; Hurub Wadi Qasi, 17°26.52'N, 42°57.32'E, 284 m, 24.V.2012, BS, Al Ansi, A., 1♂4exs; Aiban Sabya Rd. Wadi Shahdan, 17°28.27'N, 42°51.19'E, 433 m, 13.XI.2012, BS, Fadl, H., 1♂2exs; Aiban Haqu Fifa, 17°07.95'N, 43°02.59'E, 253 m, 11.XI.2012, BS, Fadl, H., 1ex; Al Aaridah Wadi Al Rad, 17°04.10'N, 43°04.33'E, 192 m, 21.V.2012, BS, Al Ansi, A., 5exs; Hurub Wadi Hurub, 17°43.12'N, 42°57.88'E, 398 m, 24.V.2012, BS, Al Ansi, A., 1ex; Al Aaridah Jizan Dam, 17°02.62'N, 42°59.36'E, 187 m, 21.V.2012, BS, Al Ansi, A., 1ex; Wadi Shuaib Barran, 17°28.94'N, 44°05.52'E, 1325 m, 16.I.2013, BS, Al Ansi et al., 1ex.

###### Local distribution.

Members of this species were collected from Asir, Baha, and Jizan, and it was previously reported by Fürsch (1979) from Asir and Riyadh.

###### World distribution.

**Asia**: PAL, JO, and SA; **North Africa**: AG, EG, LB, and TU; **AFR** (Kovář 2007); AE (Raimundo et al. 2007).

##### Pharoscymnus ovoideus

Taxon classificationAnimaliaColeopteraCoccinellidae

Sicard, 1929

C3D00E1C-25FF-5CE9-9A25-B7DED9C5C902

Pharoscymnus ovoideus Sicard, 1929: 61.

###### Remark.

This species is a predator of scale insects (Martin 1972; Montaigne and Maouloud 1981; Abu-Thuraya 1982).

###### Materials examined.

**Riyadh**: Riyadh, 20.VI.1981, Talhouk et al., ANMA, 3exs, det. Fürsch 1967, 1968; Al Hariq Ar Rayn Rd., 23°33.88'N, 46°22.53'E, 737 m, 8.V.2012, LT, Al Ansi, A., 1ex.

###### Local distribution.

It has been recorded in Riyadh and was previously reported by Abu-Thuraya (1982) and listed by Martin (1972).

###### World distribution.

**Asia**: IN, JO, and SY; **North Africa**: AG, MO, and TU; **AFR** (Kovář 2007), SA (Abu-Thuraya 1982); AE (Raimundo et al. 2007).

##### Pharoscymnus pharoides

Taxon classificationAnimaliaColeopteraCoccinellidae

(Marsuel, 1868)

6C18517F-9F0E-5689-8E70-7F7B65ABAADB

Scymnus pharoides Marsuel, 1868: 215.

###### Remark.

Presence of this species was not confirmed, except in the catalogue of Kovář (2007) with insufficient details about its distribution in the KSA. Moreover, not a single specimen was collected during or after the study period.

###### World distribution.

**Asia**: IN, PAL, SA, SY, and TR; **North Africa**: EG and LB; **AFR** (Kovář 2007).

##### Pharoscymnus setulosus

Taxon classificationAnimaliaColeopteraCoccinellidae

(Chevrolat, 1861)

5E4C76B3-9CD9-590F-B46C-2D284982DD45

Pharus setulosus Chevrolat, 1861: 269.

###### Remark.

No fresh material could be collected to examine this species. Two specimens from the collection of ANMA, collected by R.D. Pope in 1966, were examined. This species was found on a red scale insect, *Aonidiella* sp., according to the information described by R.D. Pope (1966), Martin (1972) and Abu-Thuraya (1982).

###### Material examined.

**Eastern Province**: Hafuf, 25°15'N, 49°32'E, VI.1966, ANMA, 2exs, det. Pope, 1966.

###### Local distribution.

Specimens were collected from the Eastern province of SA by Pope in 1966 and was also reported by Fürsch (1979) from wadi Awsat and Abu-Thuraya (1982) from Eastern Province and was listed by Martin (1972).

###### World distribution.

**Asia**: AE, IN, PAL, JO, and SA; **Europe**: SP; **North Africa**: AG, EG, LB, MO, and TU (Kovář 2007).

##### Pharoscymnus smirnovi

Taxon classificationAnimaliaColeopteraCoccinellidae

Dobzhanskiy, 1927

DFE5A615-F6A2-5C41-9974-A7B241F959D0

Pharoscymnus smirnovi Dobzhanskiy, 1927: 240.

###### Remark.

This is a rare species with limited geographical distribution.

###### Material examined.

**Riyadh**: Riyadh, 18.IV.1978, Talhouk et al., ANMA, 1ex.

###### Local distribution.

Riyadh province.

###### World distribution.

**Asia**: SA; **Europe**: AB, GG (Kovář 2007).

##### Pharoscymnus tristiculus

Taxon classificationAnimaliaColeopteraCoccinellidae

Sicard, 1907

BEAB8C5B-81F0-5A13-A850-E26690CA3B43

Pharus tristiculus Sicard, 1907: 418.

###### Remark.

No fresh material could be collected to examine this species. Two specimens from the collection of ANMA, collected by Pope in 1970, were examined. This species was found on citrus scale insects (Hemiptera) according to the information provided by Pope (1970).

###### Material examined.

**Al Madinah**: Ovaida farm, 24°25.58'N, 39°36.29'E, 15. IX.1969, SN, ANMA, 2exs, on citrus scale insects, det. Pope 1970.

###### Local distribution.

Specimens were collected from Al Madinah and were also reported by Abu-Thuraya (1982) from Al Madinah.

###### World distribution.

**Asia**: YE; **AFR** (Kovář 2007); SA (Abu-Thuraya 1982).

### Microweiseinae Leng, 1920

#### Serangiini Pope, 1962

***Serangium* Blackburn, 1889**

##### Serangium buettikeri

Taxon classificationAnimaliaColeopteraCoccinellidae

Fürsch, 2000

06774390-F486-5184-92F1-D8FC25EF3F1F

Serangium buettikeri Fürsch, 2000 (18): 233.

###### Remark.

This species has been recorded only in the Arabian Peninsula and is a predator of the citrus blackfly *Aleurocanthus woglumi* Ashby, 1915 (Raimundo and van Harten 2000). Individuals were collected from *Ricinus communis* L. using sweep nets.

###### Materials examined.

**Asir**: Wadi Khat, 19°05.37'N, 41°58.37'E, 13.III.2012, BS, Al Dhafer et al., 2exs; **Baha**: Thee Ain, 19°55.78'N, 41°26.60'E, 741 m, 10.III.2012, BS, Al Dhafer et al., 1ex; 11.IV.2016, BS, Al Ansi et al., 1ex; **Najran**: Rijla, Wadi Najran 17°31.56'N, 44°13.65'E, 1257 m, 15.I.2013, BS, Al Ansi, A., 4♂2♀90exs, on *R. communis* L.; 15.I.2013, SN, Al Ansi, A., 1ex, on *R. communis* L.; Wadi Shuaib Barran 17°28.94'N, 44°05.52'E, 1325 m, 16.I.2013, BS, Al Ansi, A., 1ex.

###### Local distribution.

Specimens of this species were reported from Asir, Baha, and Najran provinces and previously reported by Raimundo and van Harten (2000) from Asir.

###### World distribution.

**Asia**: SA and YE (Kovář 2007).

## Discussion

Data on the coccinellid fauna of the KSA are scarce. Fürsch (1979), who provided a key for 35 species, did not provide any precise locality information for eight of the species. Subequently, Kovář (2007) listed 41 species from the KSA but without localities. Before the present study, 56 species were reported in the KSA (Table 2), and the present survey has added eight new records, including *C. lunata lunata*, *C. arcuatus*, *N. ornatulus*, *N. nigricans*, *P. fleischeri*, *R. yemenensis*, *S. scapuliferus*, and *S. endrodyi*, for the country, one new genus record, *Clitostethus*, and presented a list of 65 species. Despite extensive exploration of the country, 14 previously known species were not collected during the present study, which included six species with known localitydata, *N. conjunctus* (from the village Qaaraah near Khamis Mushayt, Wadi Johan Abha, Wadi Maraba, 8–20 km from the Abha-Taif Rd.); *N. fenestratus* (from Wadi Shija; Wadi Hanifah; Wadi Khumra; 28 km Abha-Jizan Rd.; Wadi Ad Dilla; Wadi Mizbil and Salbukh); *S. latemaculatus* (from Khureis); *S. arabicus* (collected Thanomah; 8–20 km from the Abha-Taif Rd.; village Qaaraah near Khamis Mushayt); *P. arabicus* (from Wadi Johan Abha); and *T. arabicus* (from the village Qaaraah near Khamis Mushayt and El Hejdaz), and eight species from unknown localities.

## Supplementary Material

XML Treatment for Brumoides adenensis

XML Treatment for Brumoides nigrosuturalis

XML Treatment for Chilocorus bipustulatus

XML Treatment for Chilocorus distigma

XML Treatment for Parexochomus nigripennis

XML Treatment for Parexochomus nigromaculatus

XML Treatment for Parexochomus pubescens

XML Treatment for Parexochomus sjoestedti

XML Treatment for Tetrabrachys arabicus

XML Treatment for Tetrabrachys minutus

XML Treatment for Tetrabrachys tenebrosus

XML Treatment for Bulaea lividula bocandei

XML Treatment for Cheilomenes lunata lunata

XML Treatment for Cheilomenes lunata yemenensis

XML Treatment for Cheilomenes propinqua vicina

XML Treatment for Coccinella septempunctata

XML Treatment for Coccinella undecimpunctata menetriesi

XML Treatment for Harmonia axyridis

XML Treatment for Hippodamia variegata

XML Treatment for Oenopia oncina

XML Treatment for Psyllobora bisoctonotata

XML Treatment for Xanthadalia effusa rufescens

XML Treatment for Henosepilachna hirta

XML Treatment for Chnootriba elaterii orientalis

XML Treatment for Novius

XML Treatment for Novius argodi

XML Treatment for Novius cardinalis

XML Treatment for Novius yemenensis

XML Treatment for Diomus rubidus

XML Treatment for Hyperaspis polita

XML Treatment for Hyperaspis pumila pumila

XML Treatment for Hyperaspis vinciguerrae

XML Treatment for Clitostethus arcuatus

XML Treatment for Nephus (Bipunctatus) conjunctus

XML Treatment for Nephus (Bipunctatus) nigricans

XML Treatment for Nephus (Bipunctatus) ornatulus

XML Treatment for Nephus (Bipunctatus) wittmeri

XML Treatment for Nephus (Geminosipho) arcuatus

XML Treatment for Nephus (Geminosipho) fenestratus

XML Treatment for Nephus (Nephus) crucifer

XML Treatment for Nephus (Sidis) hiekei

XML Treatment for Nephus (Sidis) levaillanti

XML Treatment for Scymnus (Pullus) agrumi

XML Treatment for Scymnus (Pullus) arabicus

XML Treatment for Scymnus (Pullus) auritus

XML Treatment for Scymnus (Pullus) ebneri

XML Treatment for Scymnus (Pullus) latemaculatus

XML Treatment for Scymnus (Pullus) luxorensis

XML Treatment for Scymnus (Pullus) subvillosus

XML Treatment for Scymnus (Pullus) syriacus

XML Treatment for Scymnus (Pullus) yemenensis

XML Treatment for Scymnus (Scymnus) interruptus

XML Treatment for Scymnus (Scymnus) nubilus

XML Treatment for Scymnus (Scymnus) scapuliferus

XML Treatment for Stethorus gilvifrons

XML Treatment for Stethorus endrodyi

XML Treatment for Pharoscymnus arabicus

XML Treatment for Pharoscymnus c-luteum

XML Treatment for Pharoscymnus fleischeri

XML Treatment for Pharoscymnus numidicus

XML Treatment for Pharoscymnus ovoideus

XML Treatment for Pharoscymnus pharoides

XML Treatment for Pharoscymnus setulosus

XML Treatment for Pharoscymnus smirnovi

XML Treatment for Pharoscymnus tristiculus

XML Treatment for Serangium buettikeri
